# Supplementary material for: Salt-stress-induced tomato sweetening involves an SlSnRK2.6-SlZHD8 sugar accumulation cascade triggered by root-derived abscisic acid
Source: EMBO J. 2026 Feb 2;45(7):2134–56. doi: 10.1038/s44318-026-00708-0 (PMC13043847; doi:10.1038/s44318-026-00708-0)
Supplement: Supplementary file 1 — Appendix [file 44318_2026_708_MOESM1_ESM.pdf]

**Table Of Contents**

Appendix Figure S1. Plant phenotype and key signaling response under different levels of salt stress.

Appendix Figure S2. Influence of exogenous ABA on fruit sugar contents.

Appendix Figure S3. Influence of salt stress on ABA biosynthesis.

Appendix Figure S4. Screening *SlSnRK2.6* as the target for salt-mediated fruit sugar accumulation.

Appendix Figure S5. Screening identifies SlZHD8 as a SlSnRK2.6-interacting protein regulating fruit sugar accumulation during salt stress.

Appendix Figure S6. Identification of the phosphorylated residue of SlZHD8.

Appendix Figure S7. Additional data of *in vivo* phosphorylation.

Appendix Figure S8. Expression patterns of *SlZHD8*.

Appendix Figure S9. Influence of SlSnRK2.6-mediated phosphorylation of SlZHD8 on fruit sugar contents.

Appendix Figure S10. DAP-seq analysis.

Appendix Figure S11. KEGG analysis of RNA-seq.

Appendix Figure S12. Data related to Figure 5.

Appendix Figure S13. Data related to Figure 5.

Appendix Figure S14. Salt tolerance of $ZHD8^{HapA}$ and $ZHD8^{HapB}$.

Appendix Figure S15. Agronomic traits of *ZHD8* mutation and different haplotypes.

Appendix Figure S16. Conservative analysis of ZHD8 homologous proteins and gene expression pattern of *SlSnRK2.6*, *SlZHD8*, *SlSUS3*, and *SlSWEET12*.

Appendix Figure S17. Genome-wide linkage disequilibrium (LD) decay in tomato.

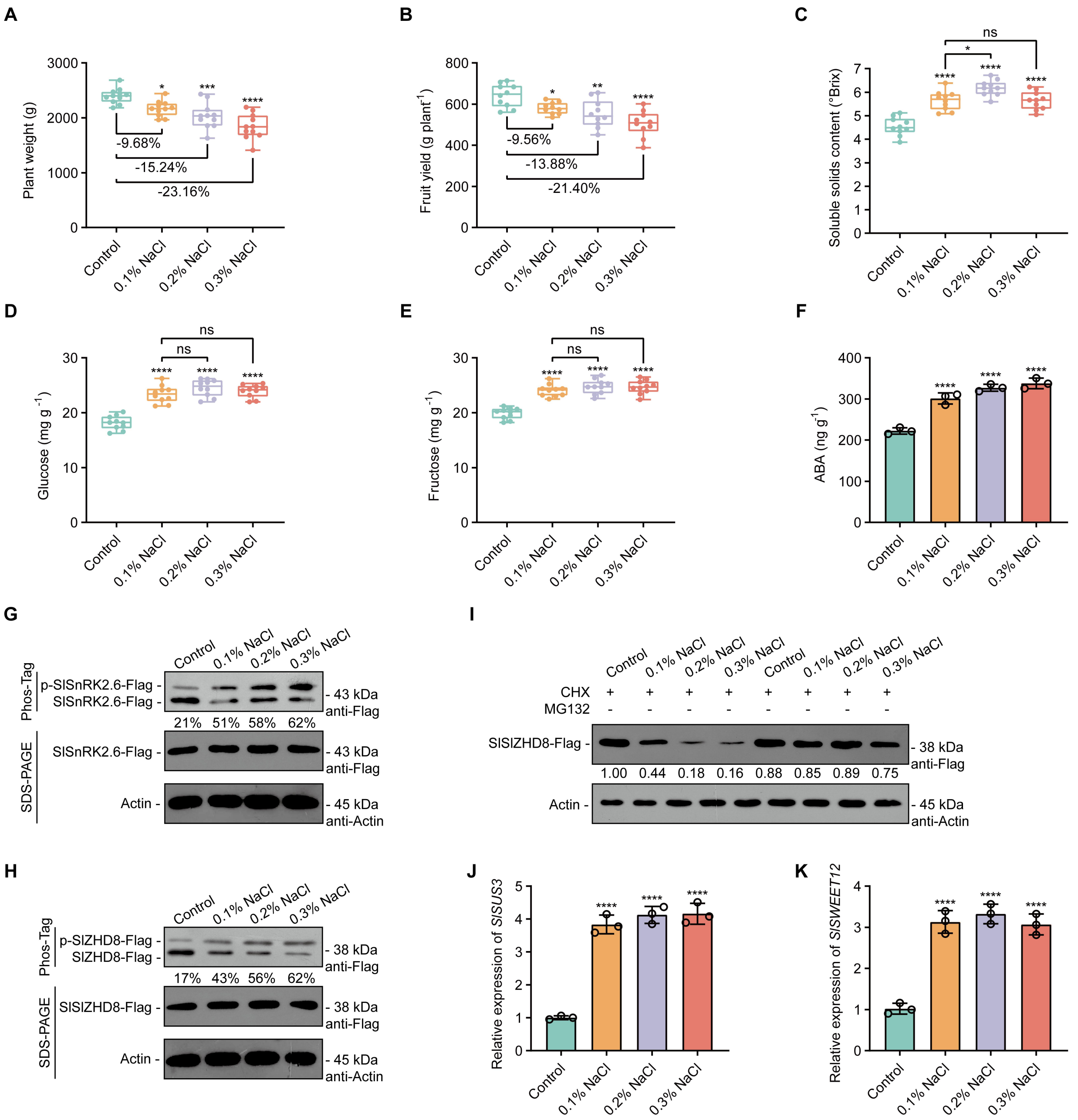


**Appendix Figure S1. Plant phenotype and key signaling response under different levels of salt stress.**

**(A-F)** Plant weight, fruit yield, soluble solids content, glucose content, fructose content, and ABA content. The combined fresh weight of roots, stems, and leaves was recorded as plant weight. Total fruit weight per plant was recorded as fruit yield. Contents of soluble solids, glucose, fructose, and ABA were measured using turning-stage fruits. WT plants were cultivated under control and salt stress (0.1-0.3% NaCl) conditions, with three fruit clusters maintained per plant. Measurements were performed when the second fruit cluster reached the turning stage. Box plots of A to E show median with 0.25 and 0.75 quartiles, whiskers represent values from minimum to maximum (n = 10); data of F represent mean ± SD (n = 3); statistical significance was determined by one-way ANOVA ($^{ns}P > 0.05$, $*P < 0.05$, $**P < 0.01$, $***P < 0.001$, $****P < 0.0001$). **(G)** Kinase activity assay of SlSnRK2.6. Total proteins were extracted from PVX-*SlSnRK2.6-Flag* transgenic fruits in control and salt stress for phosphorylation assay.

Protein abundance and mobility shift were detected by immunoblot and Phos-Tag assay, respectively. The ratio of p-SlSnRK2.6-Flag is shown below the Phos-Tag image. **(H)** Phosphorylation assay of SlZHD8. Total proteins were extracted from PVX-*SlZHD8-Flag* transgenic fruits in control and salt stress for phosphorylation assay. Protein abundance and mobility shift were detected by immunoblot and Phos-Tag assay, respectively. The ratio of p-SlZHD8-Flag is shown below the Phos-Tag image. **(I)** Degradation assay of SlZHD8. Total proteins were extracted from PVX-*SlZHD8-Flag* transgenic fruits in control and salt stress for degradation assay. The cycloheximide (CHX) and MG132 were used as protein synthesis inhibitor and 26S proteasome inhibitor, respectively. Degradation was analyzed by immunoblotting using anti-Flag and anti-Actin (loading control) antibodies, respectively. **(J, K)** Transcription analysis of *SlSUS3* and *SlSWEET12*. Turning-stage fruits in control and salt stress were used for test. Data represent mean ± SD (n = 3); statistical significance was determined by one-way ANOVA (*****P* < 0.0001).

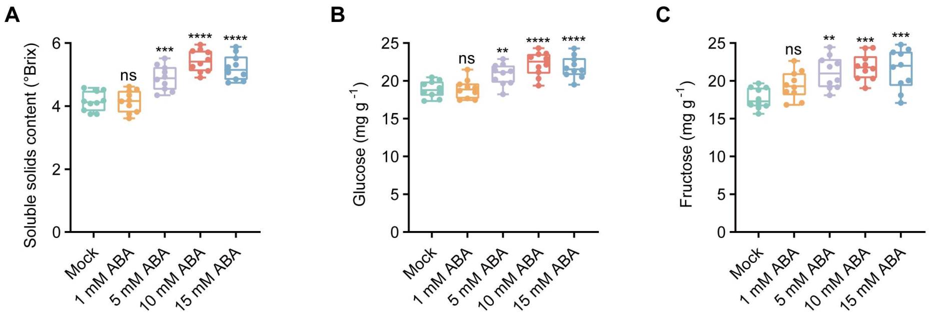


**Appendix Figure S2. Influence of exogenous ABA on fruit sugar contents.**

**(A-C)** Soluble solids content, glucose content, and fructose content. Under normal conditions, fruits at immature green stage were uniformly injected with 25 μL aqueous solution of ABA (1, 5, 10, 15 mM) with the micro-syringe. Distilled water was served as the mock. Measurements were performed when the fruits reached the turning stage. Box plots show median with 0.25 and 0.75 quartiles, whiskers represent values from minimum to maximum (n = 10); statistical significance was determined by one-way ANOVA ($^{ns}P > 0.05$, $^{**}P < 0.01$, $^{***}P < 0.001$, $^{****}P < 0.0001$).

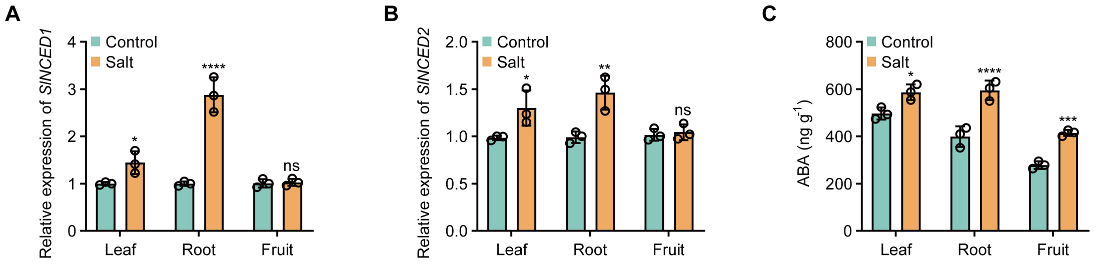


**Appendix Figure S3. Influence of salt stress on ABA biosynthesis.**

**(A-C)** Relative expression of *SlNCED1* and *SlNCED2*, as well as the ABA content. Samples of leaf, root, and turning-stage fruit in control and salt stress were used for test. Data represent mean ± SD (n = 3); statistical significance was determined by two-way ANOVA ($^{ns}P > 0.05$, $^{*}P < 0.05$, $^{**}P < 0.01$, $^{***}P < 0.001$, $^{****}P < 0.0001$).

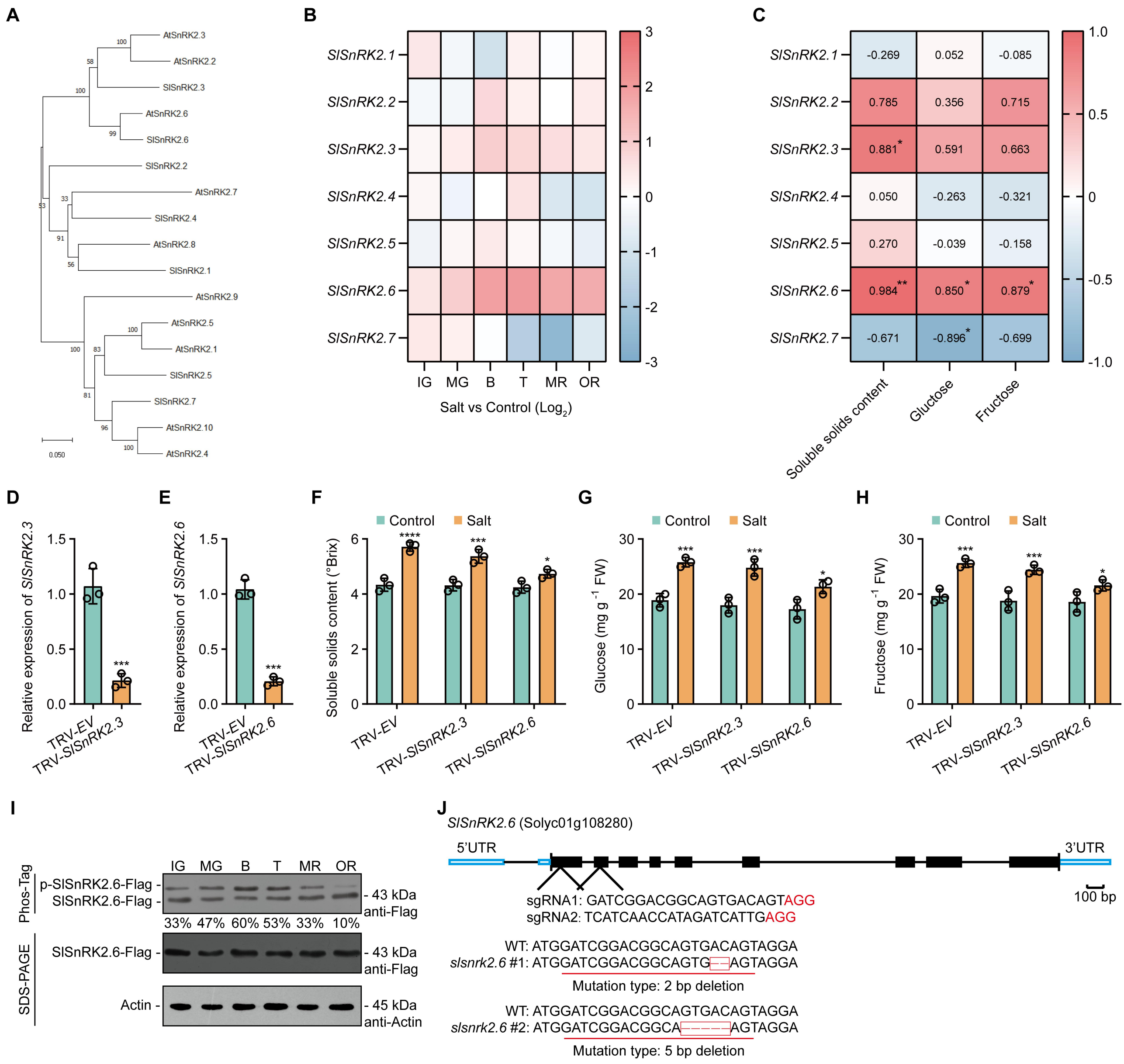


**Appendix Figure S4. Screening *SlSnRK2.6* as the target for salt-mediated fruit sugar accumulation.**

**(A)** Phylogenetic analysis of SlSnRK2s and AtSnRK2s proteins with the neighbor-joining method by MEGA 11. **(B)** Transcriptional response of *SlSnRK2s* genes to salt stress during fruit ripening (immature green, IG; mature green, MG; breaker, B; turning, T; mature red, MR; over red, OR). Data were switched to $\log_2$ transformation to generate the heatmap. Data represent mean (n = 3). **(C)** Correlation analysis of *SlSnRK2s* expression and sugar contents. The original data were derived from Appendix Figure S4B and Figure 1D-F. **(D, E)** Efficient silencing of *SlSnRK2.3* and *SlSnRK2.6* by VIGS. Data represent mean ± SD (n = 3); statistical significance was determined by *t*-text (***$P$ < 0.001). **(F-H)** The interplay between *SlSnRK2.3/2.6* and salt stress affecting soluble solids content, glucose content, and fructose content. Turning-stage fruits of TRV-*EV* (empty vector control), TRV-*SlSnRK2.3*, and TRV-*SlSnRK2.6* in control and salt stress were used for test. Data represent mean ± SD (n = 3); statistical significance was determined by two-way ANOVA (*$P$ < 0.05, ***$P$ < 0.001, ****$P$ < 0.0001). **(I)** Kinase activity assay of SlSnRK2.6

during fruit ripening (immature green, IG; mature green, MG; breaker, B; turning, T; mature red, MR; over red, OR). Total proteins were extracted from PVX-mediated *SlSnRK2.6-Flag* transgenic fruits for phosphorylation assay. Protein abundance and mobility shift were detected by immunoblot and Phos-Tag assay, respectively. The ratio of p-SlSnRK2.6-Flag is shown below the Phos-Tag image. **(J)** CRISPR-Cas9-mediated mutagenesis of *SlSnRK2.6*. Schematic of sgRNA target sites and sequence alignments comparing WT and mutant alleles.

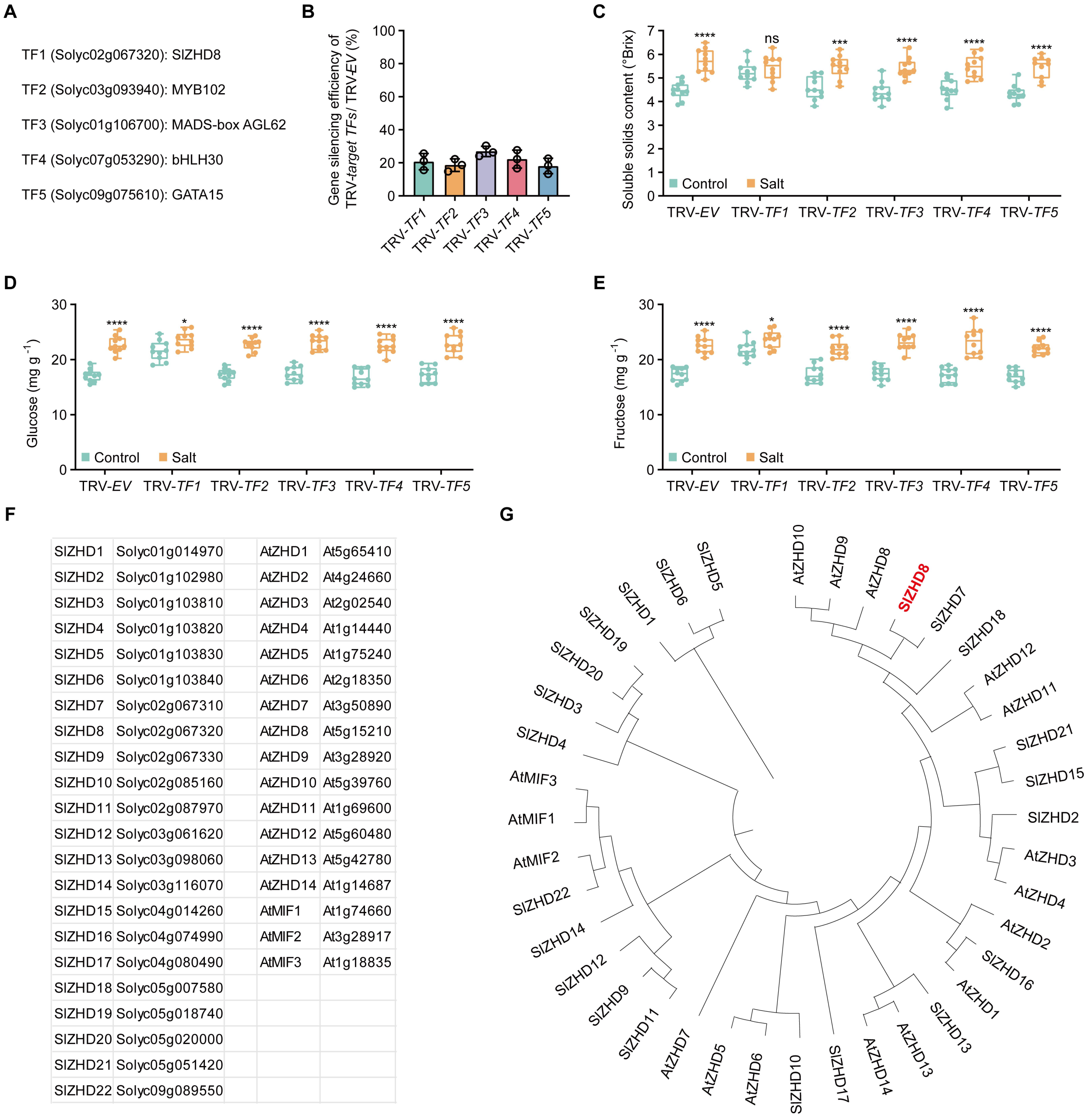


**Appendix Figure S5. Screening identifies SlZHD8 as a SlSnRK2.6-interacting protein regulating fruit sugar accumulation during salt stress.**

**(A)** Information of five transcription factors identified in Y2H screen. **(B)** Gene silencing of five transcription factors by VIGS. Data represent mean ± SD (n = 3). **(C-E)** The interplay between five transcription factors and salt stress affecting soluble solids content, glucose content, and fructose content. Turning-stage fruits of TRV-*EV* (empty vector control), TRV-*TF1* to TRV-*TF5* in control and salt stress were used for test. Box plots show median with 0.25 and 0.75 quartiles, whiskers represent values from minimum to maximum (n = 10); statistical significance was determined by two-way ANOVA ($^{ns}P > 0.05$, $^{*}P < 0.05$, $^{***}P < 0.001$, $^{****}P < 0.0001$). **(F, G)** Phylogenetic analysis of SlZHDs and AtZHDs proteins with the neighbor-joining method by MEGA 11.

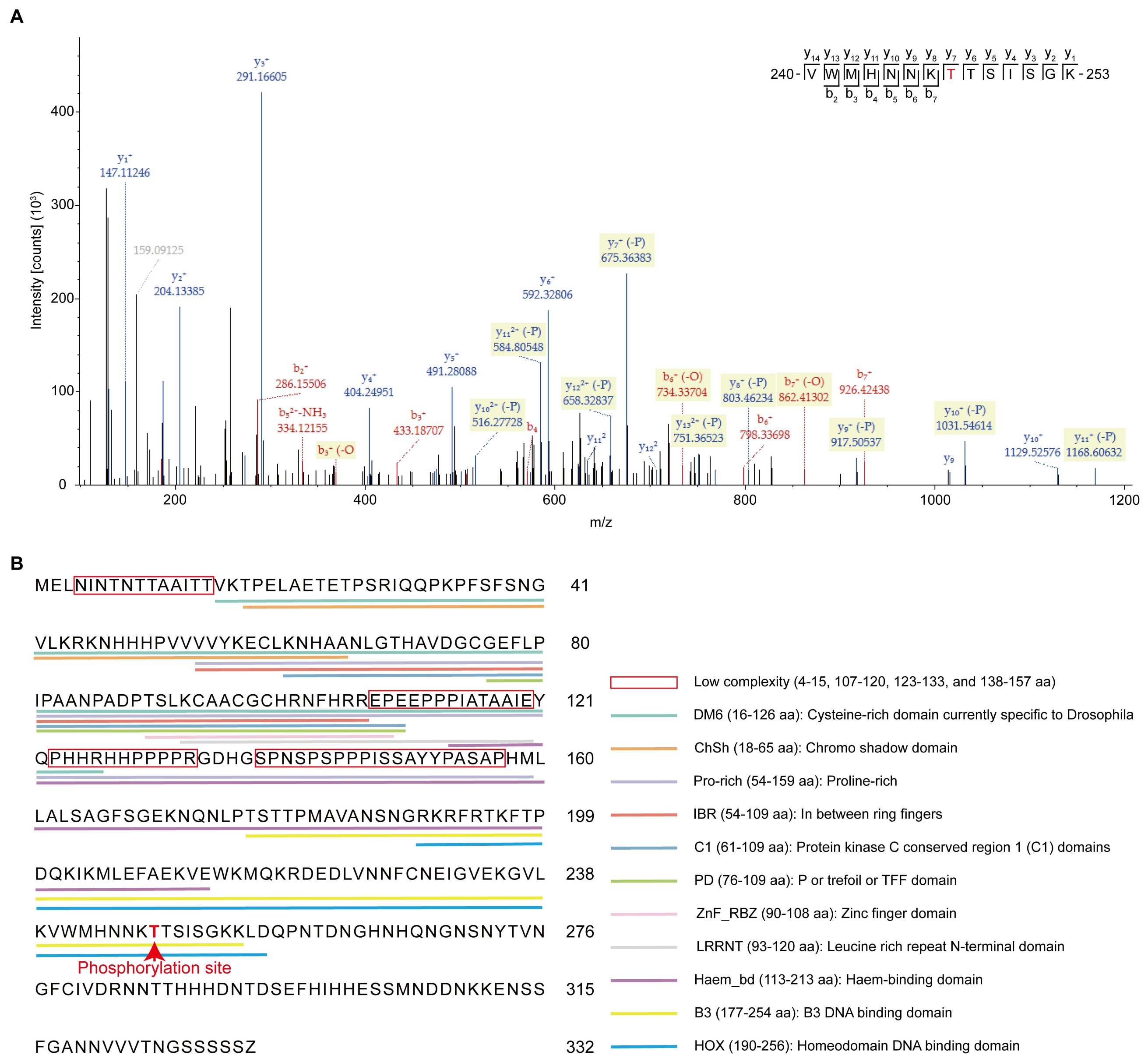


**Appendix Figure S6. Identification of the phosphorylated residue of SlZHD8.**

**(A)** LC-MS/MS analysis revealed that SlZHD8 was phosphorylated by SlSnRK2.6 at T247 residue *in vitro*. **(B)** Protein domain analysis of SlZHD8. Domain analysis was performed by the website (http://smart.emble.de), and each different colored horizontal line represents a domain region. The red font represents the phosphorylated residue (T247).

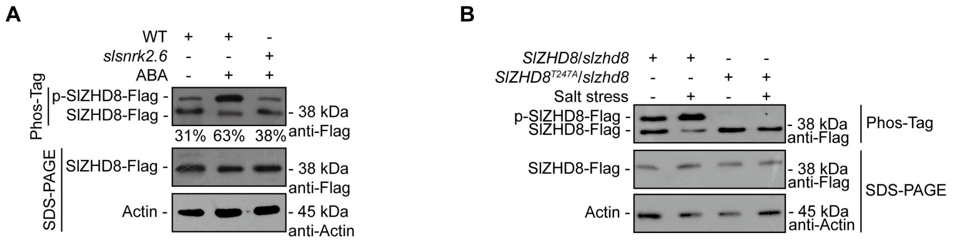


**Appendix Figure S7. Additional data of *in vivo* phosphorylation.**

**(A)** SlSnRK2.6 is involved in ABA-mediated *in vivo* phosphorylation of SlZHD8. Total proteins were extracted from PVX-mediated *SlZHD8-Flag* transgenic fruits of WT and *slsnrk2.6* backgrounds in control and ABA treatments for phosphorylation assay. Protein abundance and mobility shift were detected by immunoblot and Phos-Tag assay, respectively. The ratio of p-SlZHD8-Flag is shown below the Phos-Tag image. **(B)** Salt stress promotes *in vivo* phosphorylation of SlZHD8 at T247 residue. Total proteins were extracted from PVX-mediated *SlZHD8-Flag* or *SlZHD8*$^{T247A}$*-Flag* transgenic fruits of WT plants for phosphorylation assay. Protein abundance and mobility shift were detected by immunoblot and Phos-Tag assay, respectively. The ratio of p-SlZHD8-Flag is shown below the Phos-Tag image.

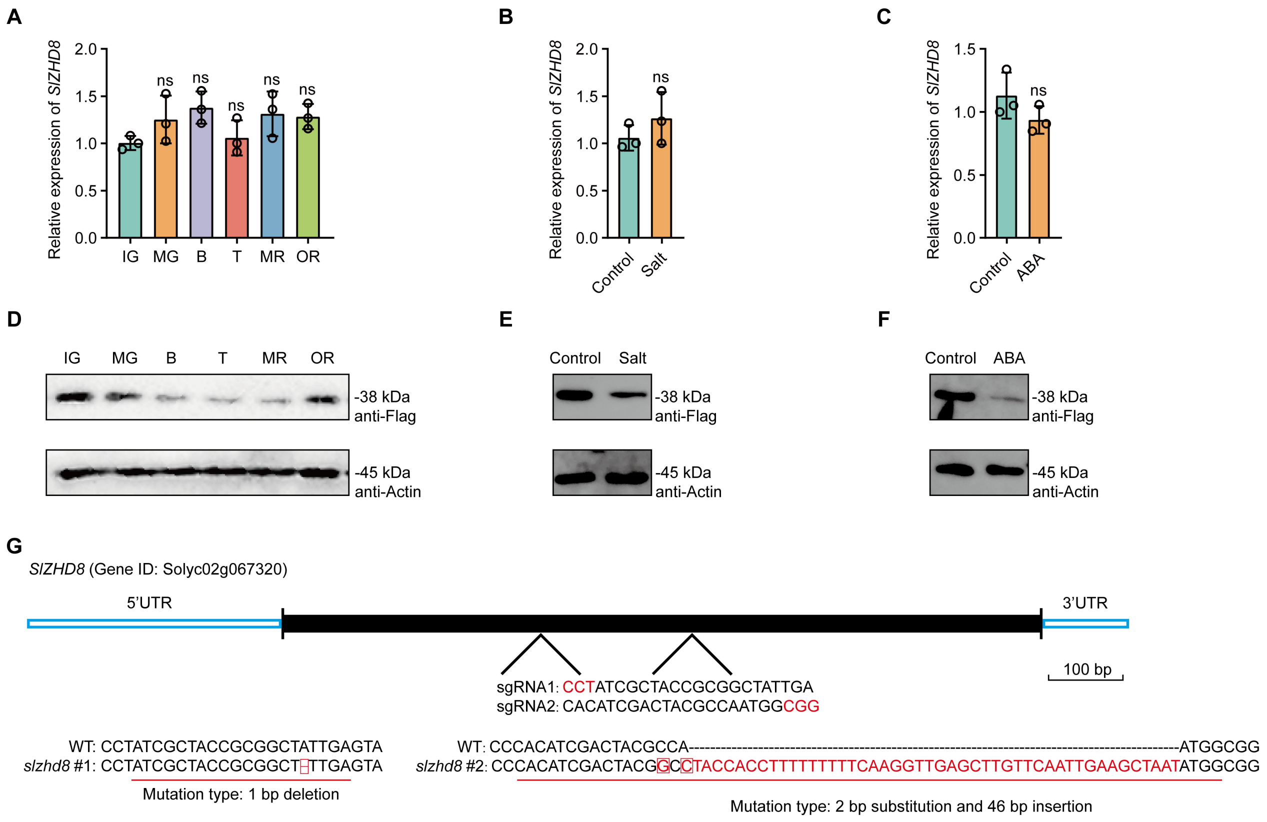


**Appendix Figure S8. Expression patterns of *SlZHD8*.**

**(A)** Transcriptional response of *SlZHD8* during fruit ripening (immature green, IG; mature green, MG; breaker, B; turning, T; mature red, MR; over red, OR). Data represent mean ± SD (n = 3); statistical significance was determined by one-way ANOVA ($^{ns}P$ > 0.05). **(B)** Transcriptional response of *SlZHD8* to salt stress. Turning-stage fruits in control and salt treatments were used for test. Data represent mean ± SD (n = 3); statistical significance was determined by one-way ANOVA ($^{ns}P$ > 0.05). **(C)** Transcriptional response of *SlZHD8* to ABA treatment. Fruits at IG stage were treated with 10 mM ABA. Turning-stage fruits in control and ABA treatments were used for test. Data represent mean ± SD (n = 3); statistical significance was determined by one-way ANOVA ($^{ns}P$ > 0.05). **(D-F)** The response of SlZHD8 protein expression to ripening, salt stress, and ABA treatment. PVX-SlZHD8-Flag was used for the expression of the SlZHD8-Flag protein. The remaining processing and sampling methods are the same as those for gene expression. Protein expression was analyzed by immunoblotting using anti-Flag and anti-Actin (loading control) antibodies, respectively. **(G)** CRISPR-Cas9-mediated mutagenesis of *SlZHD8*. Schematic of sgRNA target sites and sequence alignments comparing WT and mutant alleles.

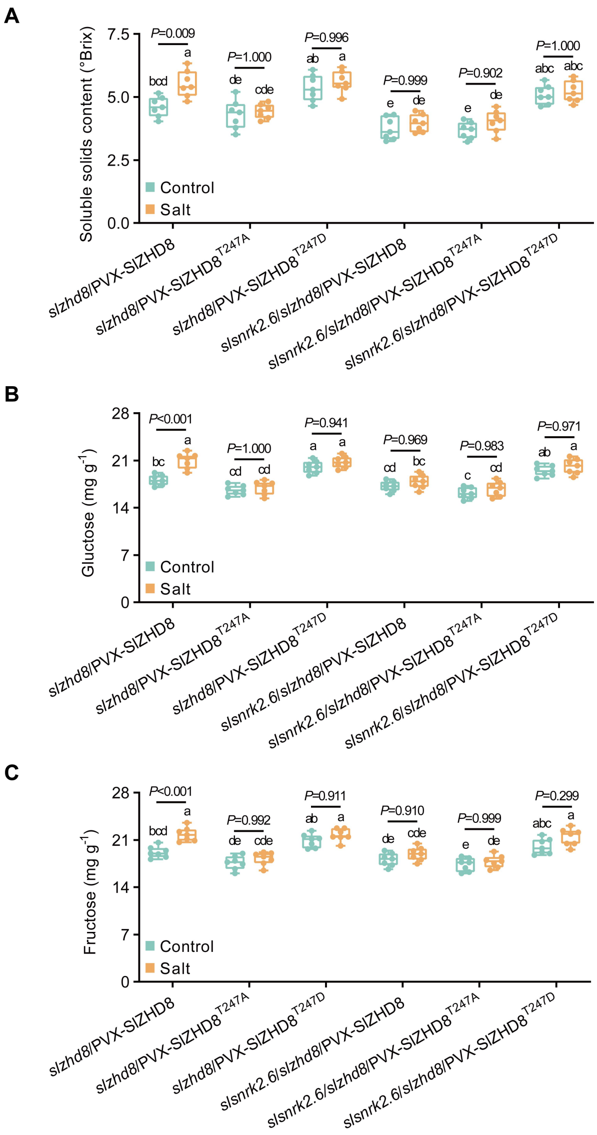


**Appendix Figure S9. Influence of SlSnRK2.6-mediated phosphorylation of SlZHD8 on fruit sugar contents.**

**(A-C)** Soluble solids content, glucose content, and fructose content. Turning-stage fruits of PVX-*SlZHD8*, PVX-*SlZHD8*$^{T247A}$ and PVX-*SlZHD8*$^{T247D}$ in both *slzhd8* and *slsnrk2.6/slzhd8* backgrounds under control and salt stress were used for test. Box plots show median with 0.25 and 0.75 quartiles, whiskers represent values from minimum to maximum (n = 7); statistical significance was determined by one-way ANOVA (Different letters indicate significant differences at $P < 0.05$).

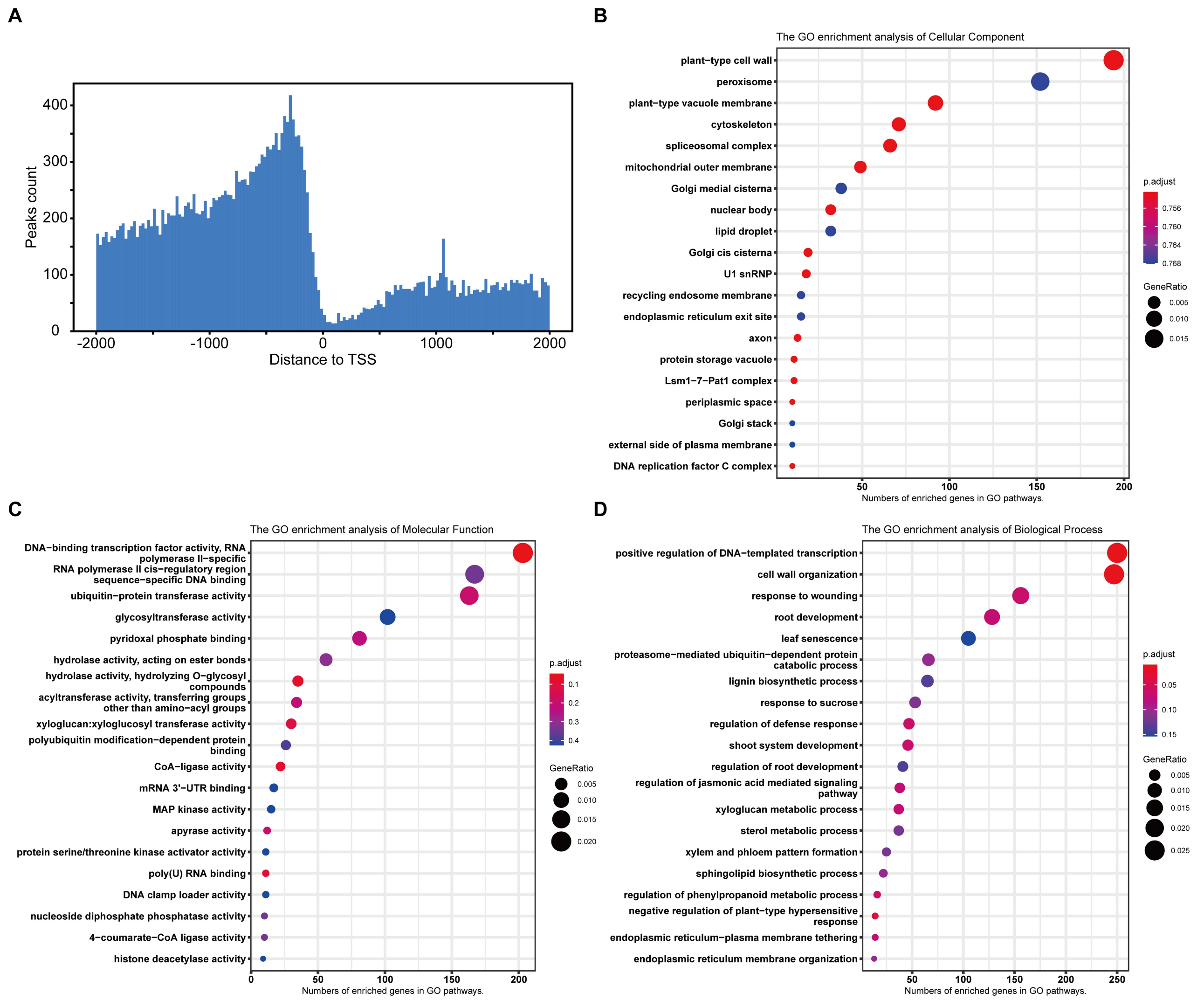


**Appendix Figure S10. DAP-seq analysis.**

**(A)** SlZHD8-binding peaks are highly enriched in the 300-bp region immediately upstream of the TSS. The overlapping peaks were used for analysis. Refer to Dataset EV2. **(B-D)** The GO enrichment analysis of cellular component, molecular function, and biological process of SlZHD8-binding peaks within promoter regions. GO enrichment analysis of the target genes was significant at $P < 0.05$ relative to the genomic background. Refer to Dataset EV4.

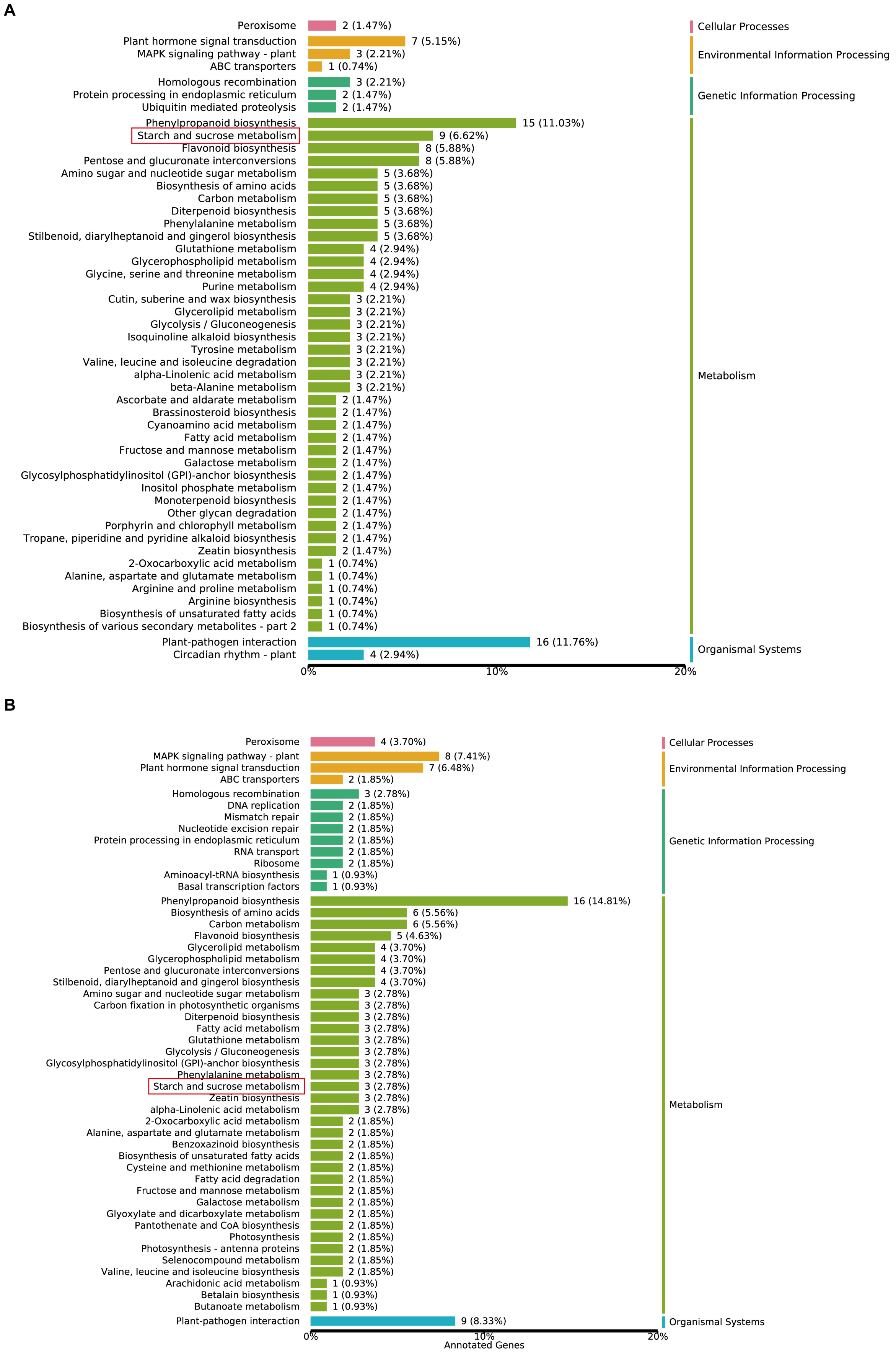


**Appendix Figure S11. KEGG analysis of RNA-seq.**

**(A)** Turning-stage fruit sample (WT vs *slzhd8*) with a significance threshold set at $P < 0.05$. Refer to Dataset S7. **(B)** Root sample (WT vs *slzhd8*) with a significance threshold set at $P < 0.05$.

Refer to Dataset S7.

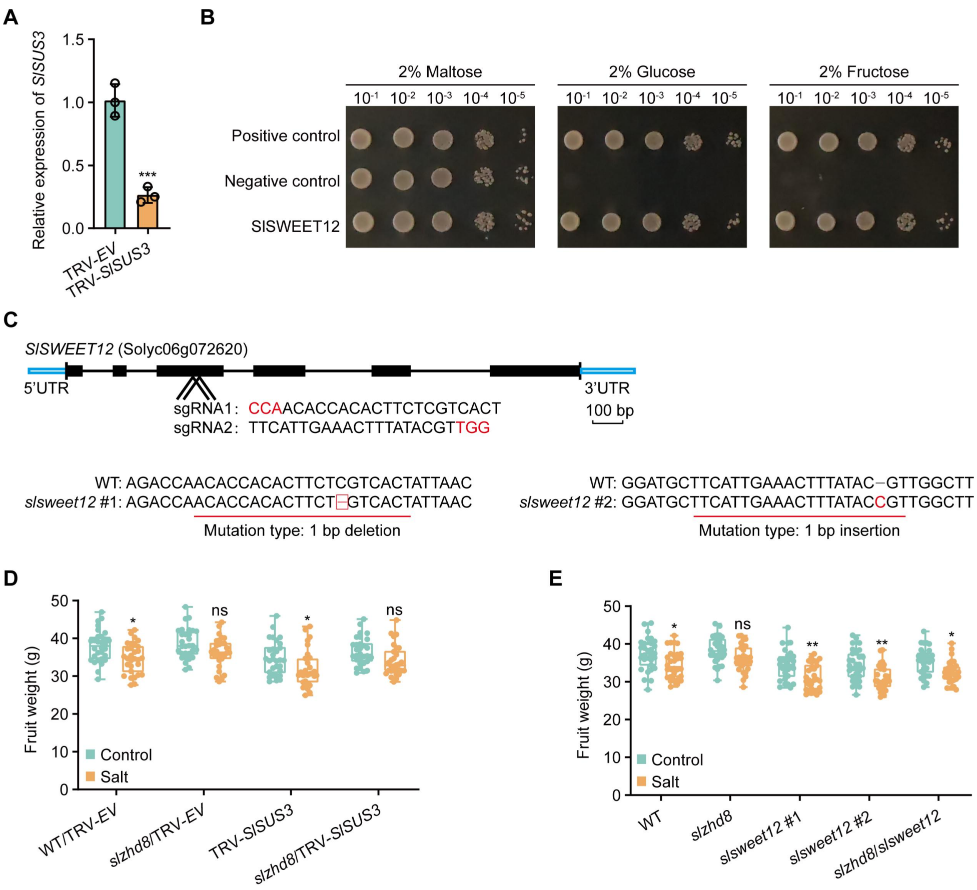


**Appendix Figure S12. Data related to Figure 5.**

**(A)** Efficient silencing of *SlSUS3* by VIGS. Data represent mean ± SD (n = 3); statistical significance was determined by *t*-text (****P* < 0.001). **(B)** Expression of *SlSWEET12* within the hexose uptake-deficient strain EBY4000 cultivated on media containing different sugars (2% maltose, 2% glucose, and 2% fructose). A panel of 2% maltose and yeast HT5 were used as a positive control, while an empty vector was used as a negative control. **(C)** CRISPR-Cas9-mediated mutagenesis of *SlSWEET12*. Schematic of sgRNA target sites and sequence alignments comparing WT and mutant alleles. **(D, E)** Data of fruit weight related to Figure 5A-F. Box plots show median with 0.25 and 0.75 quartiles, whiskers represent values from minimum to maximum (n = 30); statistical significance was determined by two-way ANOVA ($^{ns}P$ > 0.05, **P* < 0.05, ***P* < 0.01).

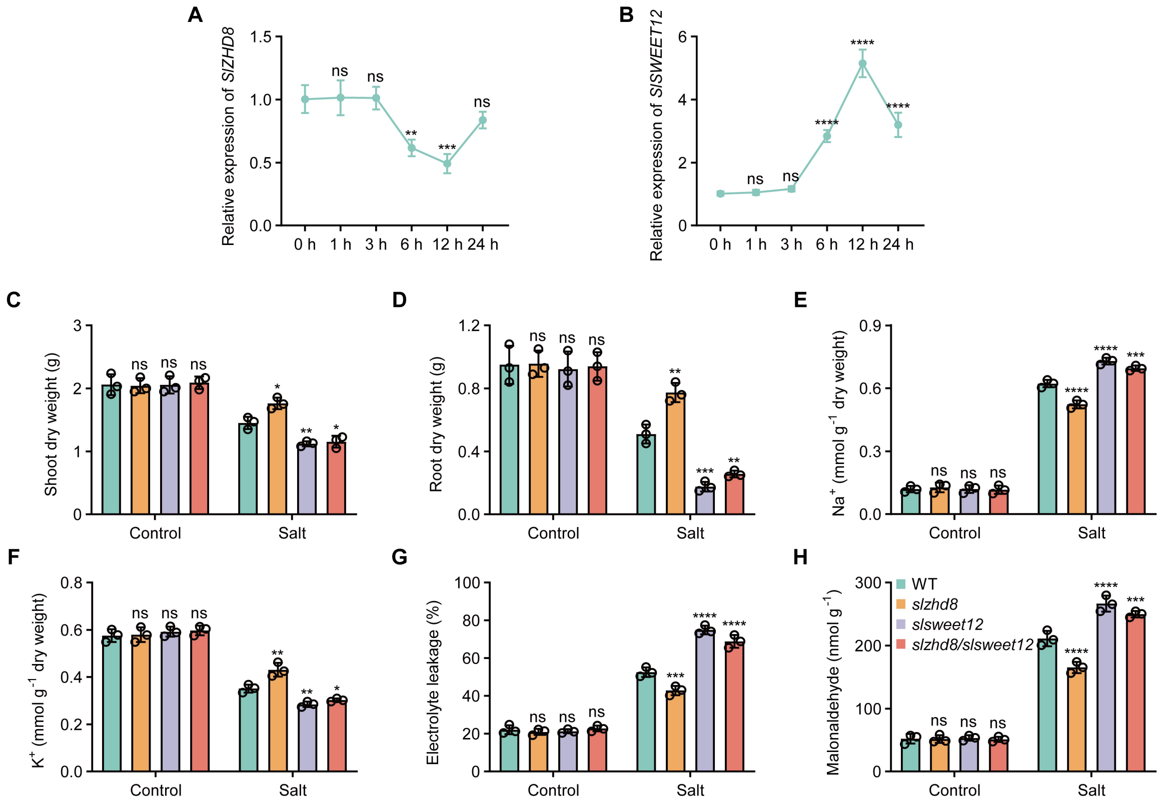


**Appendix Figure S13. Data related to Figure 5.**

**(A, B)** Transcriptional response of *SlZHD8* and *SlSWEET12* to salt stress (0.6% NaCl). Root samples were used for test. Data represent mean ± SD (n = 3); statistical significance was determined by one-way ANOVA ($^{ns}P > 0.05$, $^{*}P < 0.05$, $^{**}P < 0.01$, $^{***}P < 0.001$, $^{****}P < 0.0001$).

**(C-H)** The *SlZHD8-SlSWEET12* module is involved in salt stress tolerance. Shoot dry weight, root dry weight, $Na^+$ content of root, $K^+$ content of root, electrolyte leakage of root, and malonaldehyde content of root. Seedlings of WT, *slzhd8*, *slsweet12*, and *slzhd8*/*slsweet12* were used for test at 20 d after salt stress (0.6% NaCl). Data represent mean ± SD (n = 3); statistical significance was determined by two-way ANOVA ($^{ns}P > 0.05$, $^{*}P < 0.05$, $^{**}P < 0.01$, $^{***}P < 0.001$, $^{****}P < 0.0001$).

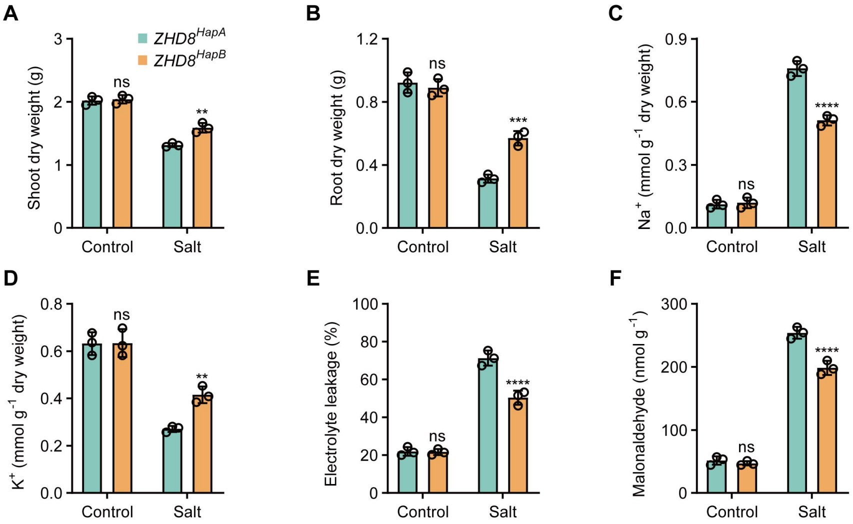


**Appendix Figure S14. Salt tolerance of *ZHD8$^{HapA}$* and *ZHD8$^{HapB}$*.**

**(A)** Shoot dry weight. **(B)** Root dry weight. **(C)** $Na^+$ content of root. **(D)** $K^+$ content of root. **(E)** Electrolyte leakage of root. **(F)** Malonaldehyde content of root. Seedlings of *ZHD8$^{HapA}$* and *ZHD8$^{HapB}$* were used for test at 20 d after salt stress (0.6% NaCl). Data represent mean ± SD (n = 3); statistical significance was determined by two-way ANOVA ($^{ns}P > 0.05$, $^{*}P < 0.05$, $^{**}P < 0.01$, $^{***}P < 0.001$, $^{****}P < 0.0001$).

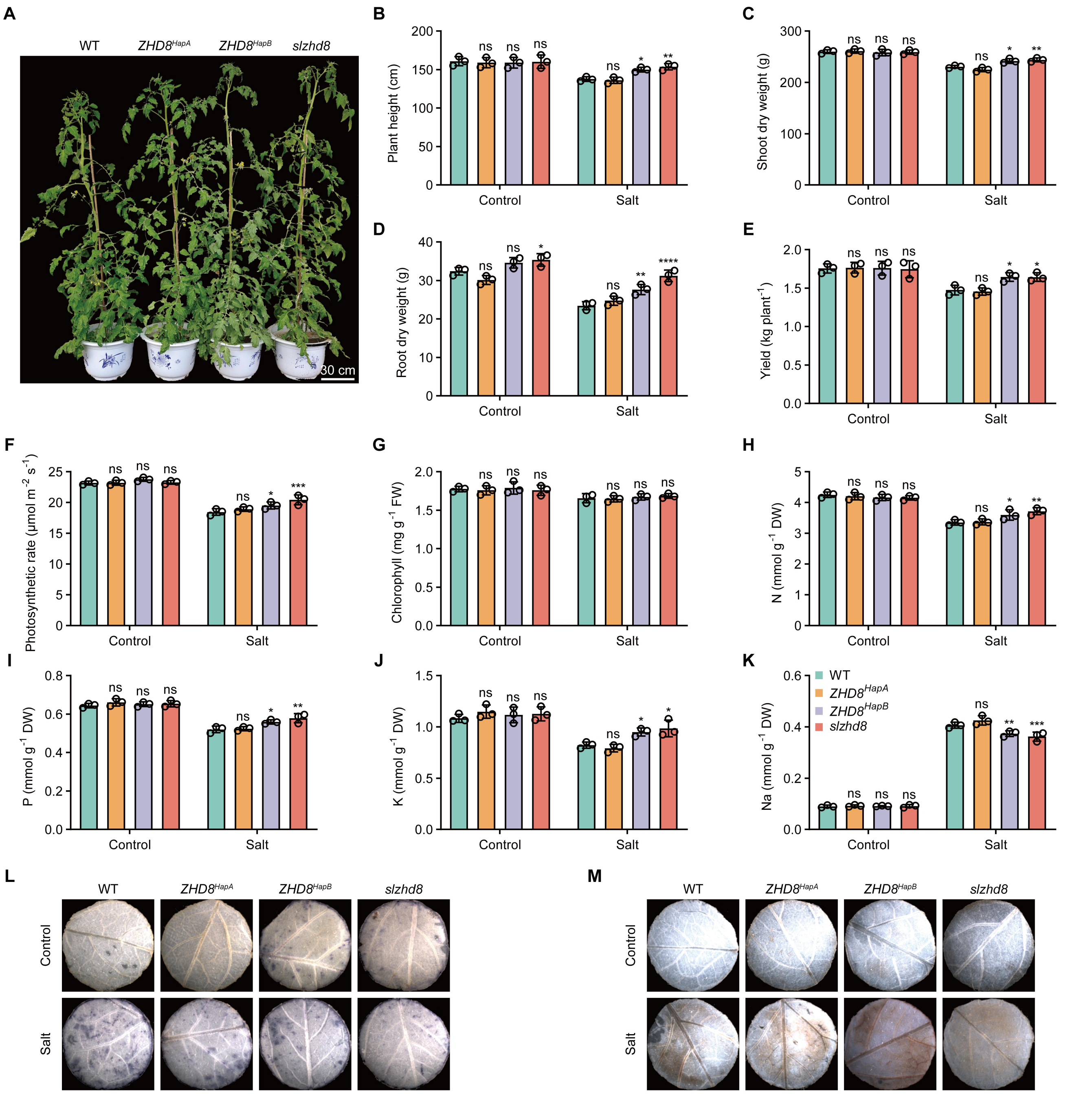


**Appendix Figure S15. Agronomic traits of *ZHD8* mutation and different haplotypes.**

**(A)** Phenotype of plants under salt stress. **(B)** Plant height. **(C)** Shoot dry weight. **(D)** Root dry weight. **(E)** Fruit yield per plant. **(F)** Net photosynthetic rate of the leaves. **(G)** Chlorophyll content of the leaves. **(H)** Nitrogen content of leaves. **(I)** Phosphorus content of leaves. **(J)** Potassium content of leaves. **(K)** Sodium content of leaves. **(L)** Hydrogen peroxide staining by 3,3'-diaminobenzidine. **(M)** Superoxide anion staining by nitroblue tetrazolium. WT, $ZHD8^{HapA}$, $ZHD8^{HapB}$, and *slzhd8* plants were cultivated under control and salt stress (0.1% NaCl) conditions. Measurements were performed at three month after stress. Data represent mean ± SD (n = 3); statistical significance was determined by two-way ANOVA ($^{ns}P > 0.05$, $^{*}P < 0.05$, $^{**}P < 0.01$, $^{***}P < 0.001$, $^{****}P < 0.0001$).

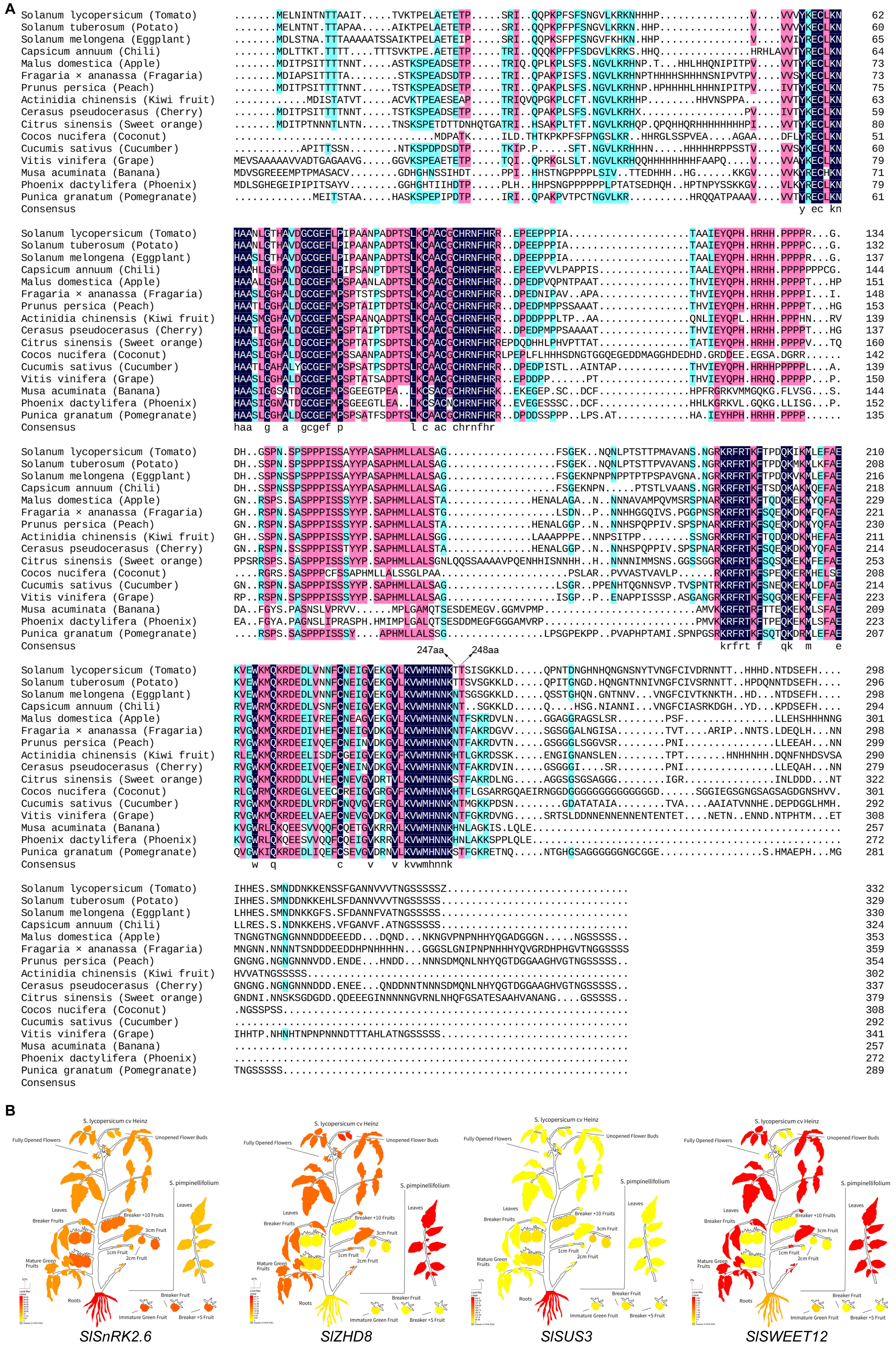


**Appendix Figure S16. Conservative analysis of ZHD8 homologous proteins and gene**

**expression pattern of *SlSnRK2.6*, *SlZHD8*, *SlSUS3*, and *SlSWEET12*.**

**(A)** ZHD8 homologous proteins across 16 fruit-bearing crops. **(B)** Gene expression pattern of *SlSnRK2.6*, *SlZHD8*, *SlSUS3*, and *SlSWEET12*. Data was obtained from the Plant eFP Viewer (http://bar.utoronto.ca/eplant_tomato/).

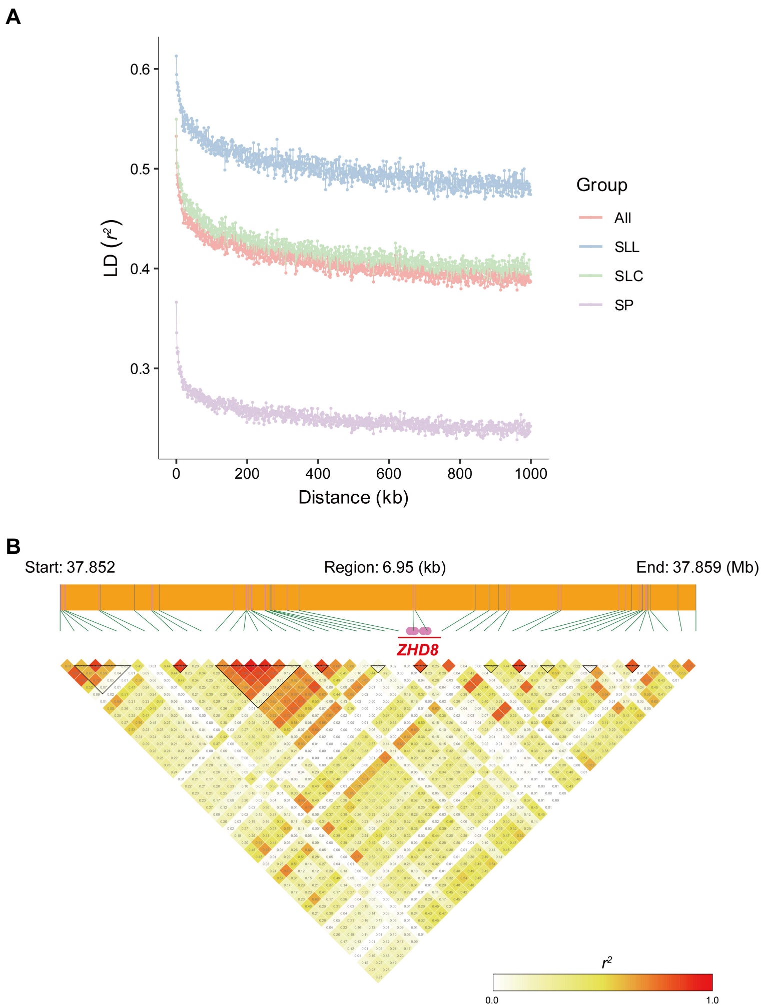


**Appendix Figure S17. Genome-wide linkage disequilibrium (LD) decay in tomato. (A)** The decay of linkage disequilibrium (LD) was evaluated based on single nucleotide polymorphism (SNP) derived from the three tomato population (SP, SLC and SLL). **(B)** LD plot for SNPs located in the 37.852-37.859 Mb region. ZHD8, highlighted in red, did not belong to the continuous association block with other loci. The color gradient from white to red indicates the LD value ($r^2$) among accessions.
