# Supplementary figures and images for: Salt-stress-induced tomato sweetening involves an SlSnRK2.6-SlZHD8 sugar accumulation cascade triggered by root-derived abscisic acid
Source: EMBO J. 2026 Feb 2;45(7):2134–56. doi: 10.1038/s44318-026-00708-0 (PMC13043847; doi:10.1038/s44318-026-00708-0)

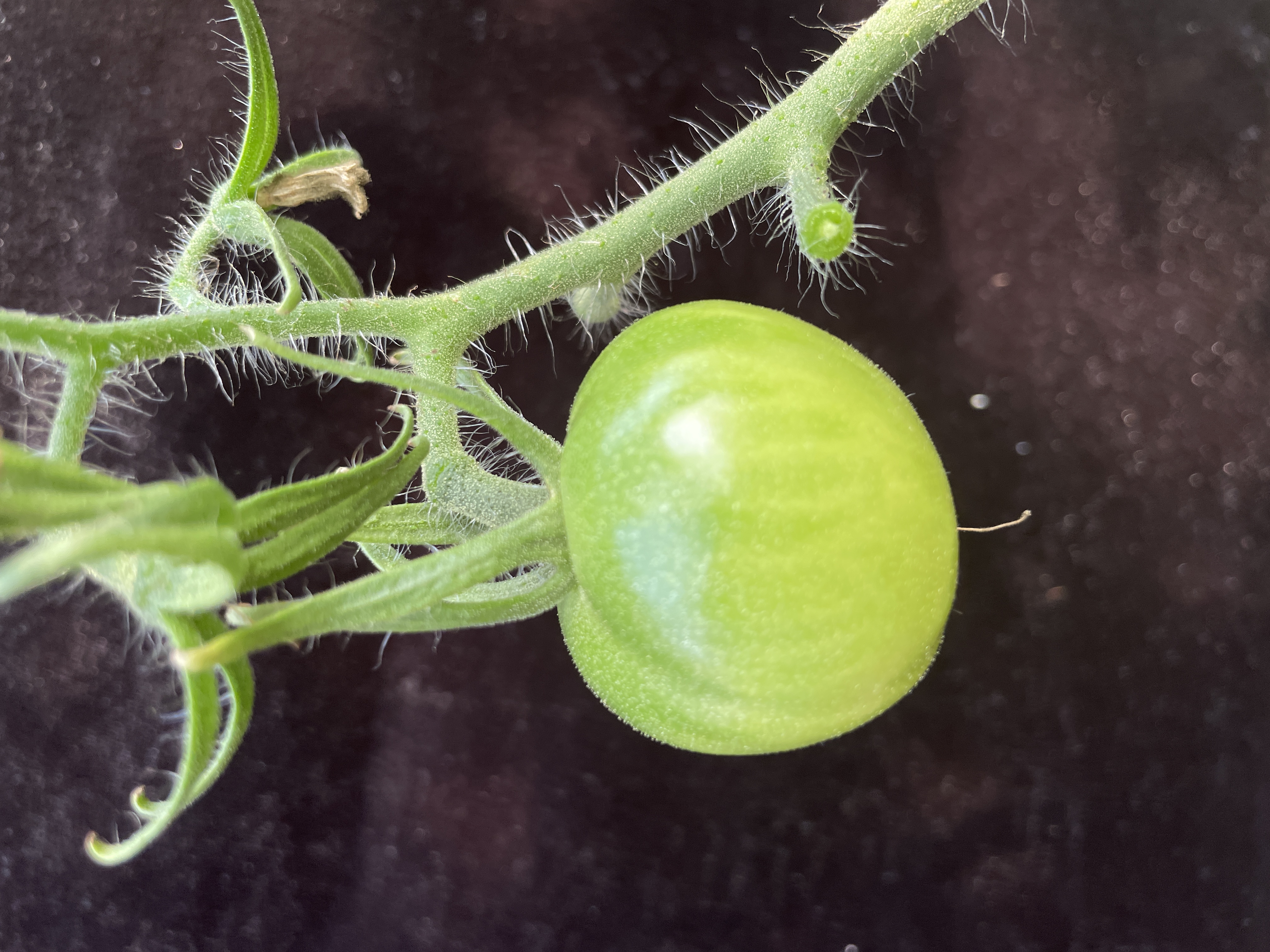

Supplement: Supplementary file 13 — Source data Fig. 1 [file 44318_2026_708_MOESM13_ESM.zip › Source Data Fig 1/Source Data Fig 1A/Control-32dpa.JPG]

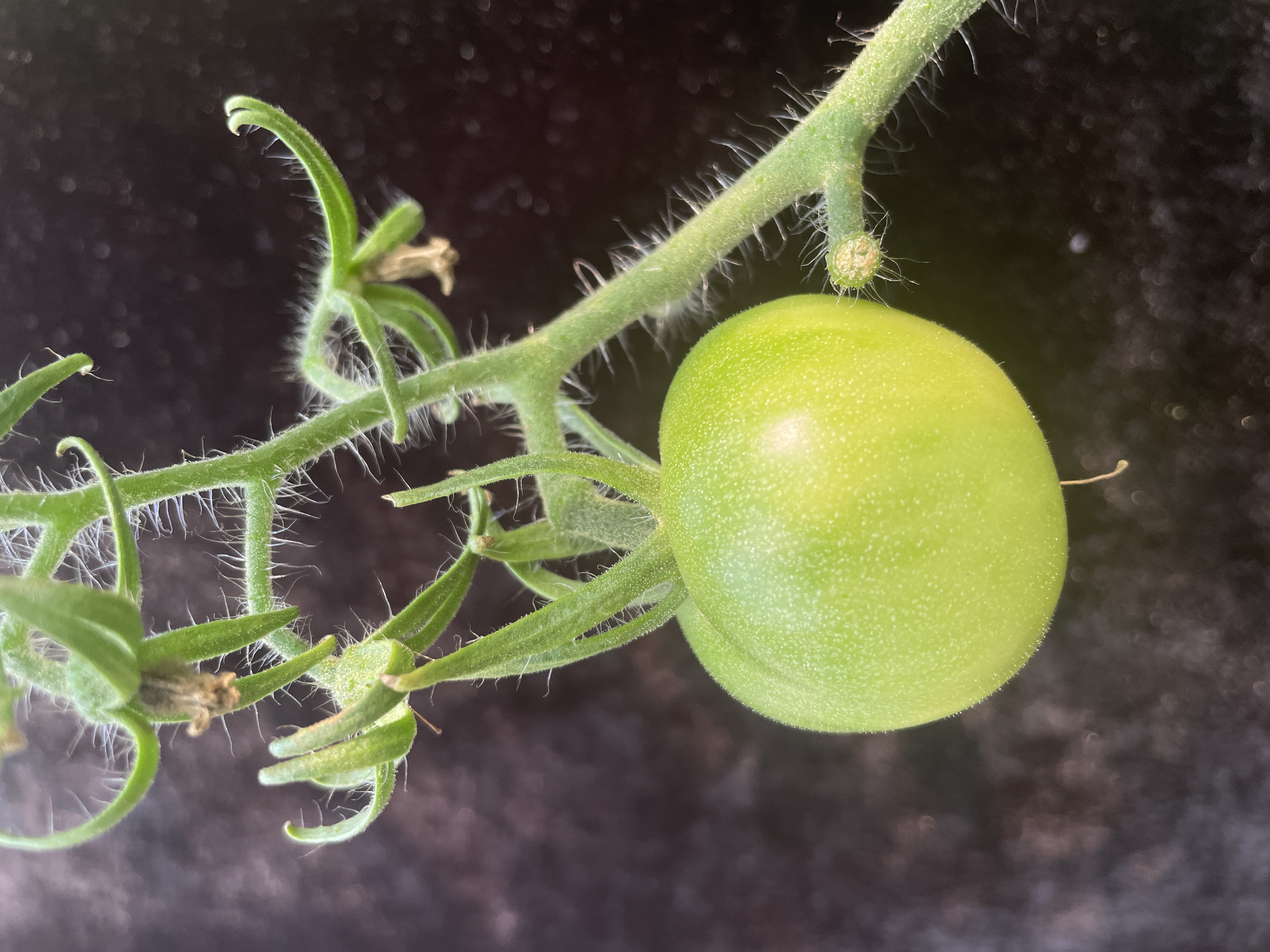

Supplement: Supplementary file 13 — Source data Fig. 1 [file 44318_2026_708_MOESM13_ESM.zip › Source Data Fig 1/Source Data Fig 1A/Control-36dpa.JPG]

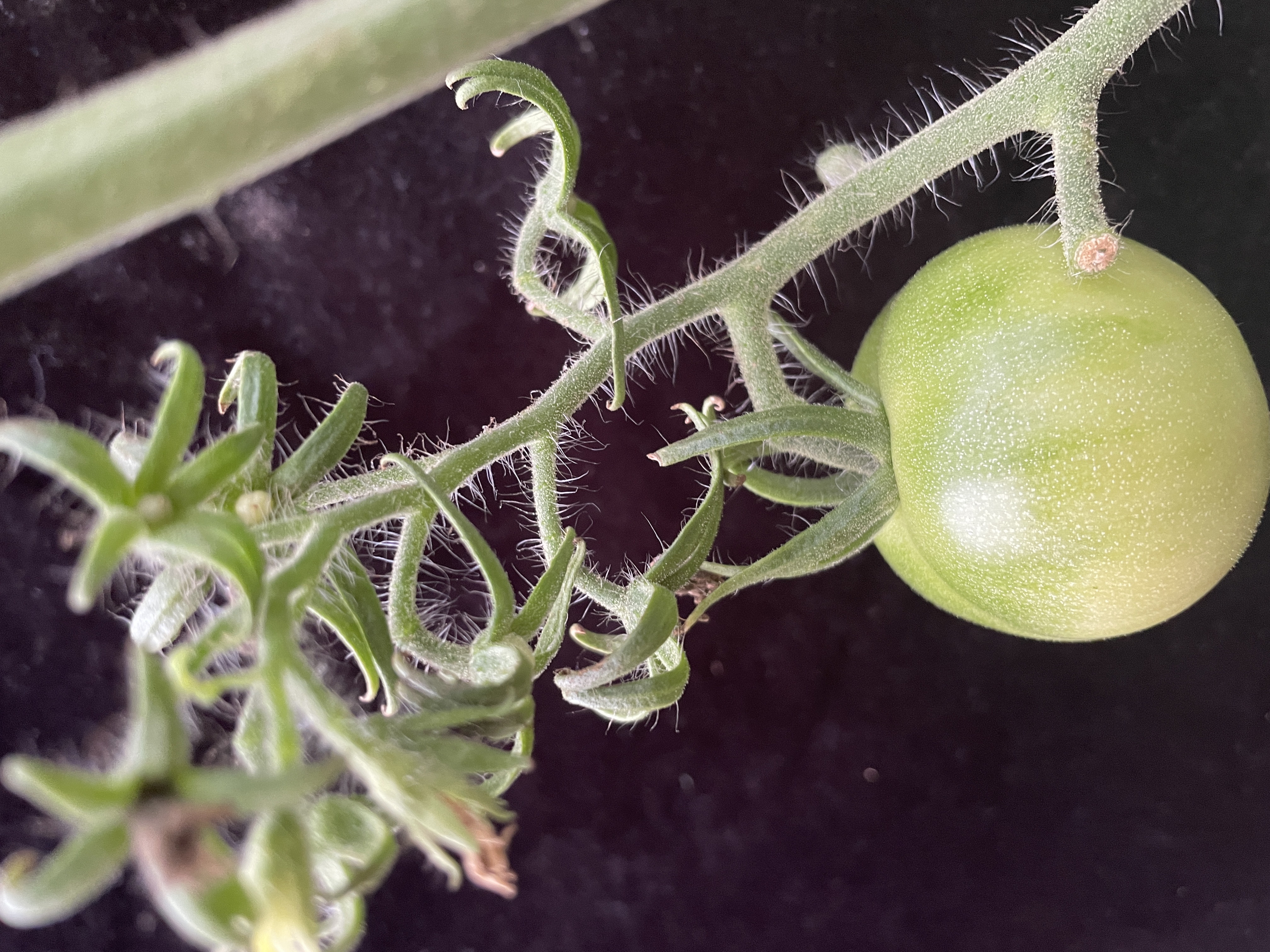

Supplement: Supplementary file 13 — Source data Fig. 1 [file 44318_2026_708_MOESM13_ESM.zip › Source Data Fig 1/Source Data Fig 1A/Control-40dpa.JPG]

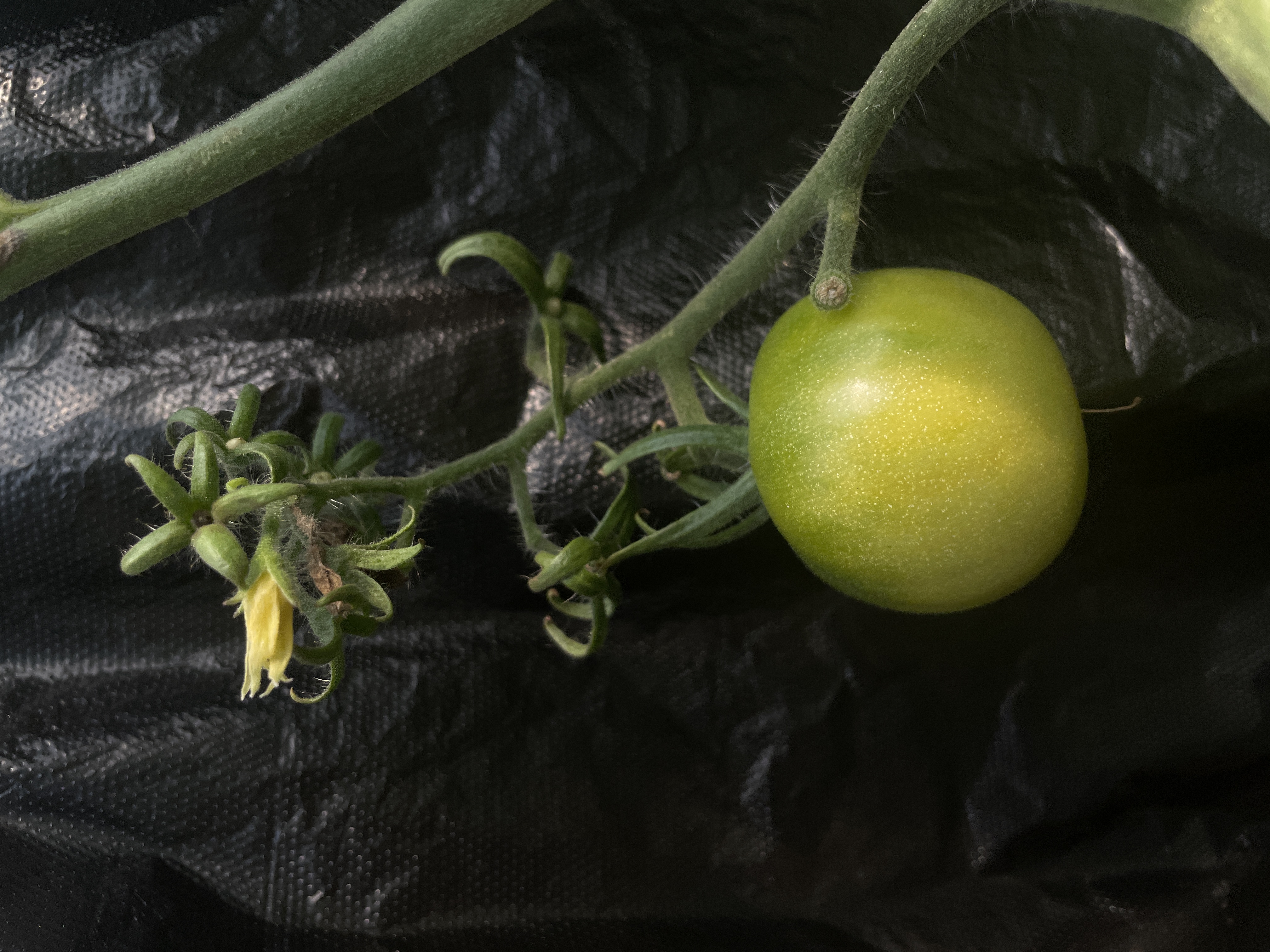

Supplement: Supplementary file 13 — Source data Fig. 1 [file 44318_2026_708_MOESM13_ESM.zip › Source Data Fig 1/Source Data Fig 1A/Control-43dpa.jpg]

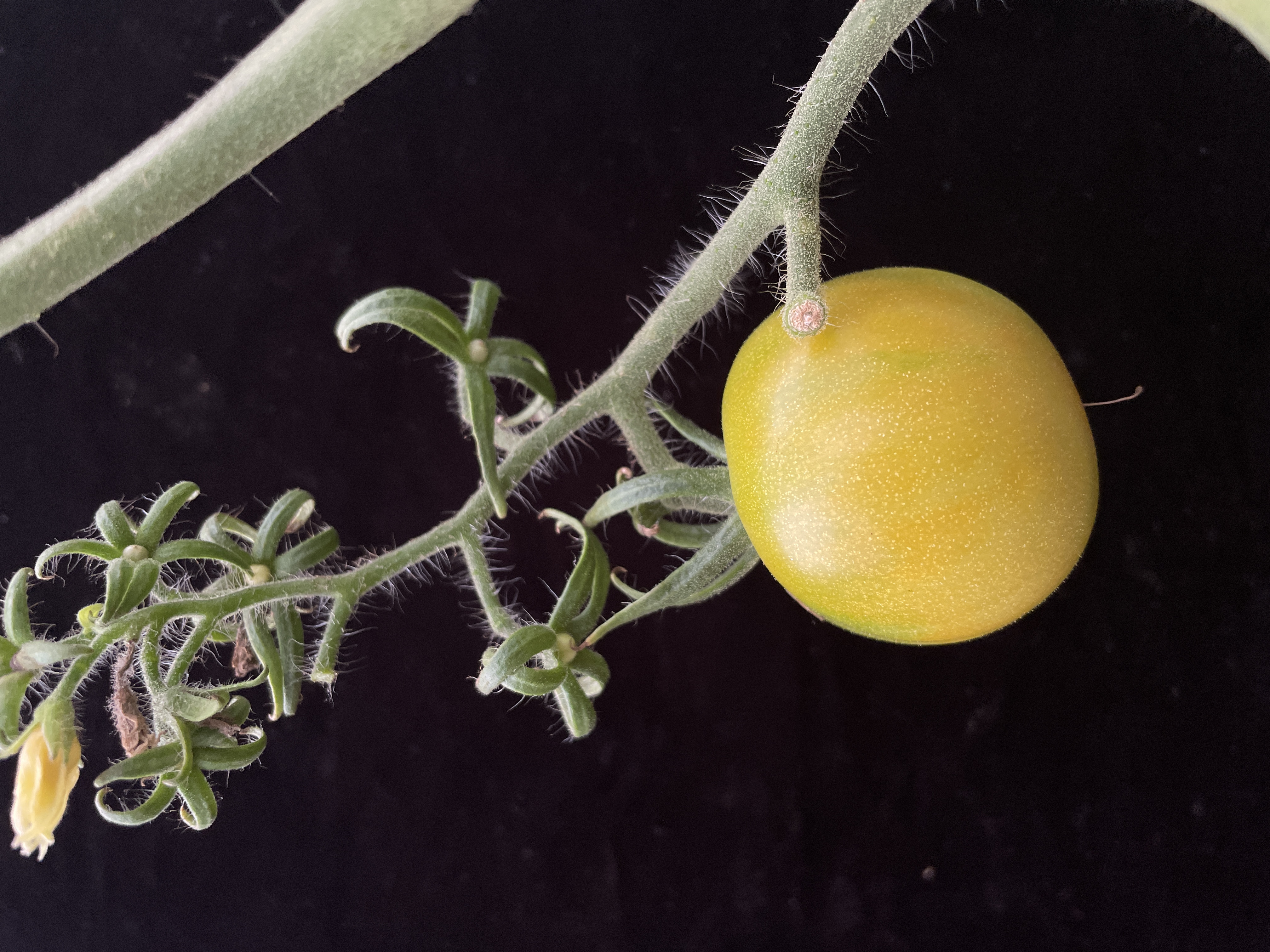

Supplement: Supplementary file 13 — Source data Fig. 1 [file 44318_2026_708_MOESM13_ESM.zip › Source Data Fig 1/Source Data Fig 1A/Control-48dpa.jpg]

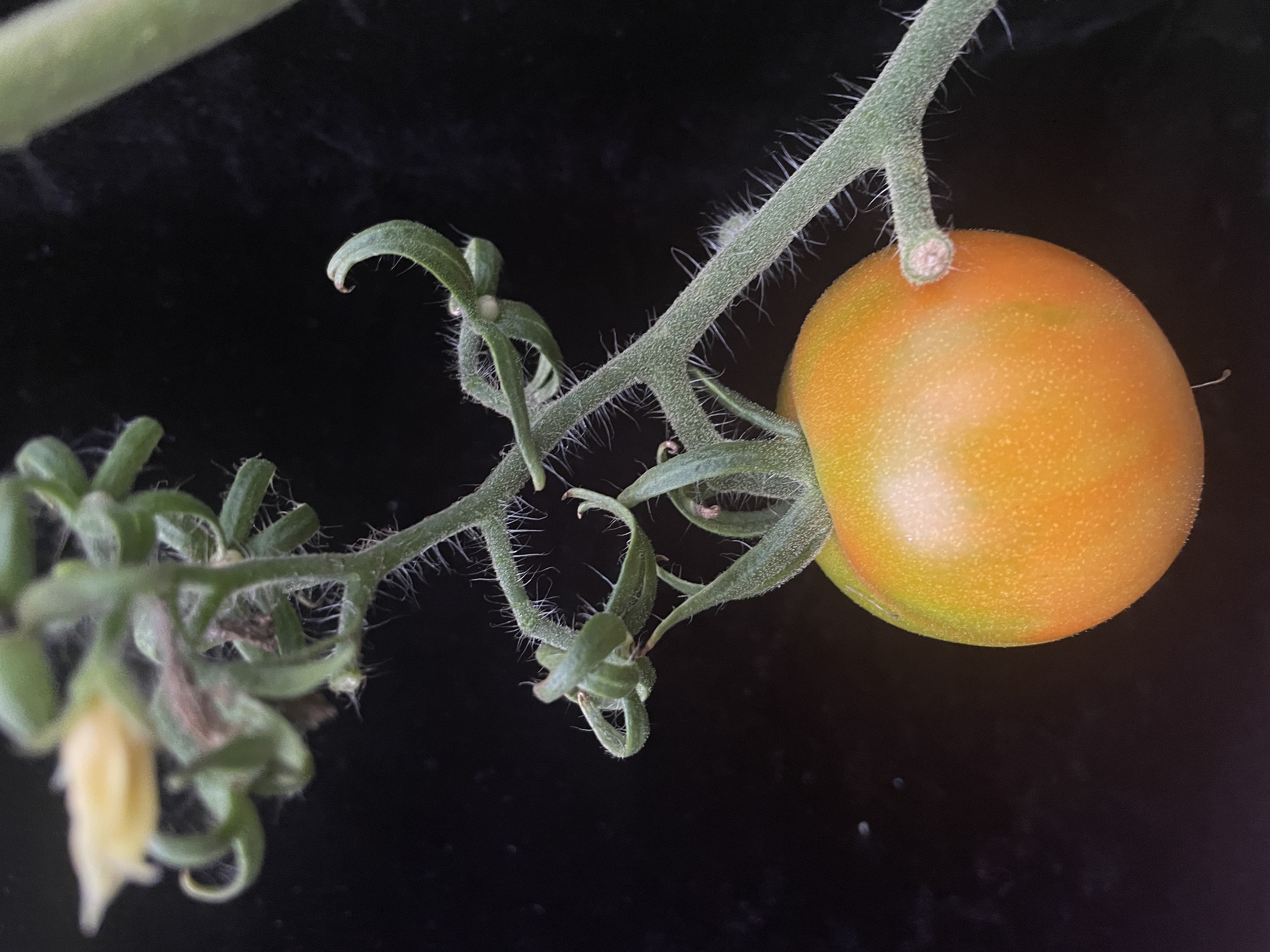

Supplement: Supplementary file 13 — Source data Fig. 1 [file 44318_2026_708_MOESM13_ESM.zip › Source Data Fig 1/Source Data Fig 1A/Control-51dpa.jpg]

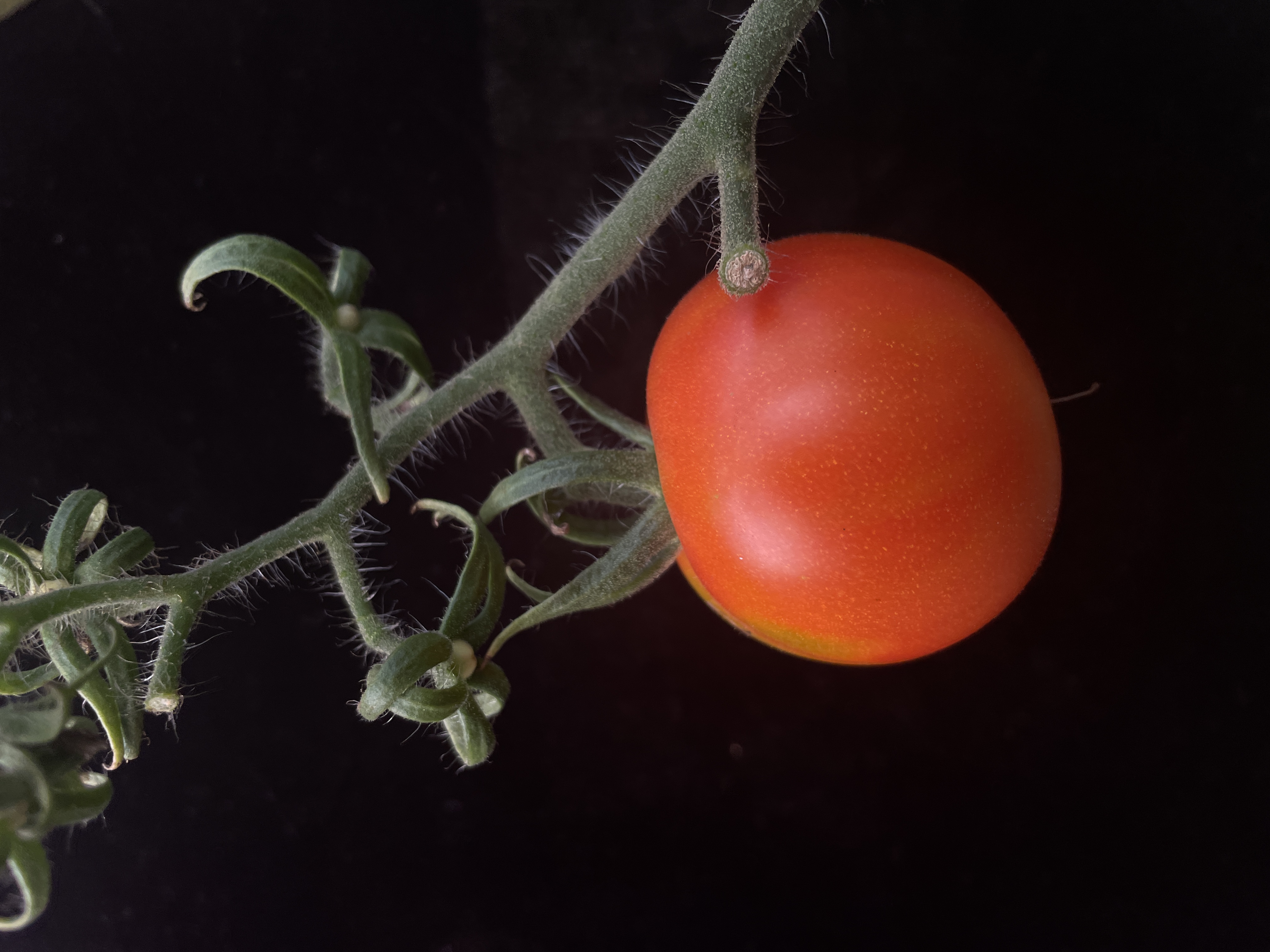

Supplement: Supplementary file 13 — Source data Fig. 1 [file 44318_2026_708_MOESM13_ESM.zip › Source Data Fig 1/Source Data Fig 1A/Control-55dpa.jpg]

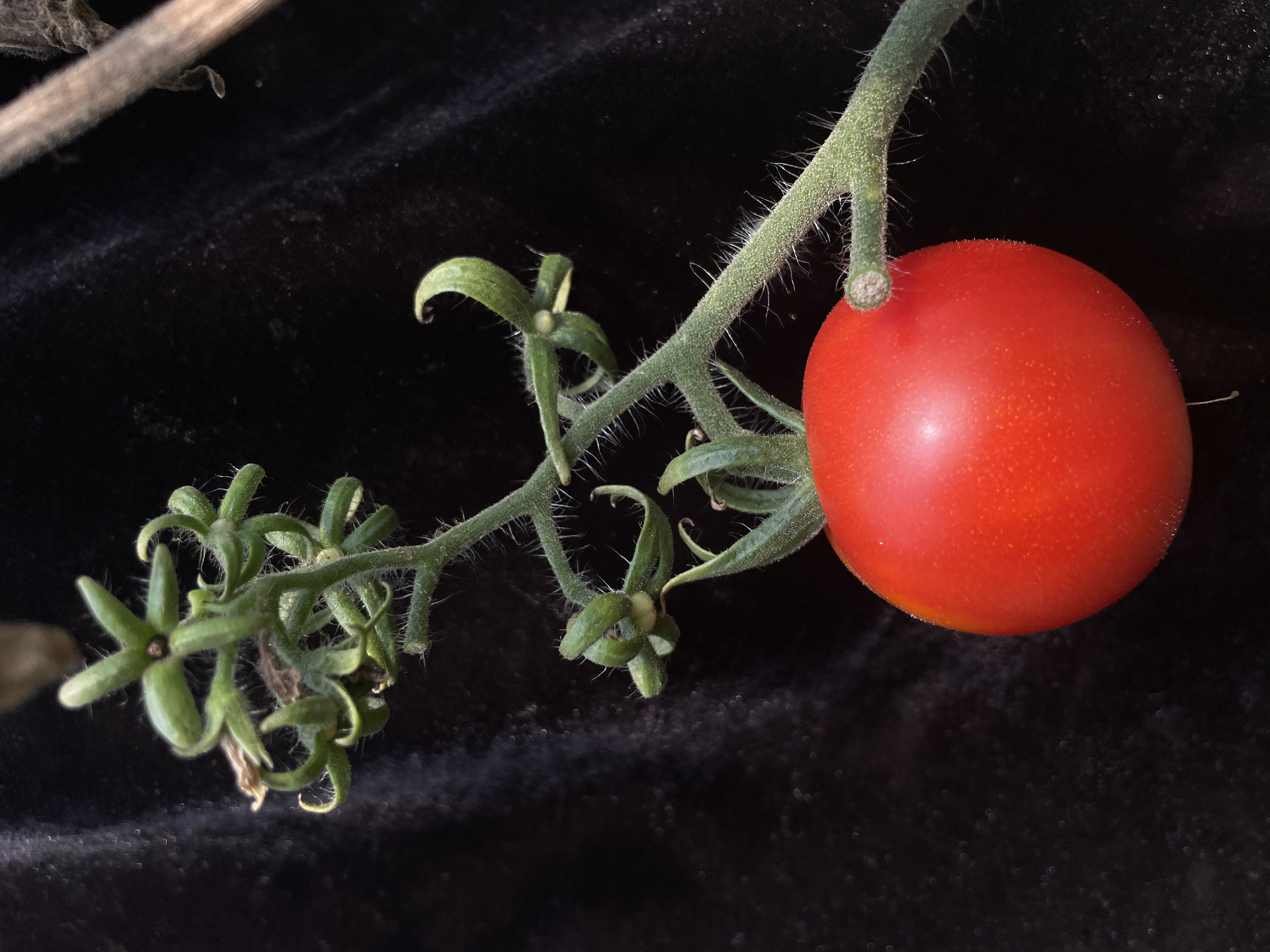

Supplement: Supplementary file 13 — Source data Fig. 1 [file 44318_2026_708_MOESM13_ESM.zip › Source Data Fig 1/Source Data Fig 1A/Control-60dpa.jpg]

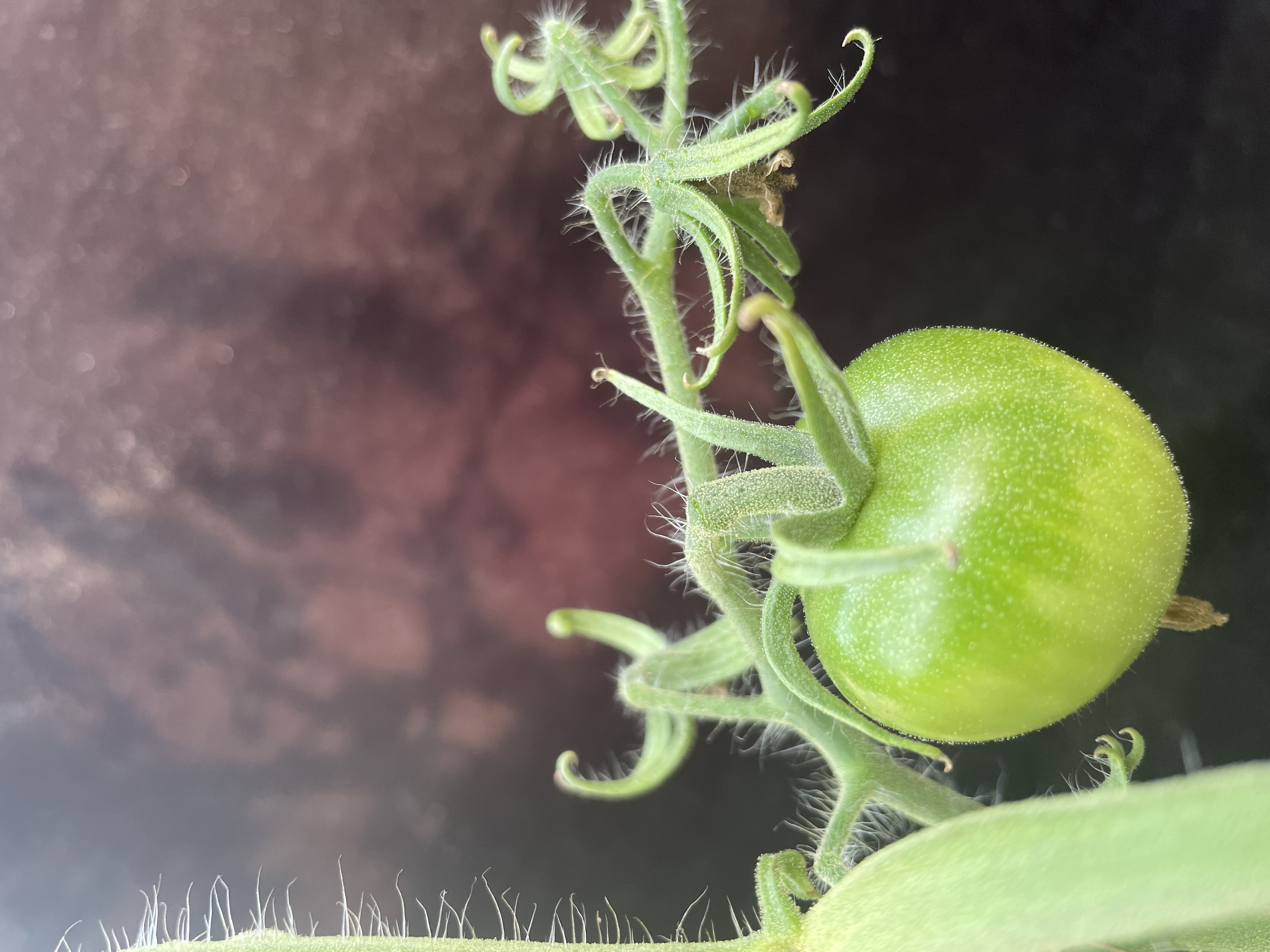

Supplement: Supplementary file 13 — Source data Fig. 1 [file 44318_2026_708_MOESM13_ESM.zip › Source Data Fig 1/Source Data Fig 1A/salt-32dpa.jpg]

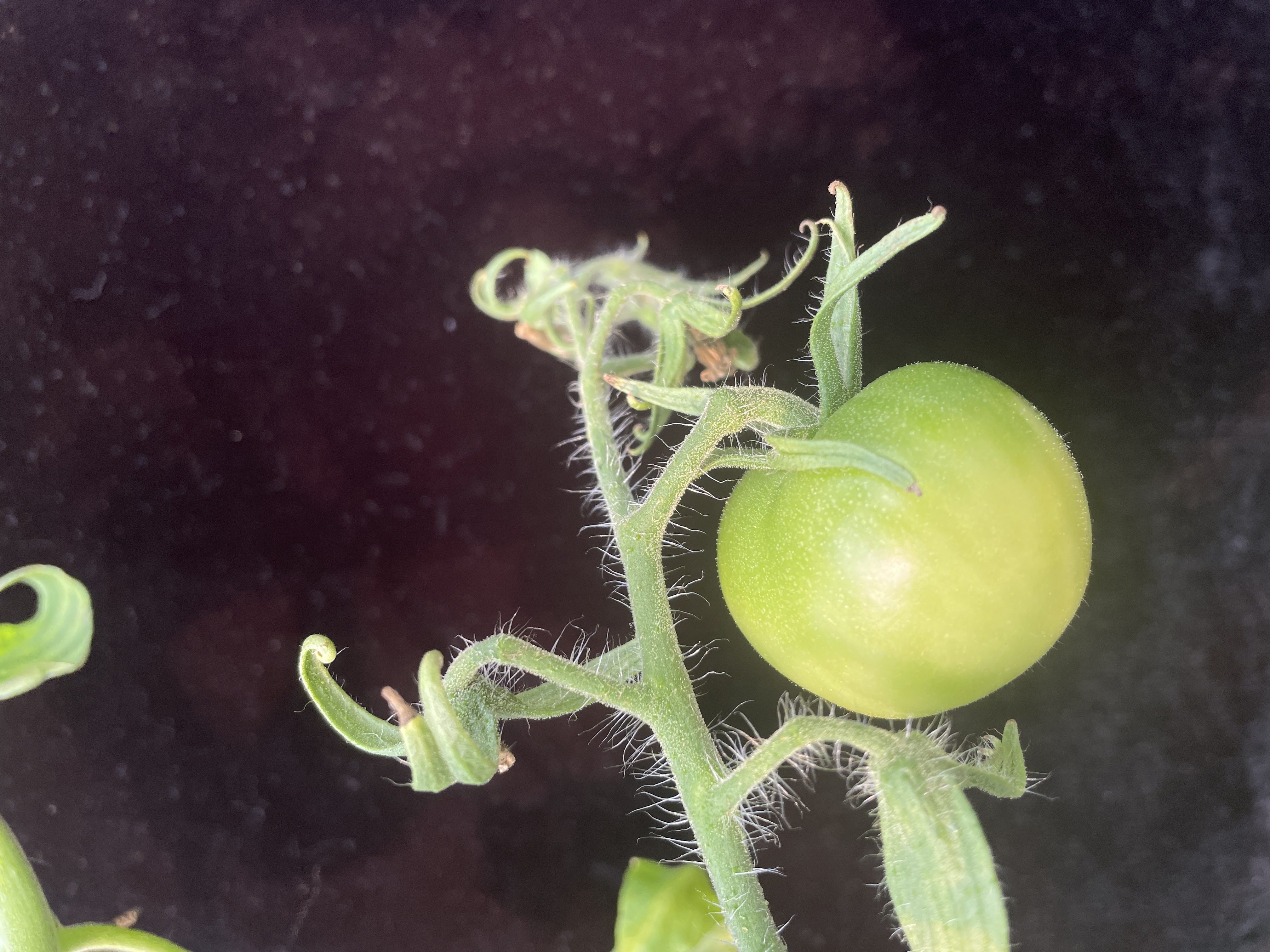

Supplement: Supplementary file 13 — Source data Fig. 1 [file 44318_2026_708_MOESM13_ESM.zip › Source Data Fig 1/Source Data Fig 1A/salt-36dpa.JPG]

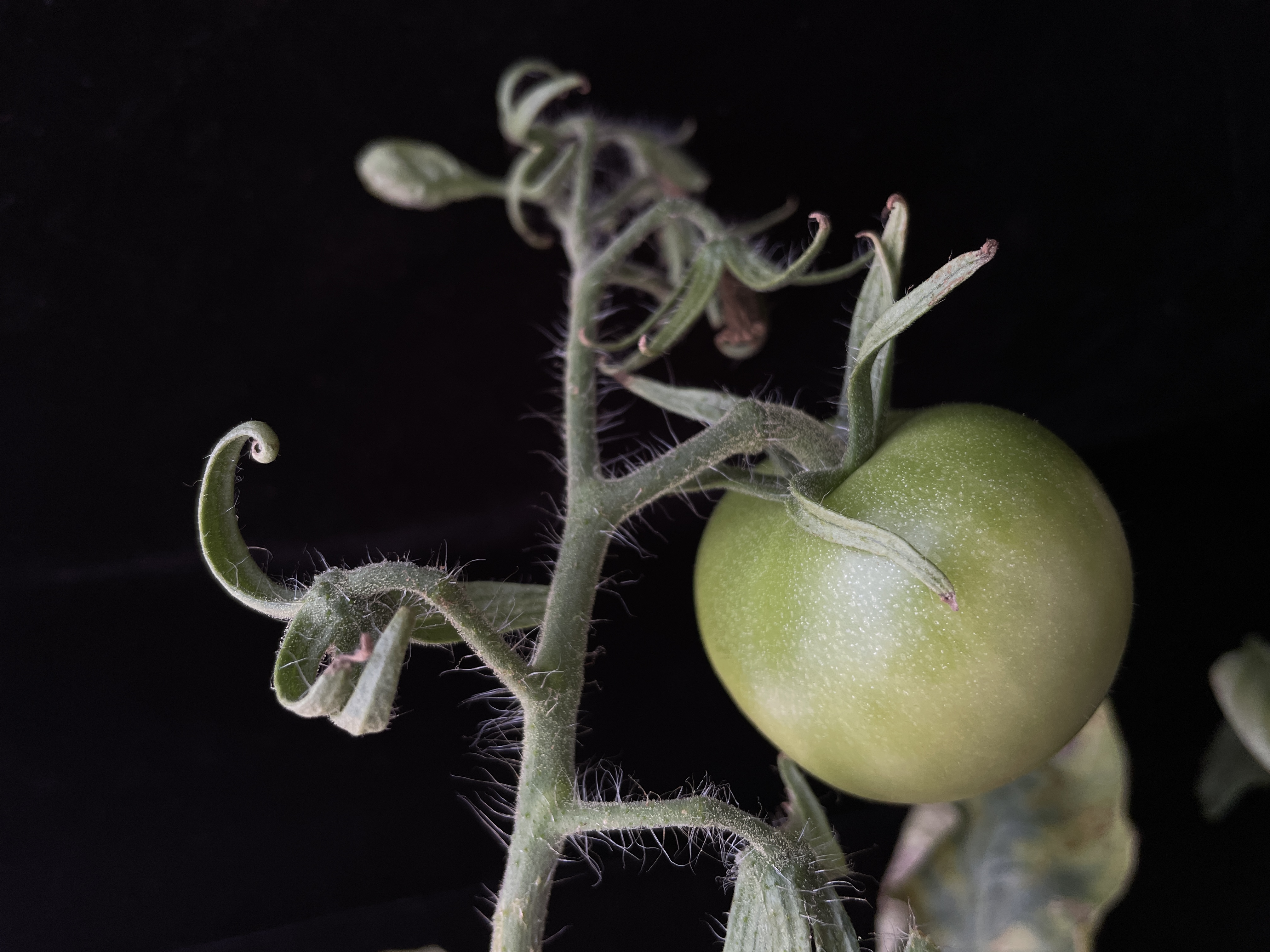

Supplement: Supplementary file 13 — Source data Fig. 1 [file 44318_2026_708_MOESM13_ESM.zip › Source Data Fig 1/Source Data Fig 1A/salt-40dpa.JPG]

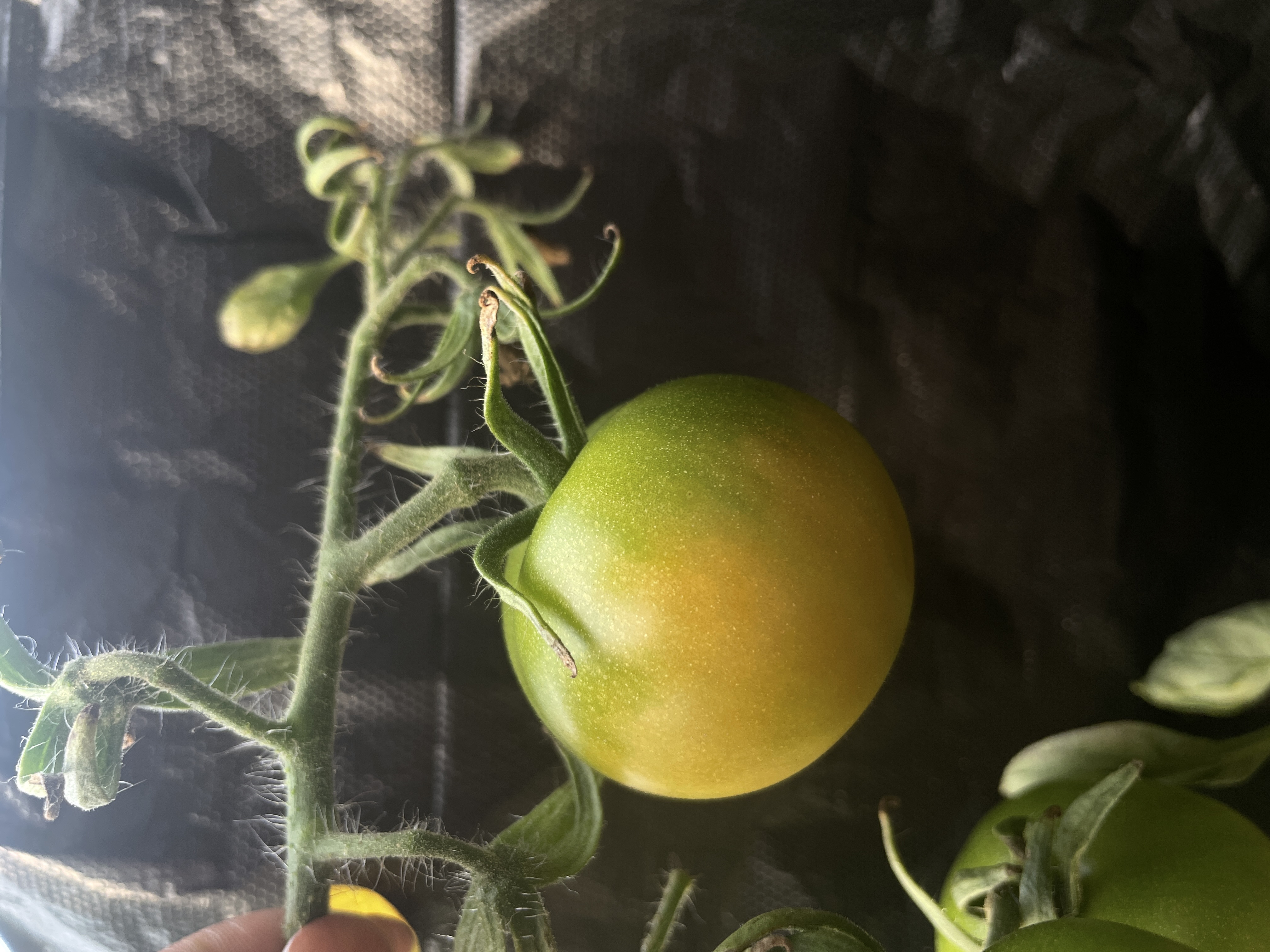

Supplement: Supplementary file 13 — Source data Fig. 1 [file 44318_2026_708_MOESM13_ESM.zip › Source Data Fig 1/Source Data Fig 1A/salt-43dpa.jpg]

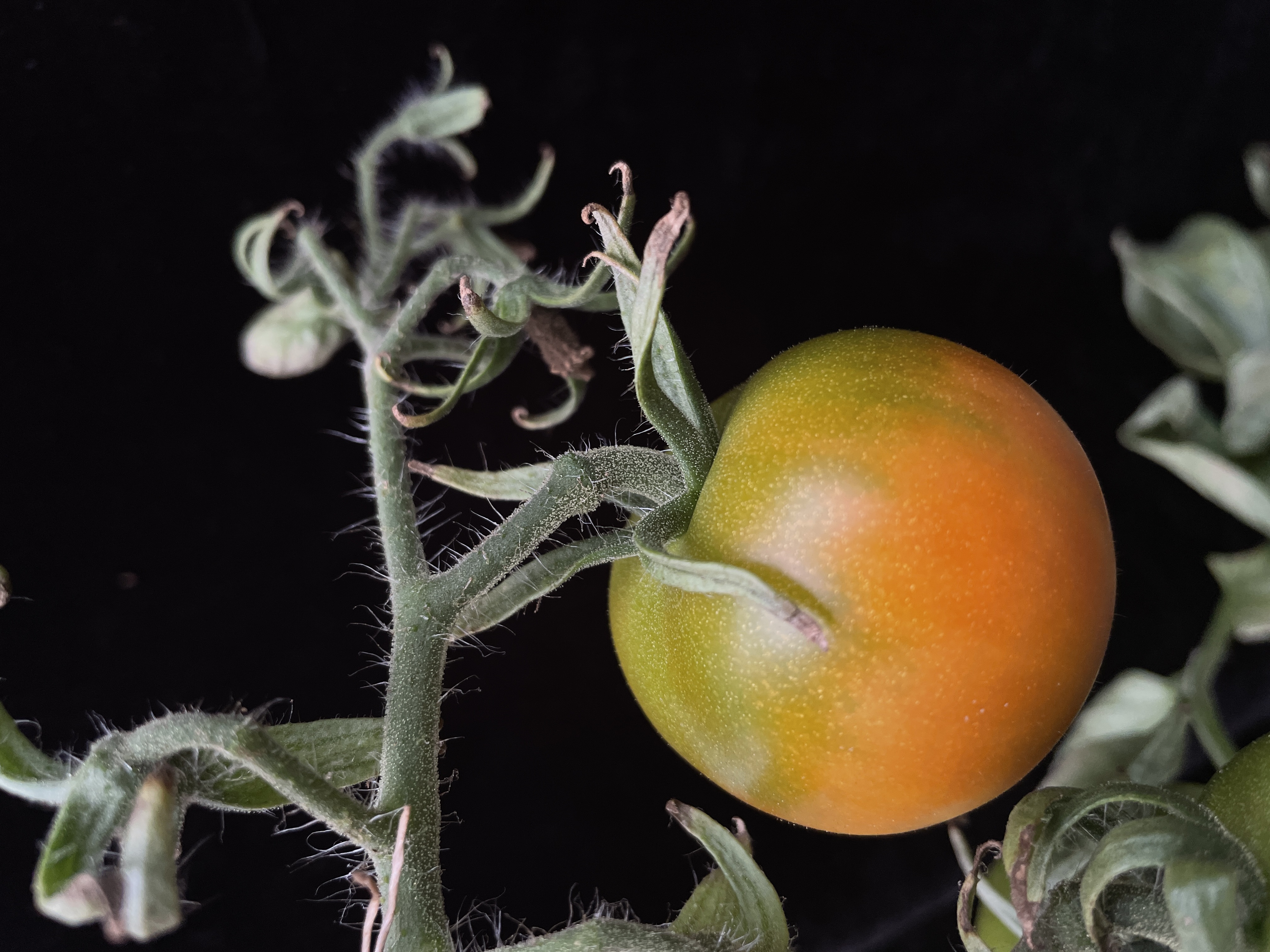

Supplement: Supplementary file 13 — Source data Fig. 1 [file 44318_2026_708_MOESM13_ESM.zip › Source Data Fig 1/Source Data Fig 1A/salt-48dpa.jpg]

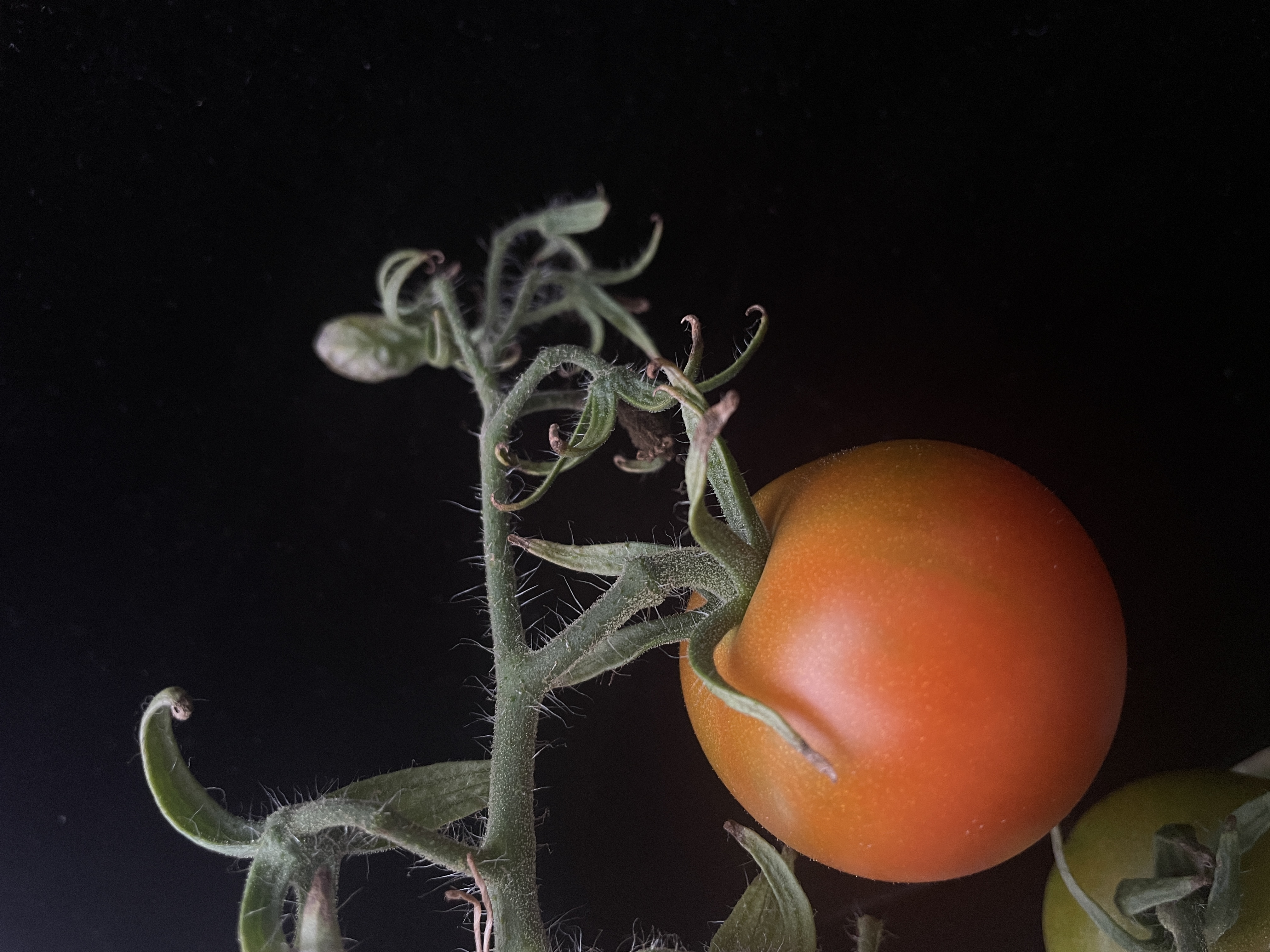

Supplement: Supplementary file 13 — Source data Fig. 1 [file 44318_2026_708_MOESM13_ESM.zip › Source Data Fig 1/Source Data Fig 1A/salt-51dpa.jpg]

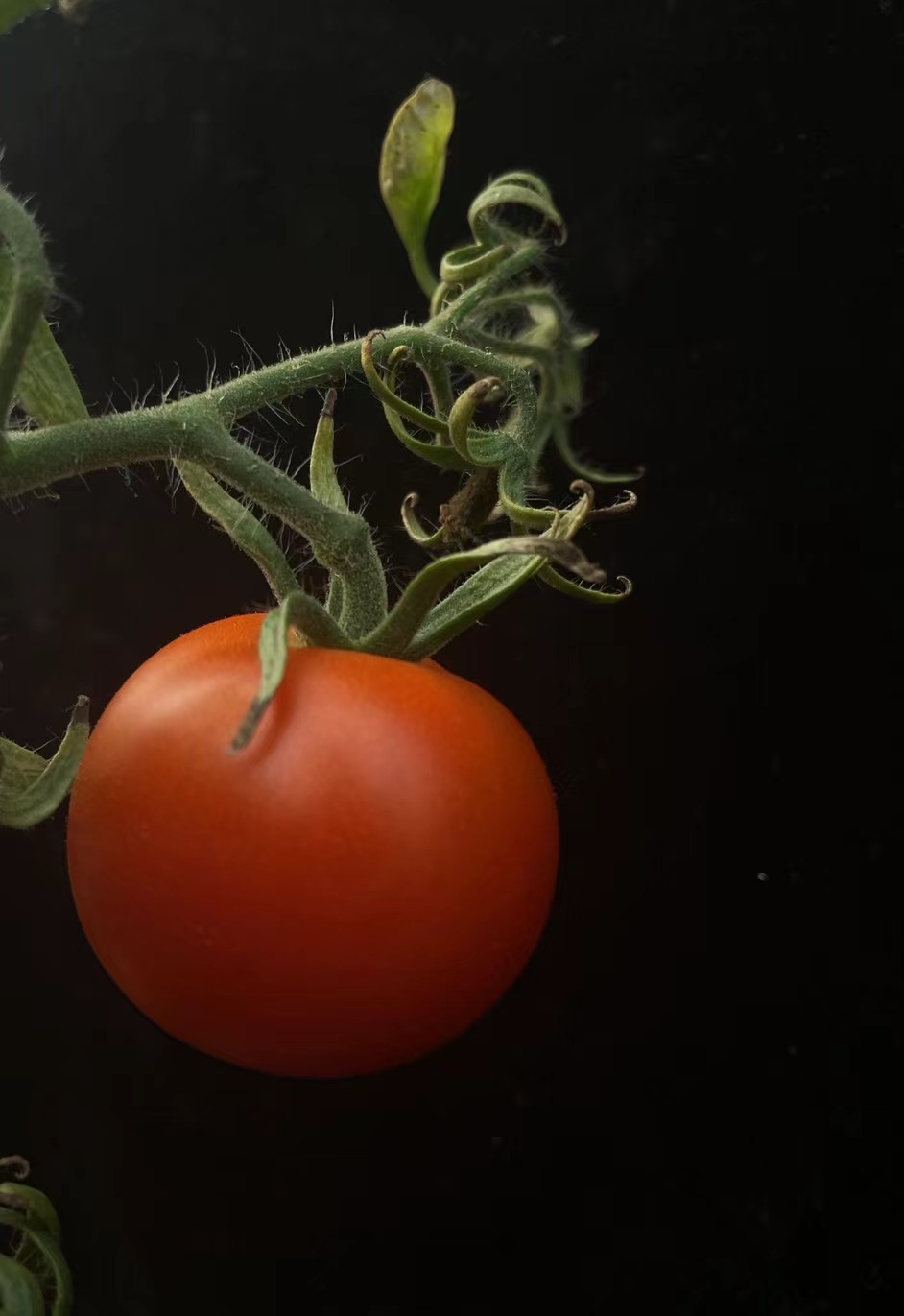

Supplement: Supplementary file 13 — Source data Fig. 1 [file 44318_2026_708_MOESM13_ESM.zip › Source Data Fig 1/Source Data Fig 1A/salt-55dpa.jpg]

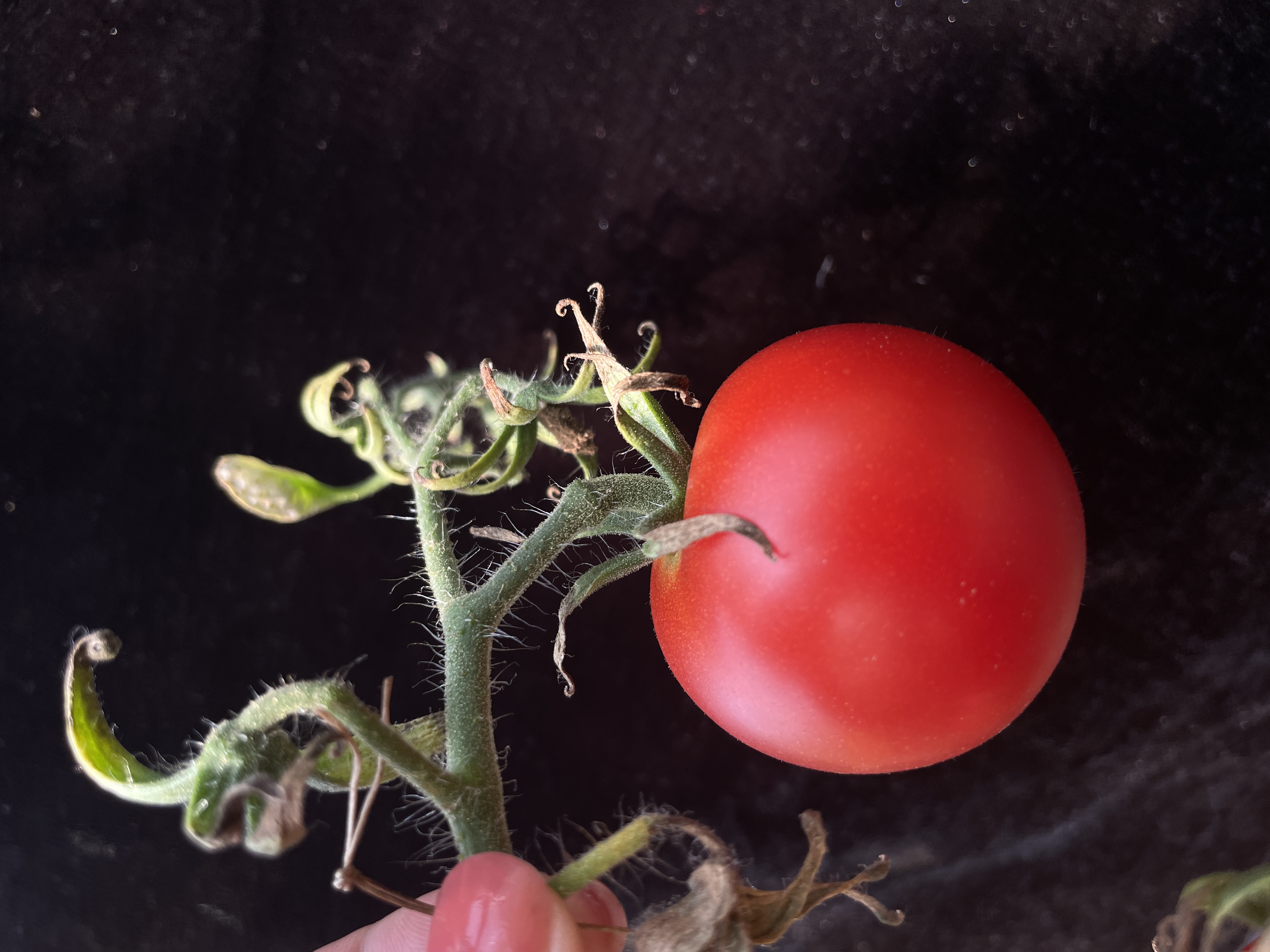

Supplement: Supplementary file 13 — Source data Fig. 1 [file 44318_2026_708_MOESM13_ESM.zip › Source Data Fig 1/Source Data Fig 1A/salt-60dpa.jpg]

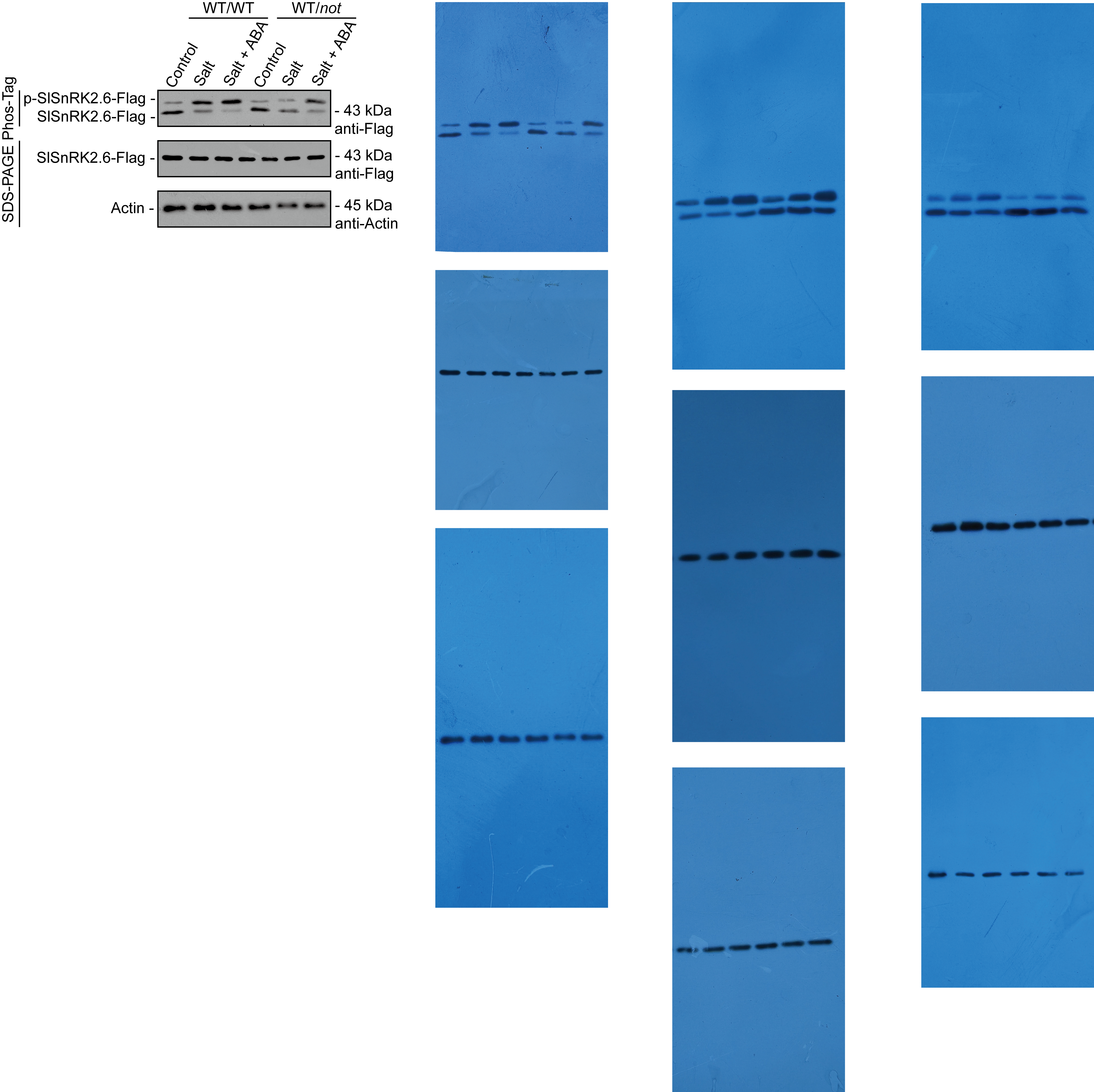

Supplement: Supplementary file 14 — Source data Fig. 2 [file 44318_2026_708_MOESM14_ESM.zip › Source Data Fig 2/Source Data Fig 2F/Fig 2F.tif]

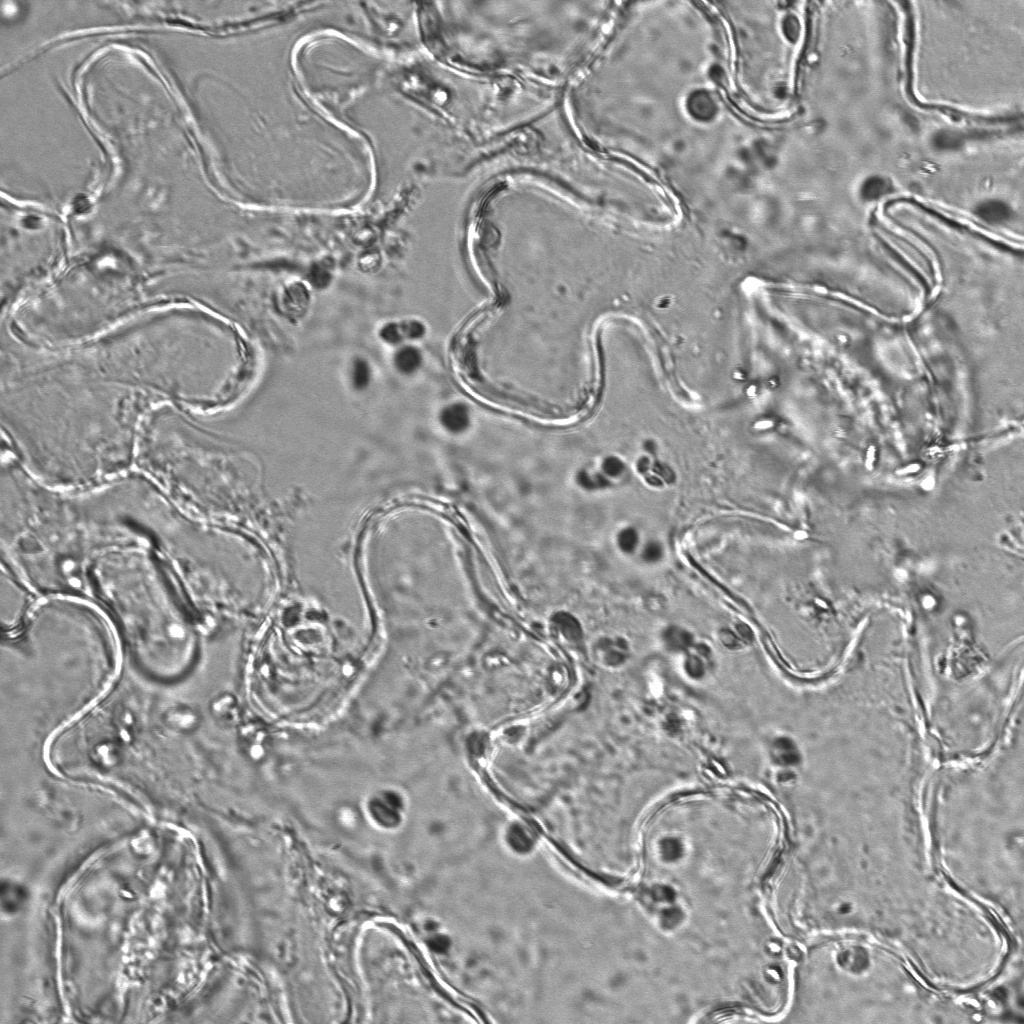

Supplement: Supplementary file 15 — Source data Fig. 3 [file 44318_2026_708_MOESM15_ESM.zip › Source Data Fig 3/Source Data Fig 3A/Bright.jpg]

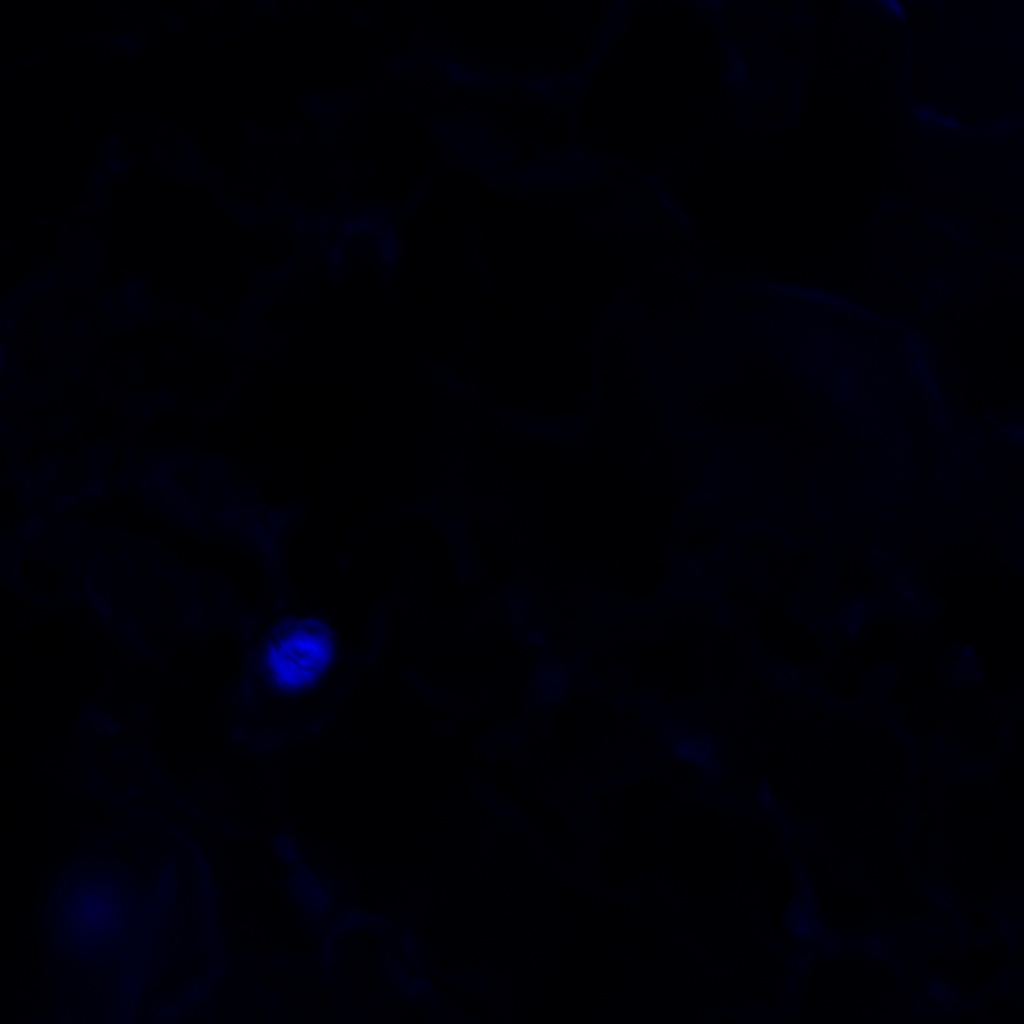

Supplement: Supplementary file 15 — Source data Fig. 3 [file 44318_2026_708_MOESM15_ESM.zip › Source Data Fig 3/Source Data Fig 3A/DAPI.jpg]

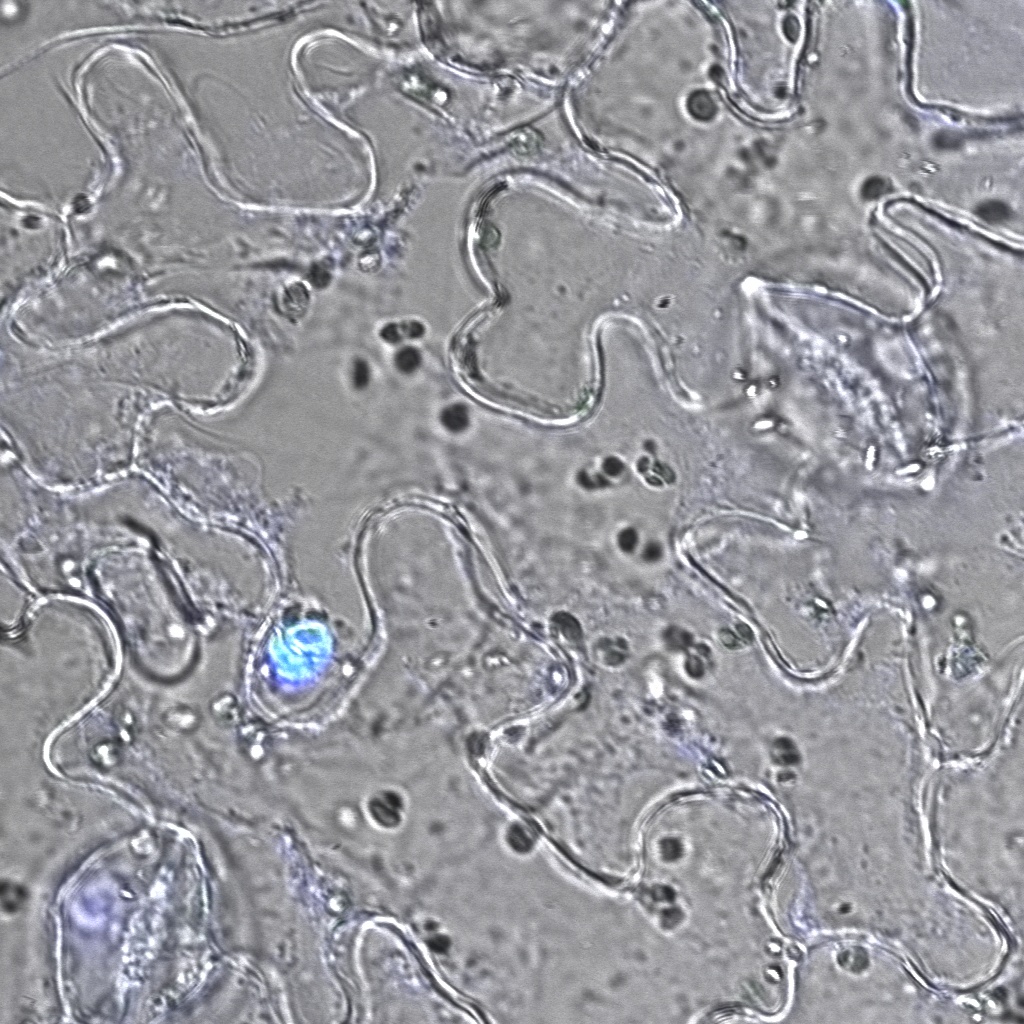

Supplement: Supplementary file 15 — Source data Fig. 3 [file 44318_2026_708_MOESM15_ESM.zip › Source Data Fig 3/Source Data Fig 3A/Merge.jpg]

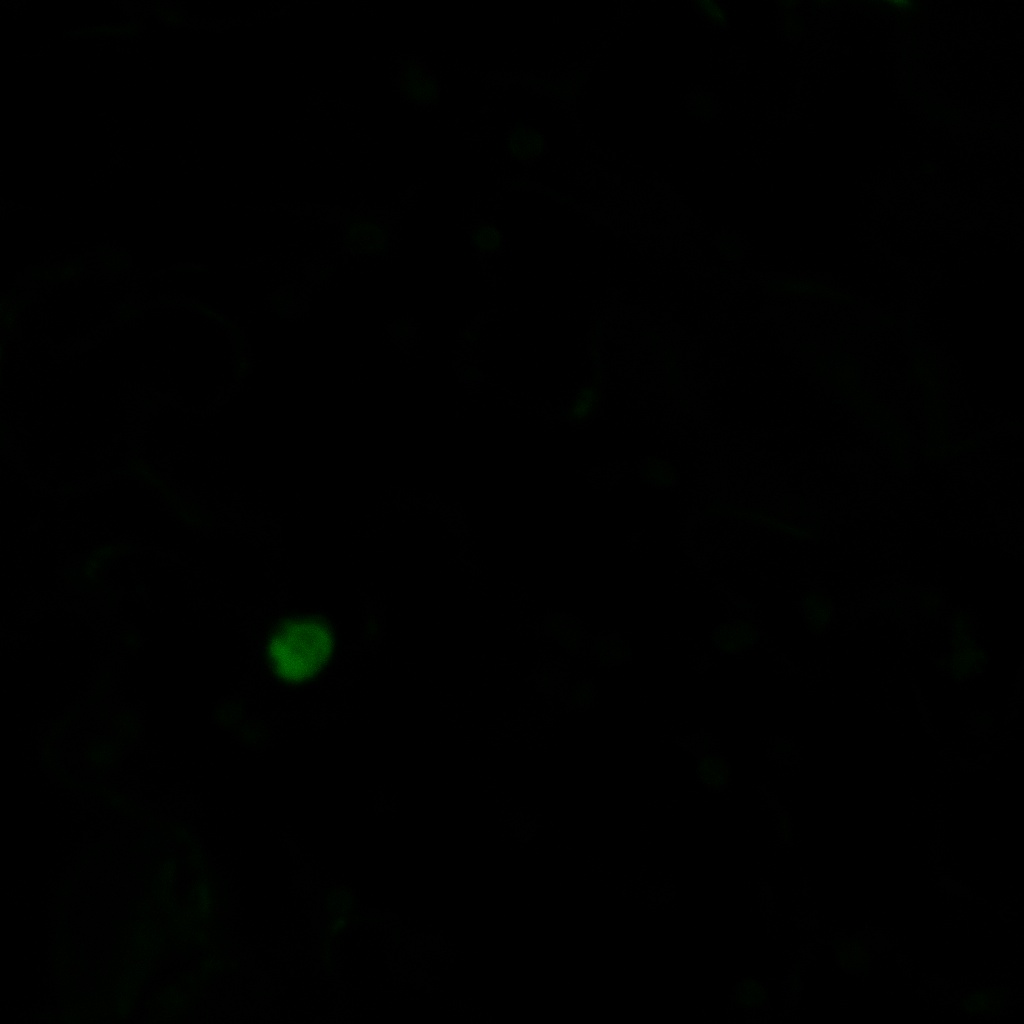

Supplement: Supplementary file 15 — Source data Fig. 3 [file 44318_2026_708_MOESM15_ESM.zip › Source Data Fig 3/Source Data Fig 3A/SlZHD8-GFP.jpg]

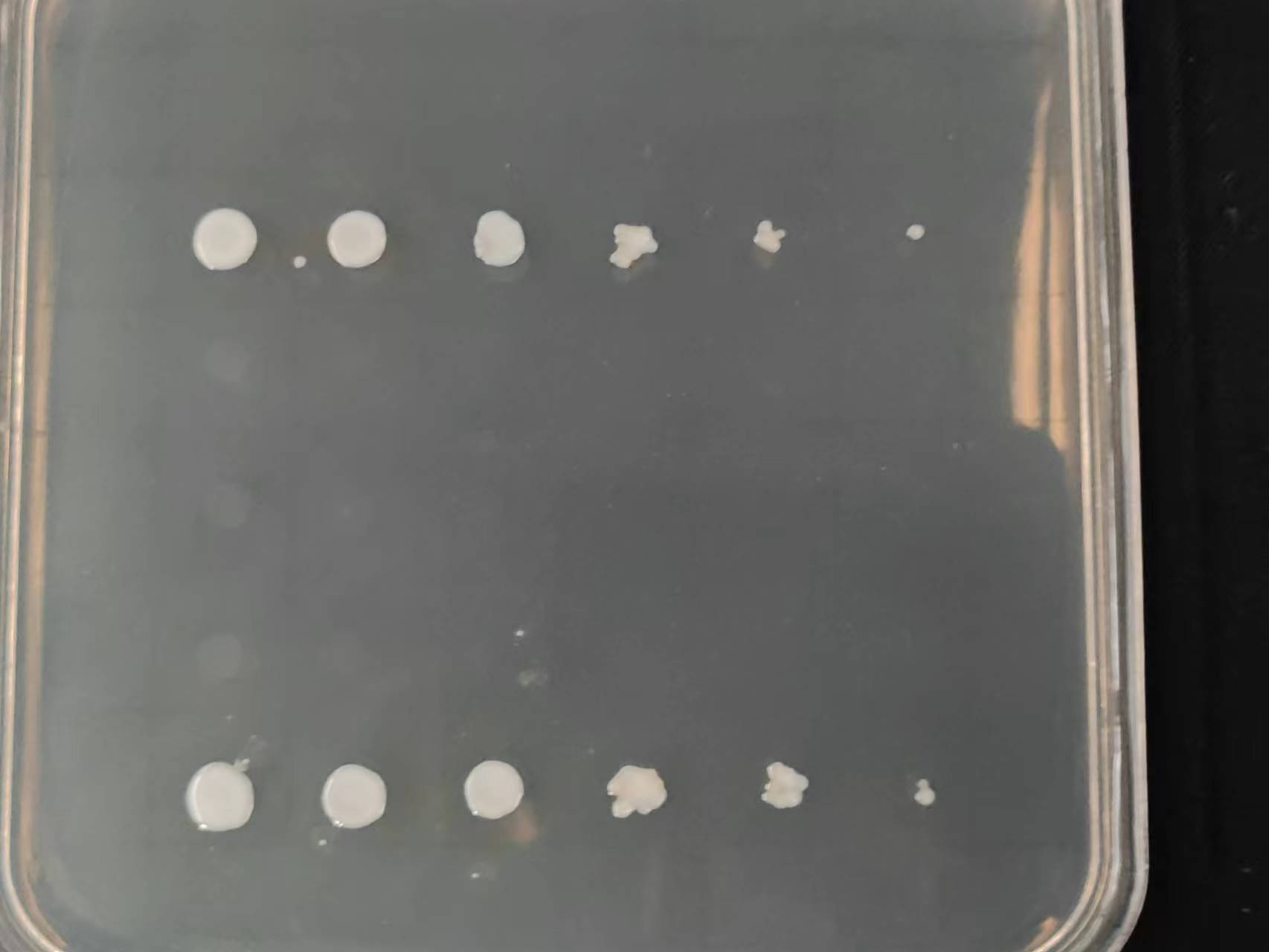

Supplement: Supplementary file 15 — Source data Fig. 3 [file 44318_2026_708_MOESM15_ESM.zip › Source Data Fig 3/Source Data Fig 3B/-Leu-Trp-His-Ade.jpg]

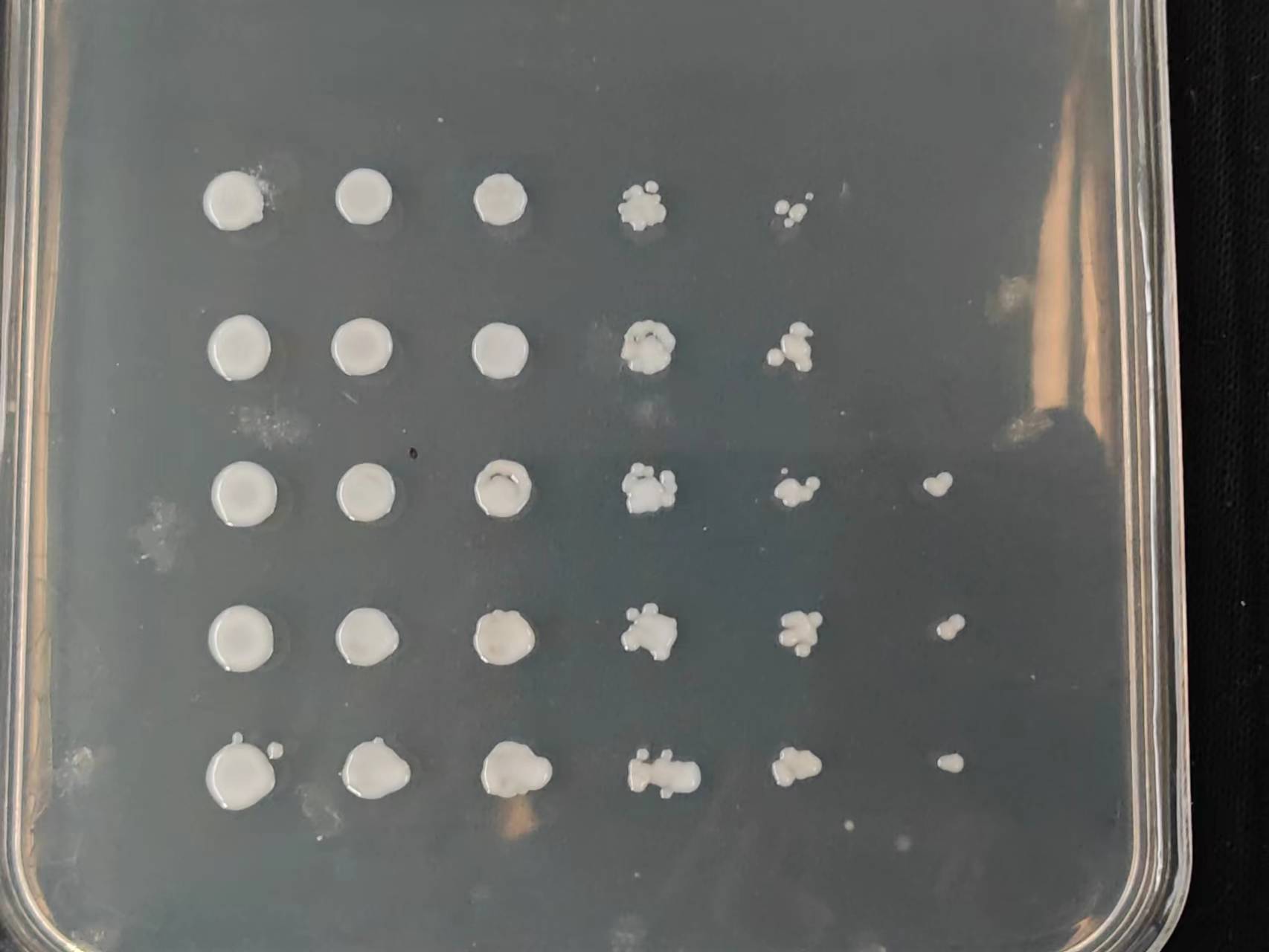

Supplement: Supplementary file 15 — Source data Fig. 3 [file 44318_2026_708_MOESM15_ESM.zip › Source Data Fig 3/Source Data Fig 3B/-Leu-Trp.jpg]

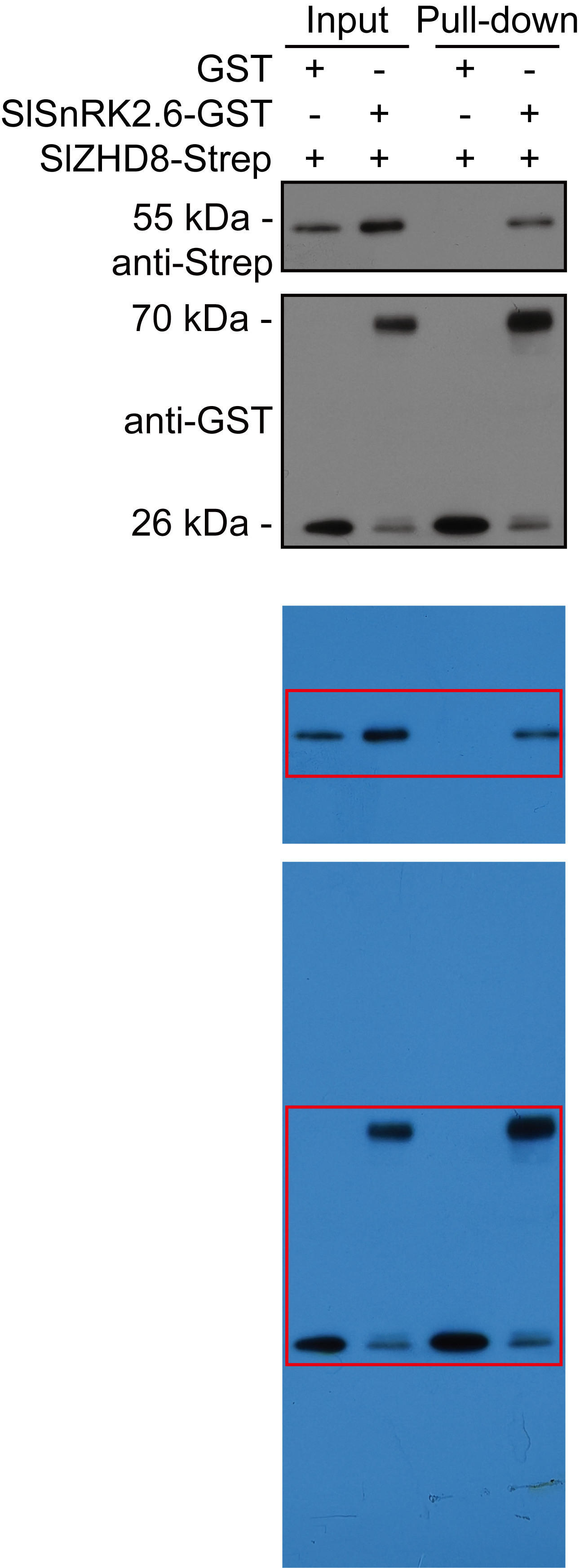

Supplement: Supplementary file 15 — Source data Fig. 3 [file 44318_2026_708_MOESM15_ESM.zip › Source Data Fig 3/Source Data Fig 3C/Source Data Fig 3C.tif]

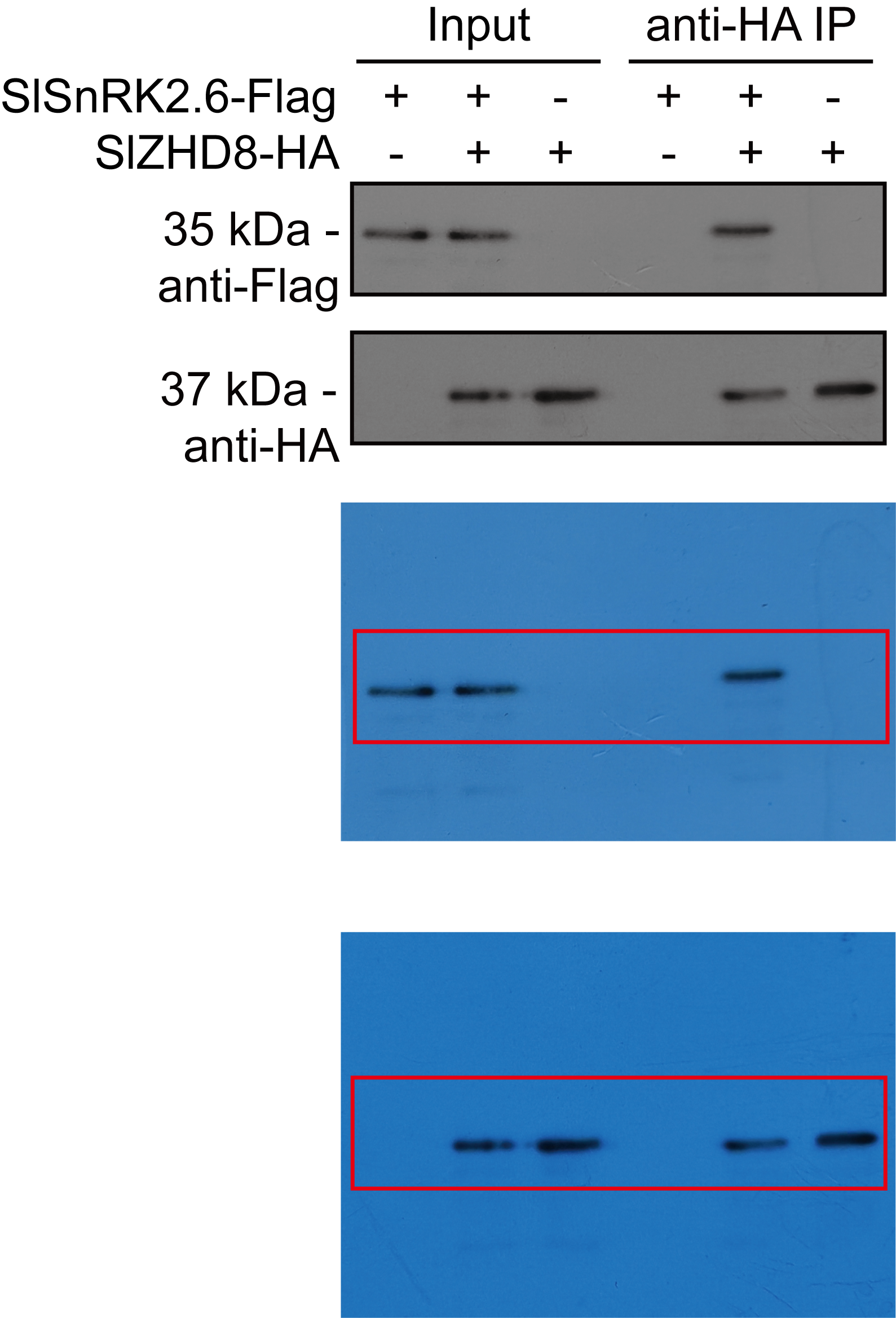

Supplement: Supplementary file 15 — Source data Fig. 3 [file 44318_2026_708_MOESM15_ESM.zip › Source Data Fig 3/Source Data Fig 3D/Source Data Fig 3D.tif]

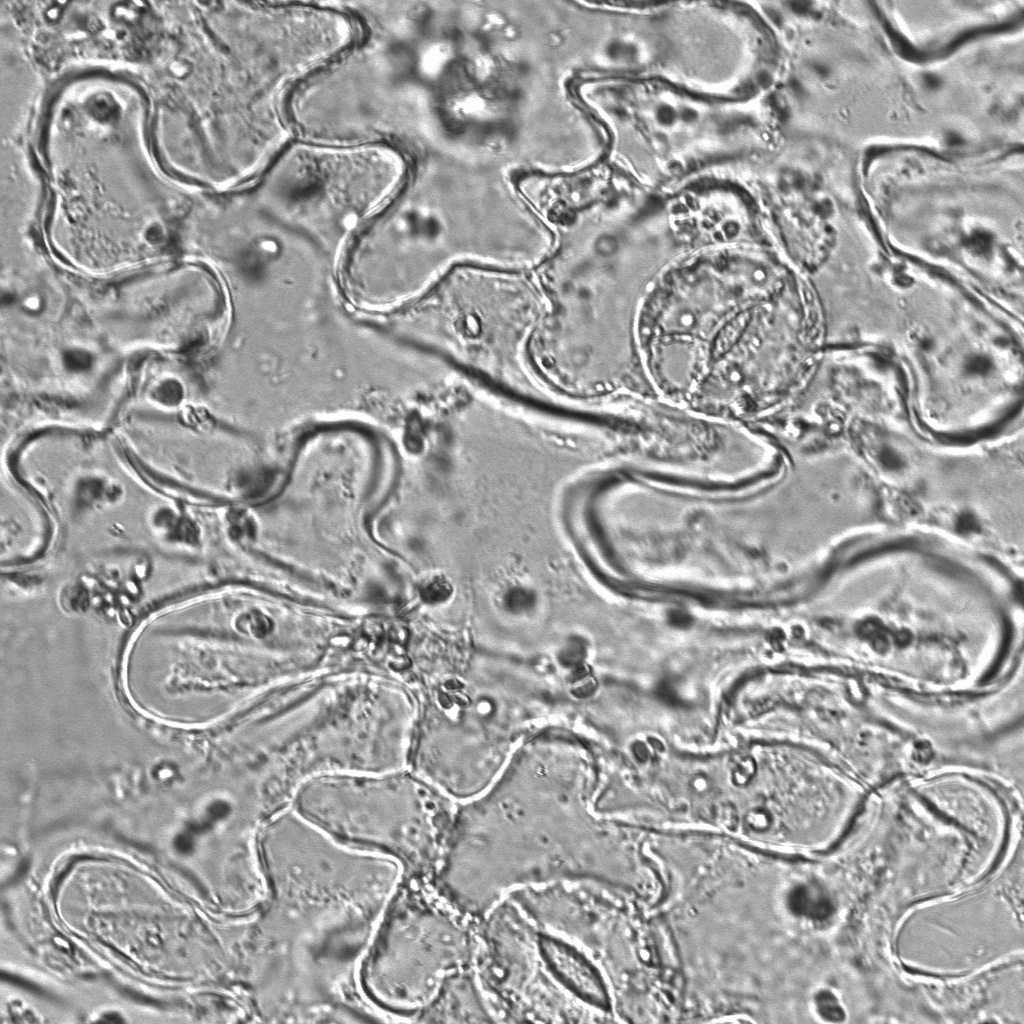

Supplement: Supplementary file 15 — Source data Fig. 3 [file 44318_2026_708_MOESM15_ESM.zip › Source Data Fig 3/Source Data Fig 3E/Birght.jpg]

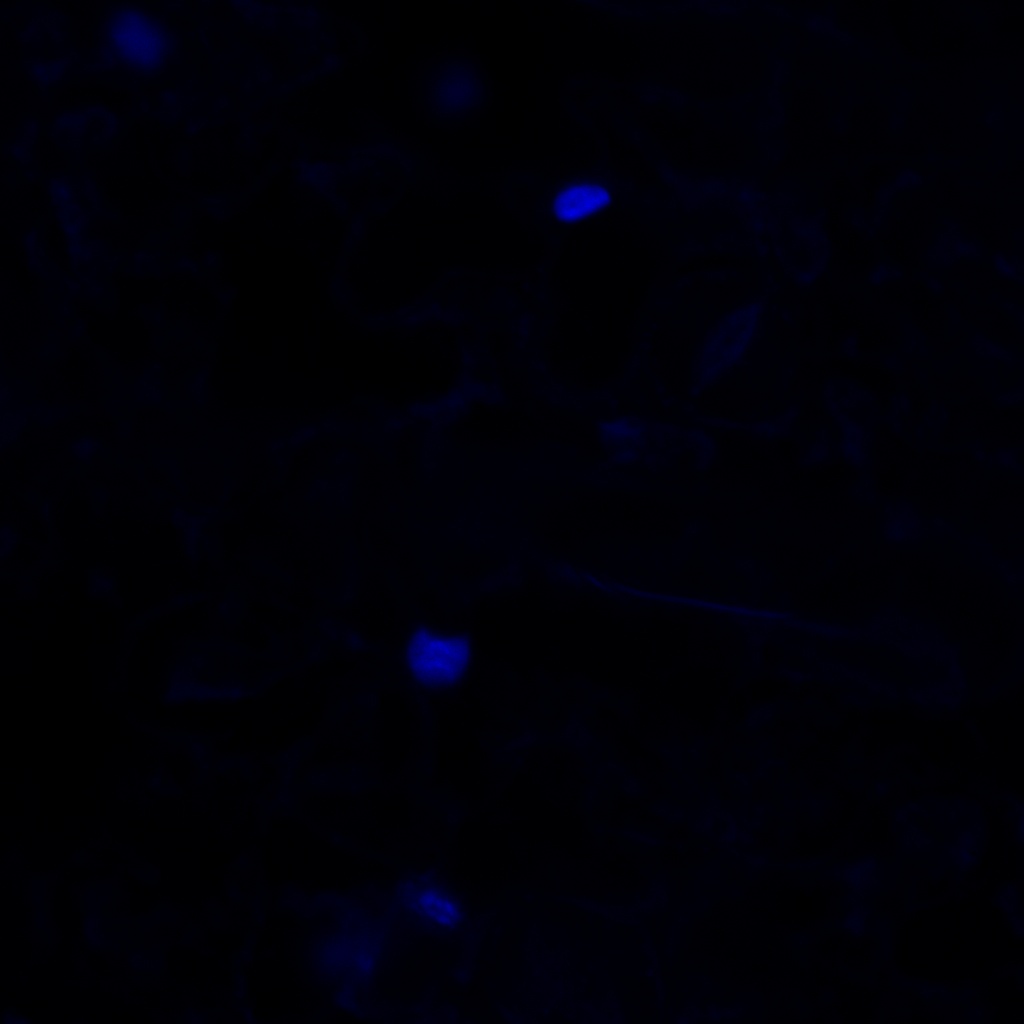

Supplement: Supplementary file 15 — Source data Fig. 3 [file 44318_2026_708_MOESM15_ESM.zip › Source Data Fig 3/Source Data Fig 3E/DAPI.jpg]

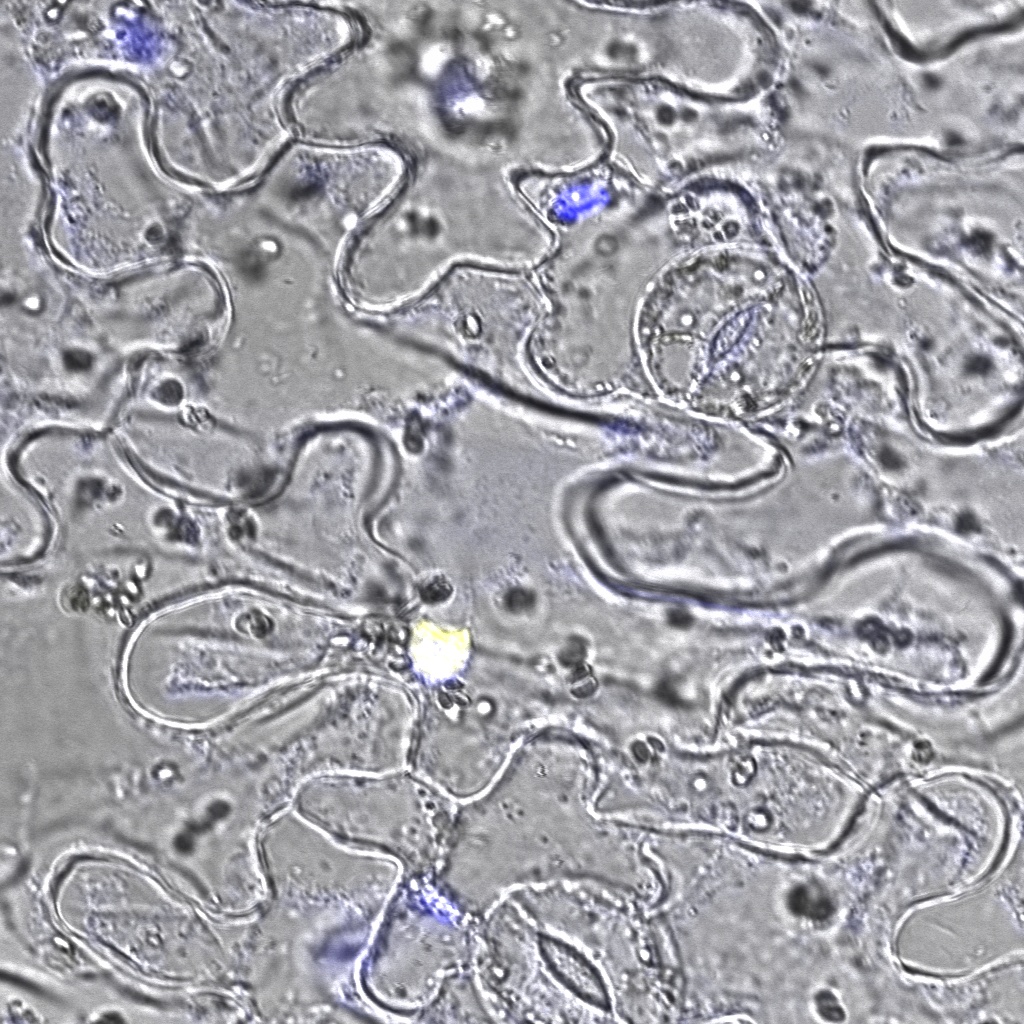

Supplement: Supplementary file 15 — Source data Fig. 3 [file 44318_2026_708_MOESM15_ESM.zip › Source Data Fig 3/Source Data Fig 3E/Merge.jpg]

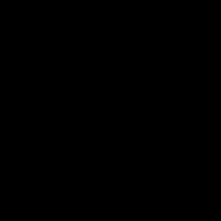

Supplement: Supplementary file 15 — Source data Fig. 3 [file 44318_2026_708_MOESM15_ESM.zip › Source Data Fig 3/Source Data Fig 3E/Negative control 1.jpg]

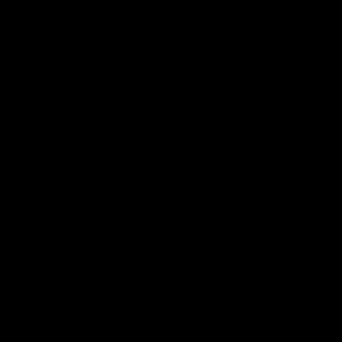

Supplement: Supplementary file 15 — Source data Fig. 3 [file 44318_2026_708_MOESM15_ESM.zip › Source Data Fig 3/Source Data Fig 3E/Negative control 2.jpg]

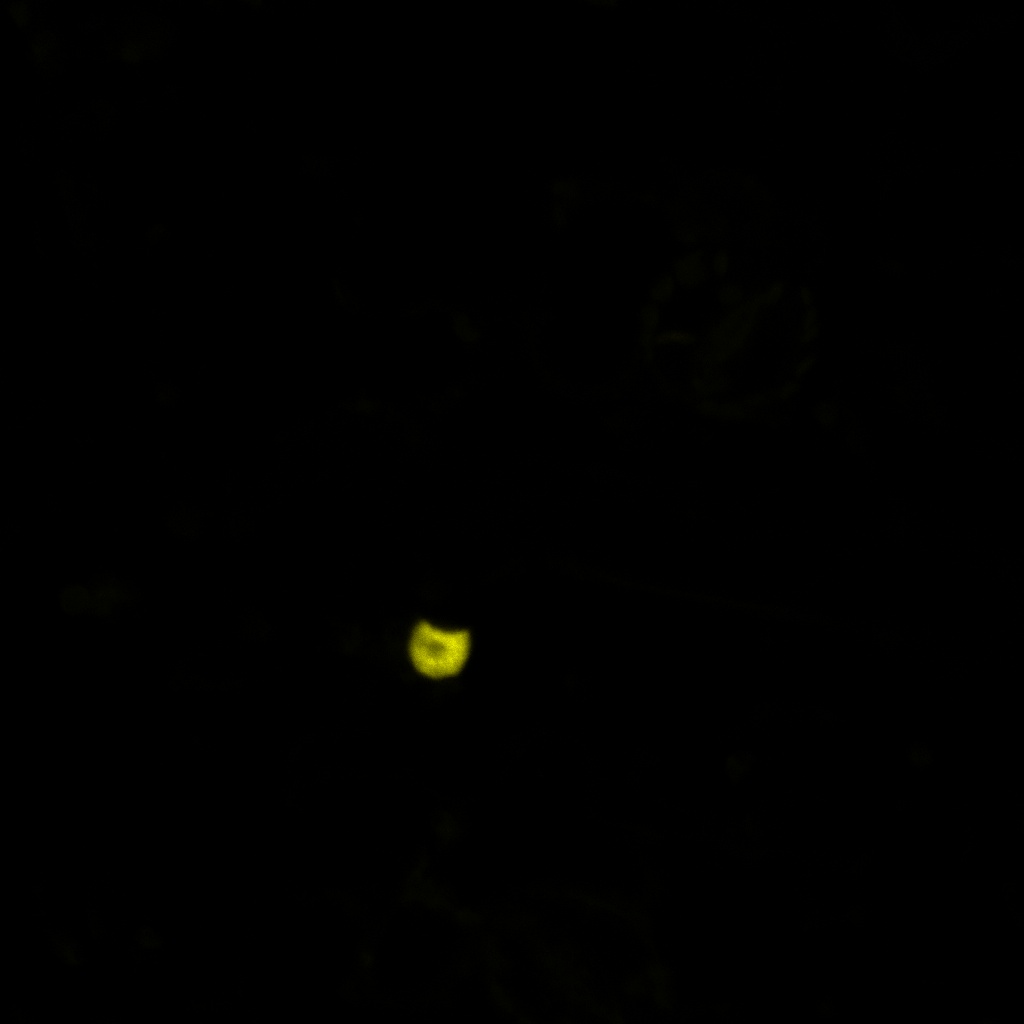

Supplement: Supplementary file 15 — Source data Fig. 3 [file 44318_2026_708_MOESM15_ESM.zip › Source Data Fig 3/Source Data Fig 3E/YFP.jpg]

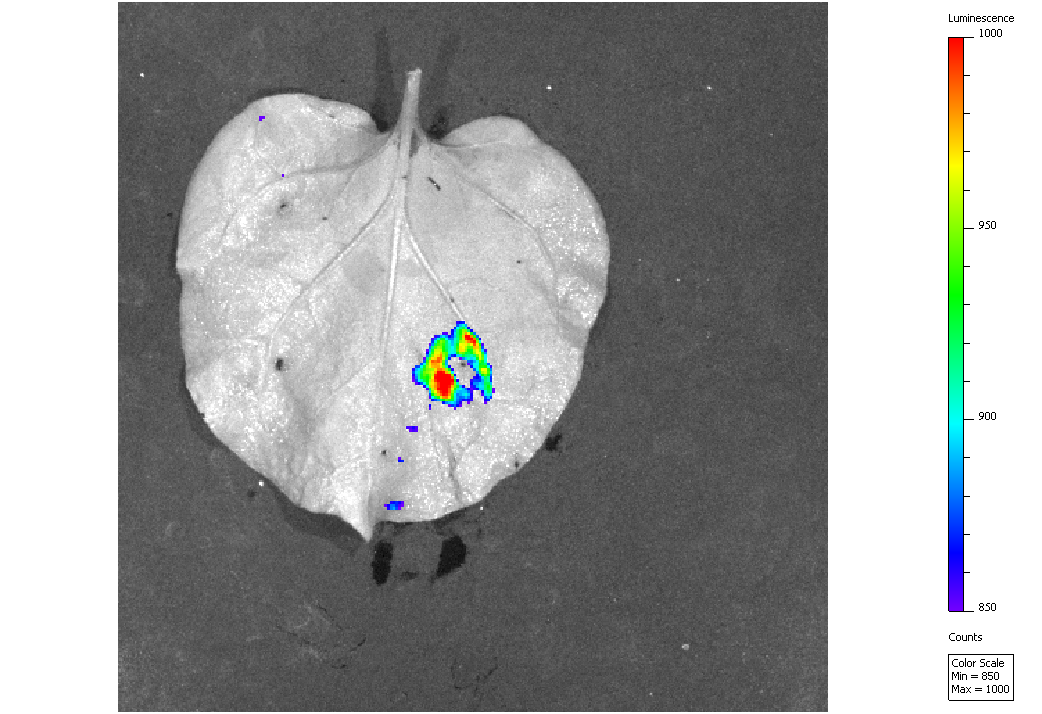

Supplement: Supplementary file 15 — Source data Fig. 3 [file 44318_2026_708_MOESM15_ESM.zip › Source Data Fig 3/Source Data Fig 3F/Source Data Fig 3F.tif]

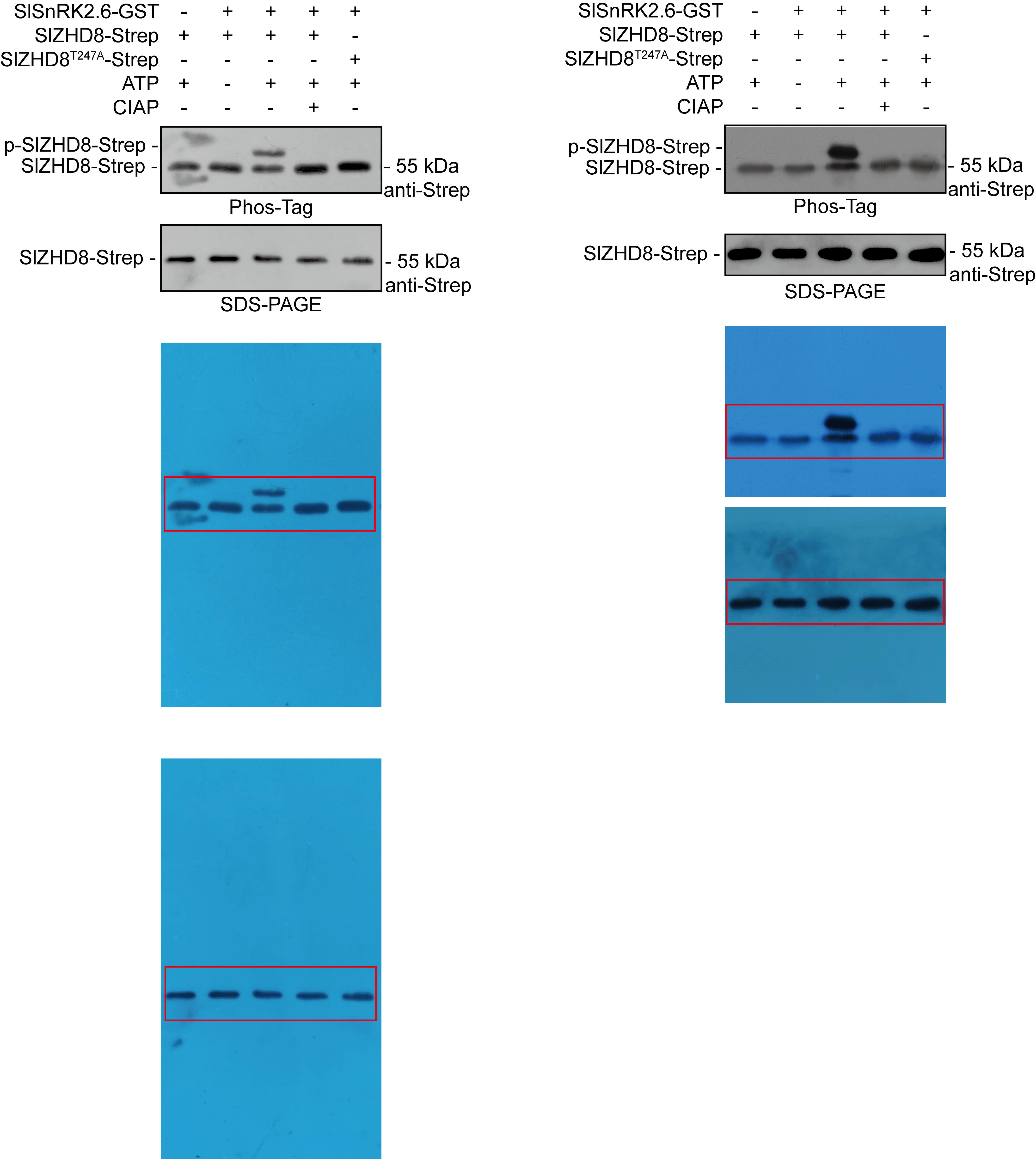

Supplement: Supplementary file 15 — Source data Fig. 3 [file 44318_2026_708_MOESM15_ESM.zip › Source Data Fig 3/Source Data Fig 3G/Source Data Fig 3G.tif]

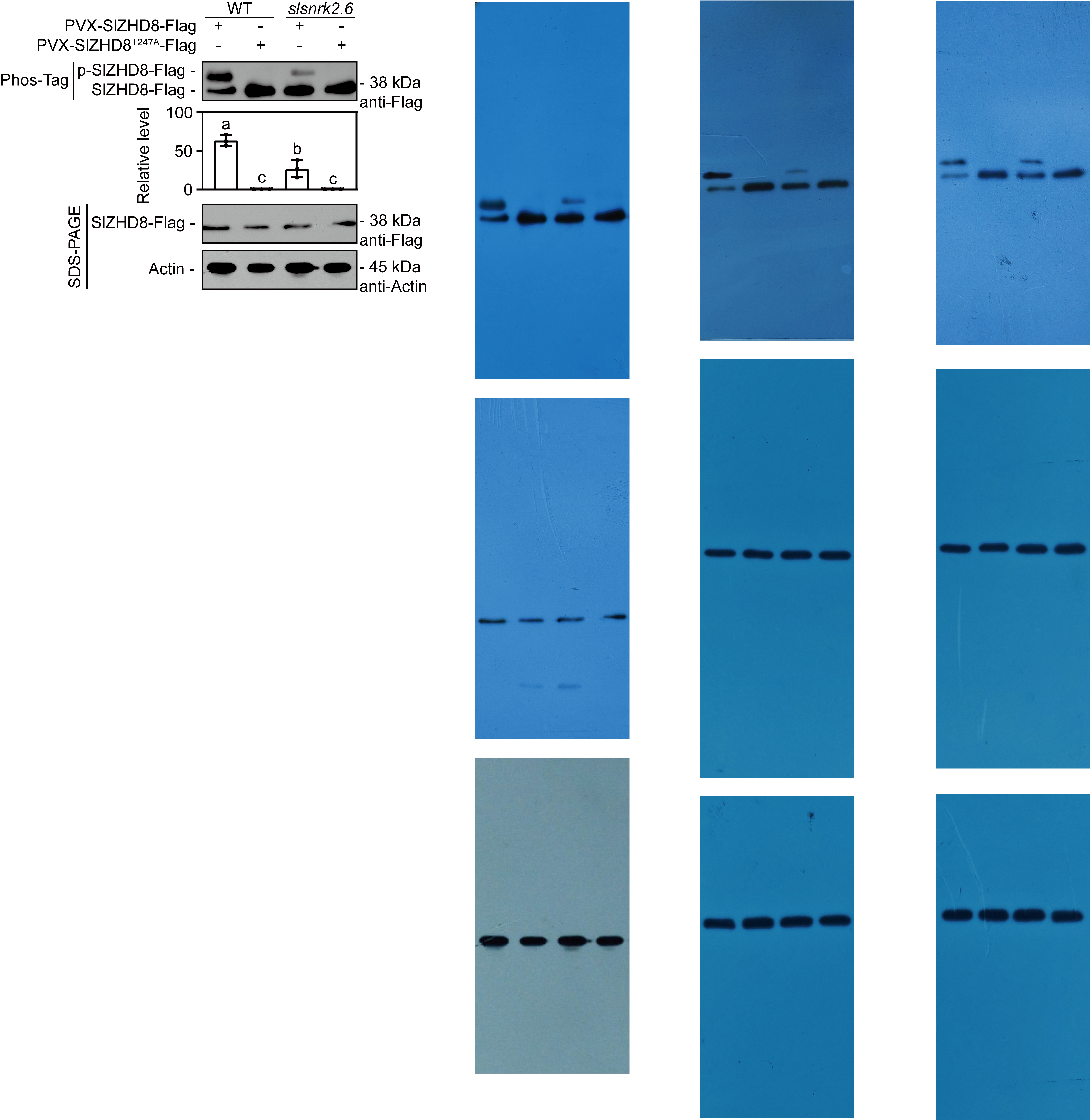

Supplement: Supplementary file 15 — Source data Fig. 3 [file 44318_2026_708_MOESM15_ESM.zip › Source Data Fig 3/Source Data Fig 3H/Fig 3H.tif]

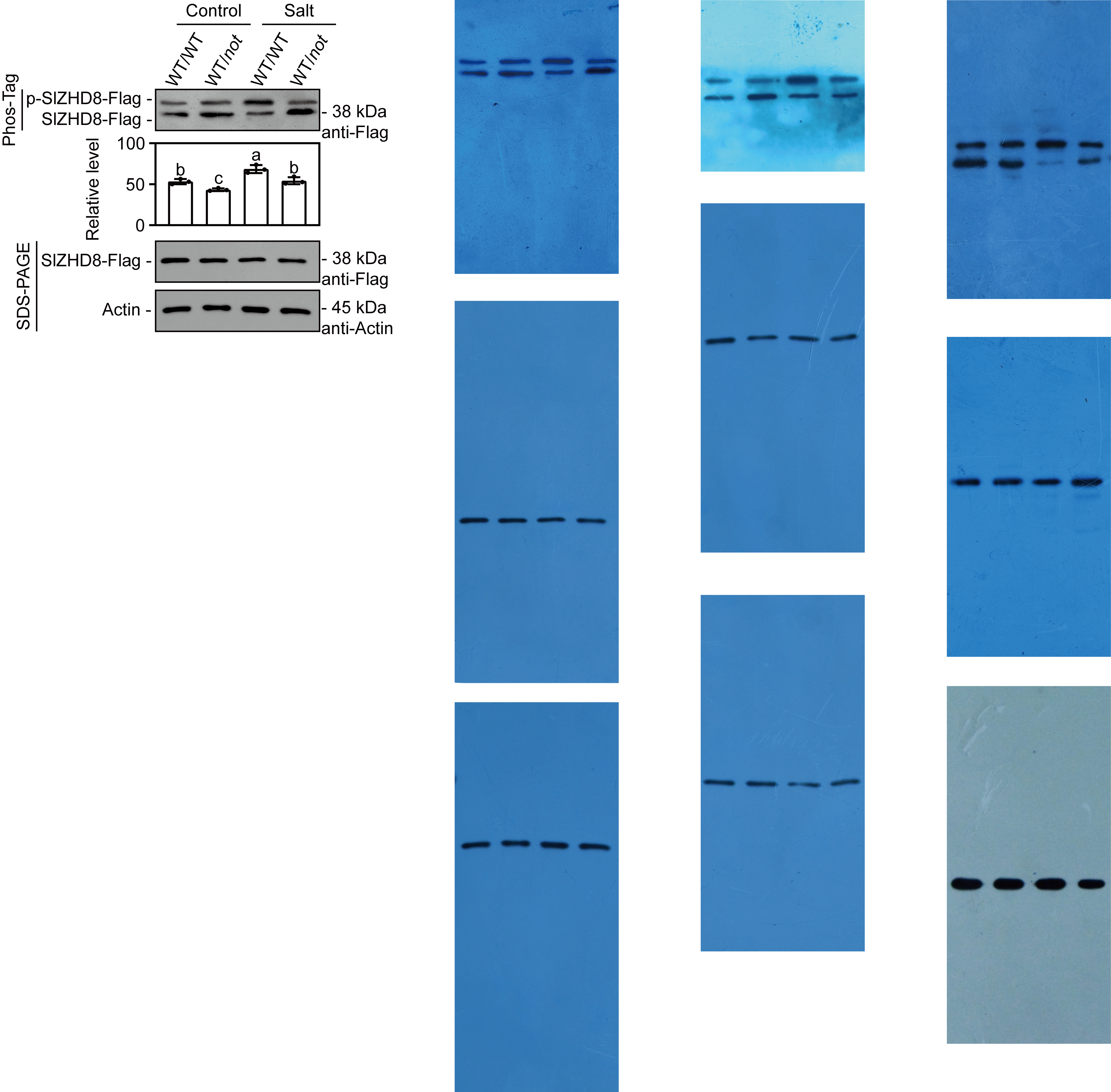

Supplement: Supplementary file 15 — Source data Fig. 3 [file 44318_2026_708_MOESM15_ESM.zip › Source Data Fig 3/Source Data Fig 3I/Fig 3I.tif]

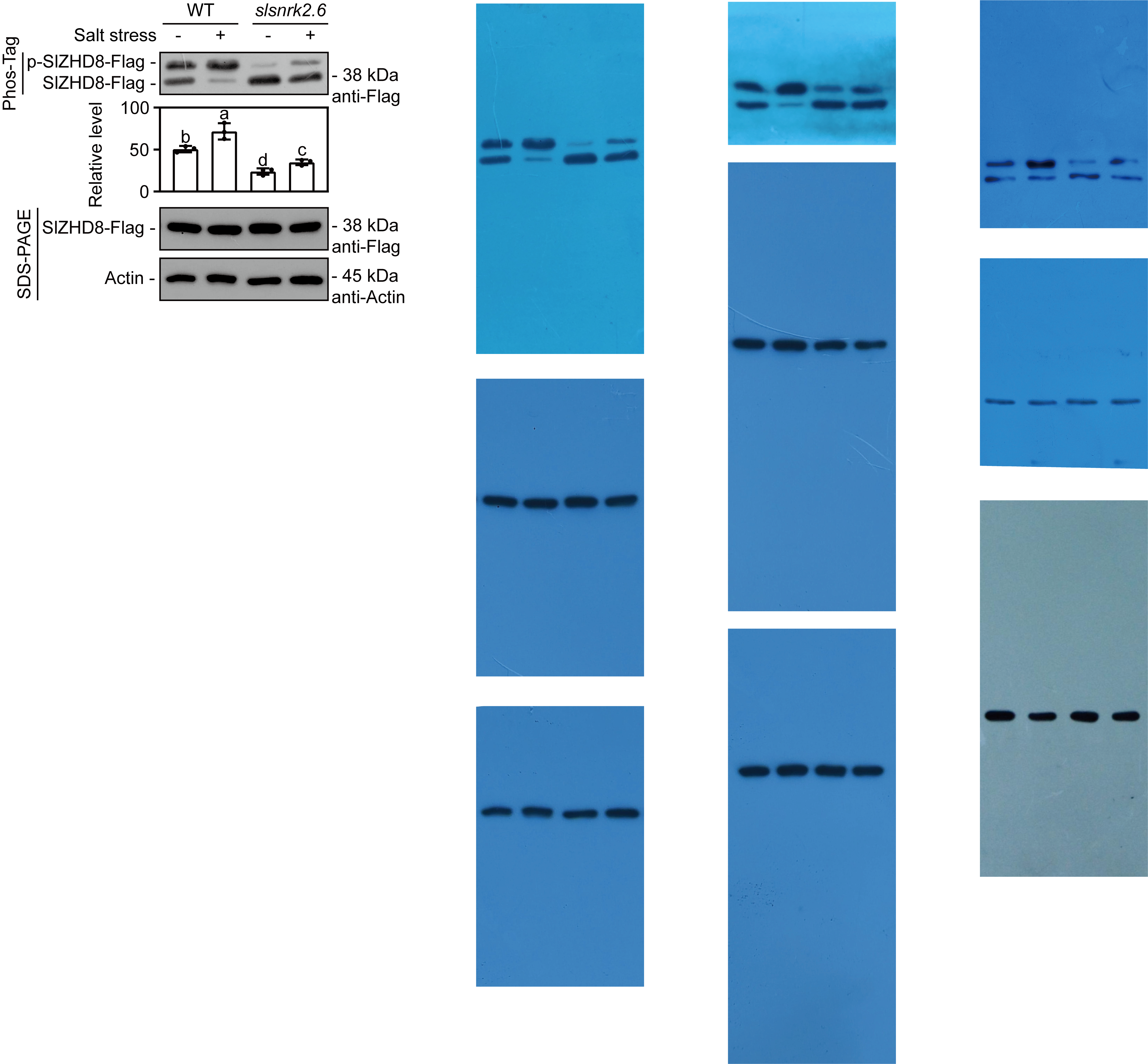

Supplement: Supplementary file 15 — Source data Fig. 3 [file 44318_2026_708_MOESM15_ESM.zip › Source Data Fig 3/Source Data Fig 3J/Fig 3J.tif]

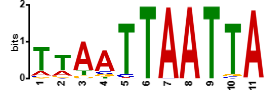

Supplement: Supplementary file 16 — Source data Fig. 4 [file 44318_2026_708_MOESM16_ESM.zip › Source Data Fig 4/Source Data Fig 4C/Fig 4C-motif.png]

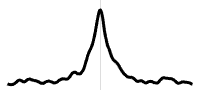

Supplement: Supplementary file 16 — Source data Fig. 4 [file 44318_2026_708_MOESM16_ESM.zip › Source Data Fig 4/Source Data Fig 4C/Fig 4C-value.png]

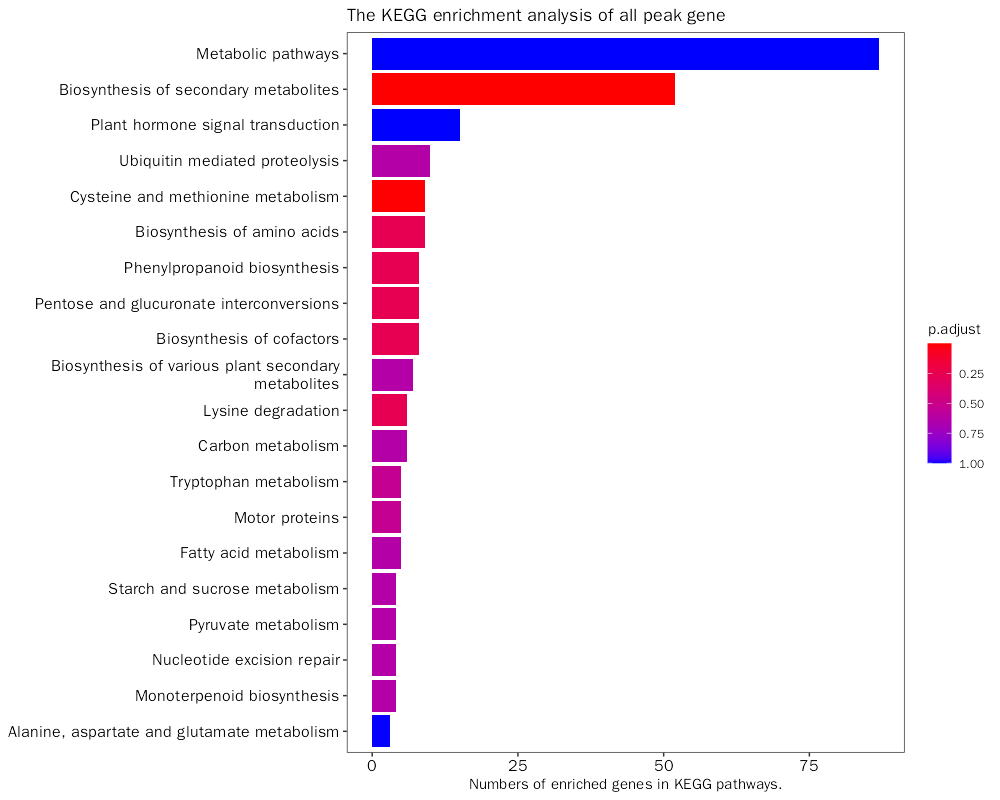

Supplement: Supplementary file 16 — Source data Fig. 4 [file 44318_2026_708_MOESM16_ESM.zip › Source Data Fig 4/Source Data Fig 4D/Fig 4D.png]

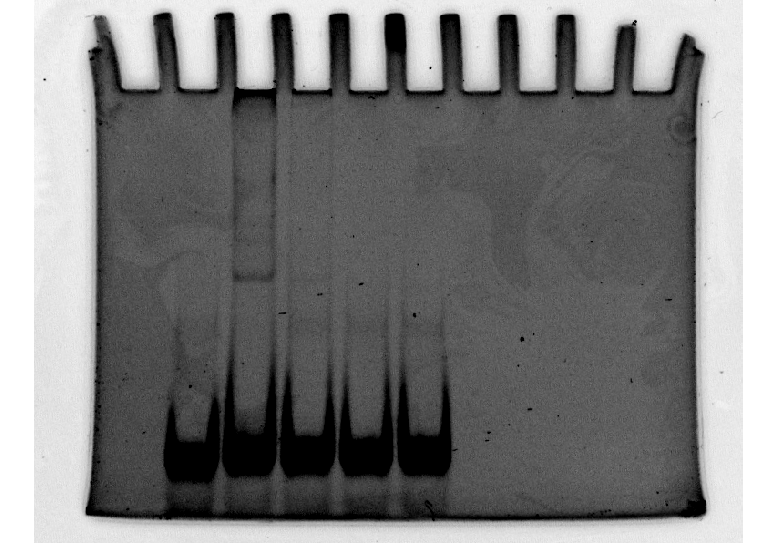

Supplement: Supplementary file 16 — Source data Fig. 4 [file 44318_2026_708_MOESM16_ESM.zip › Source Data Fig 4/Source Data Fig 4I/SlZHD8-His and SlSUS3 promoter.png]

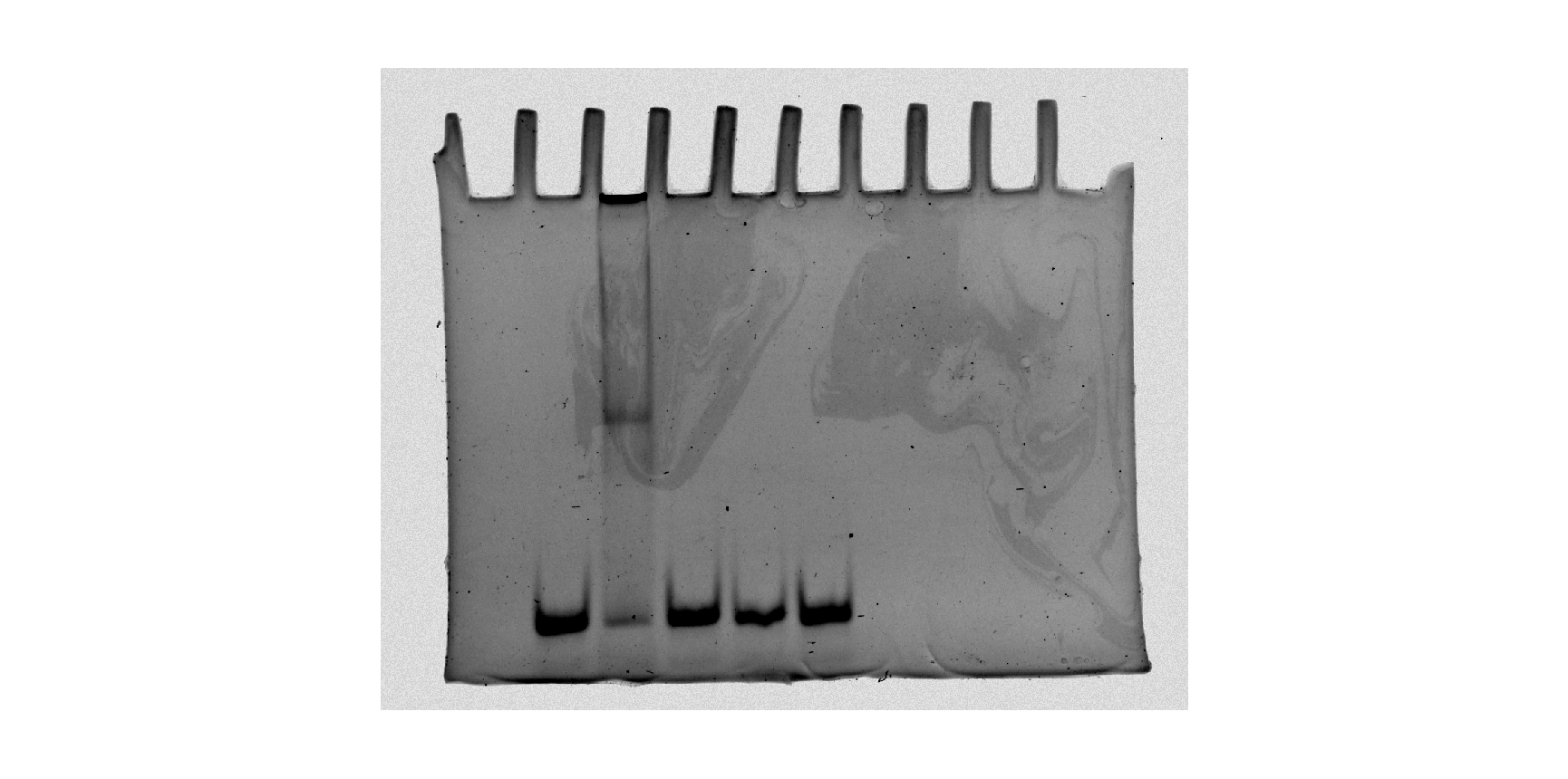

Supplement: Supplementary file 16 — Source data Fig. 4 [file 44318_2026_708_MOESM16_ESM.zip › Source Data Fig 4/Source Data Fig 4I/SlZHD8-His and SlSWEET12 promoter.png]

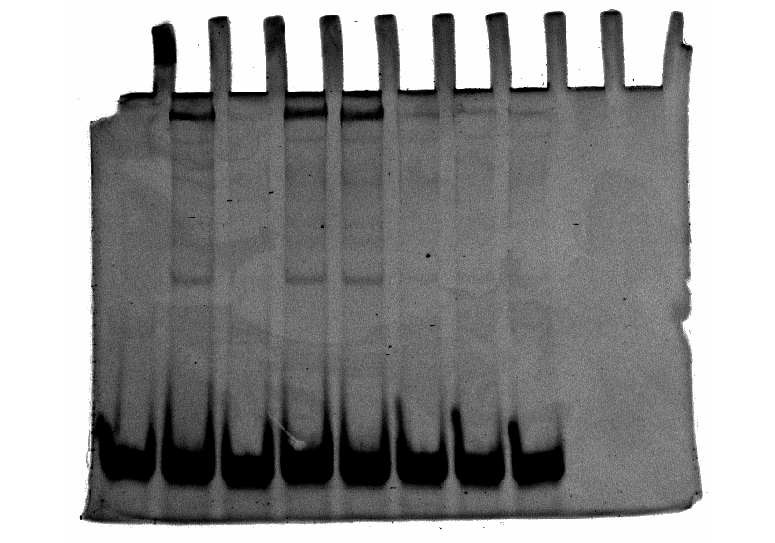

Supplement: Supplementary file 16 — Source data Fig. 4 [file 44318_2026_708_MOESM16_ESM.zip › Source Data Fig 4/Source Data Fig 4J/SlZHD8-His and SlSUS3 probe.png]

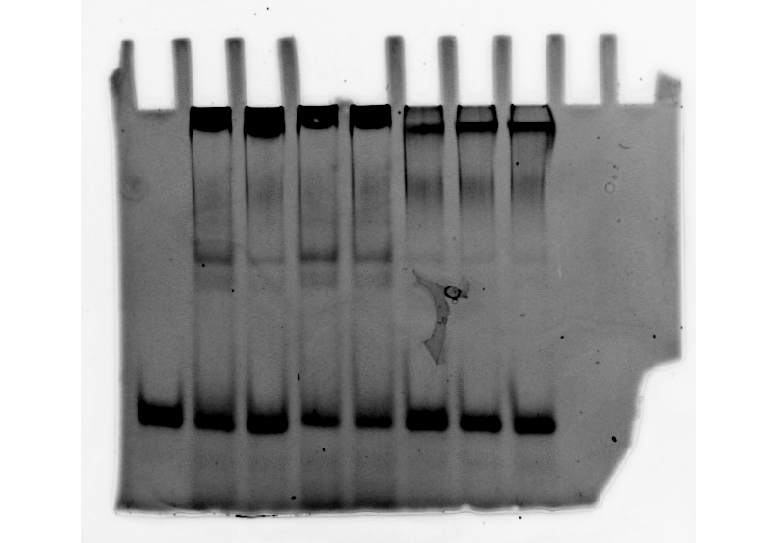

Supplement: Supplementary file 16 — Source data Fig. 4 [file 44318_2026_708_MOESM16_ESM.zip › Source Data Fig 4/Source Data Fig 4J/SlZHD8-His and SlSWEET12 probe.png]

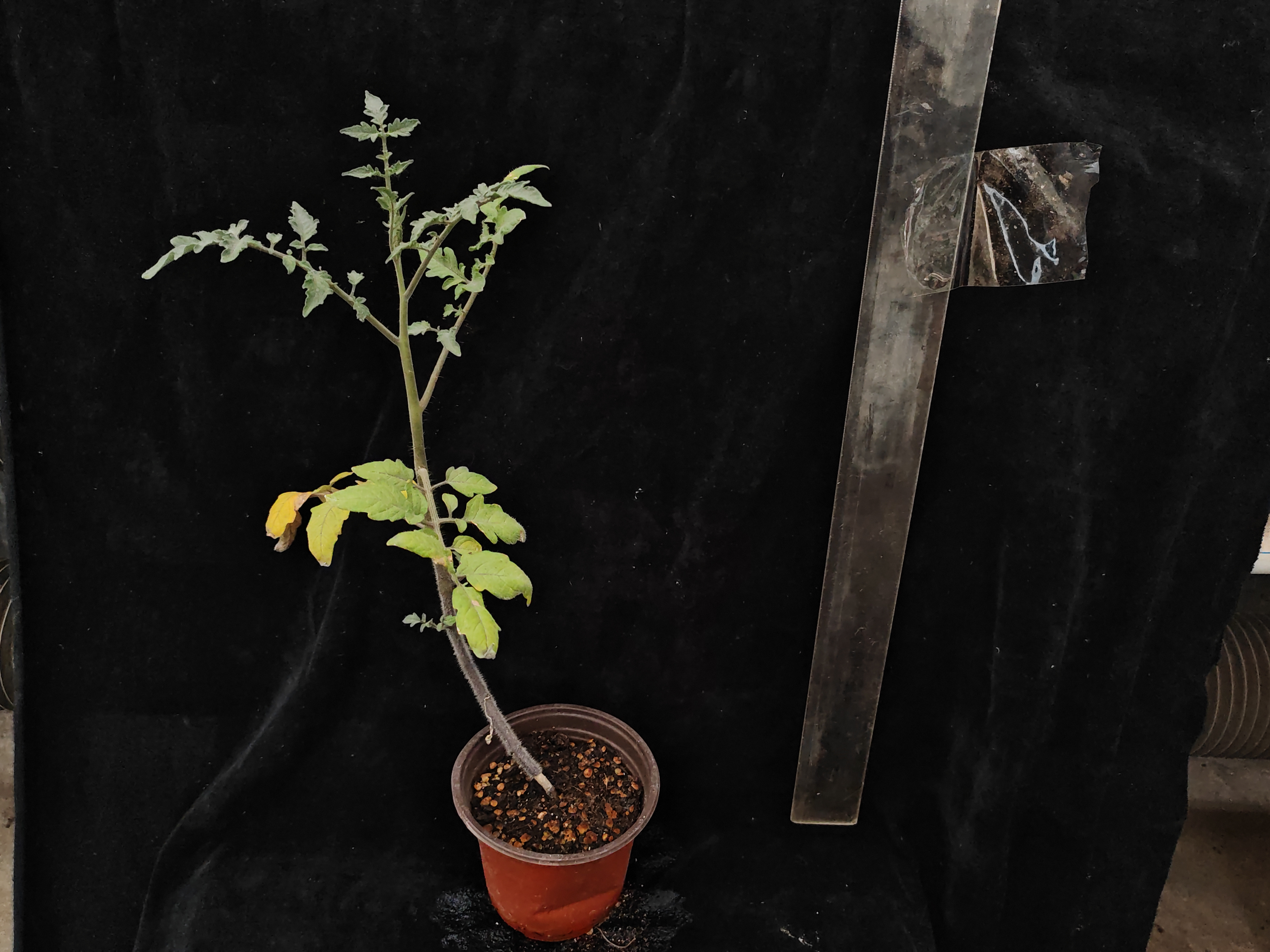

Supplement: Supplementary file 17 — Source data Fig. 5 [file 44318_2026_708_MOESM17_ESM.zip › Source Data Fig 5/Source Data Fig 5G/1 WT.jpg]

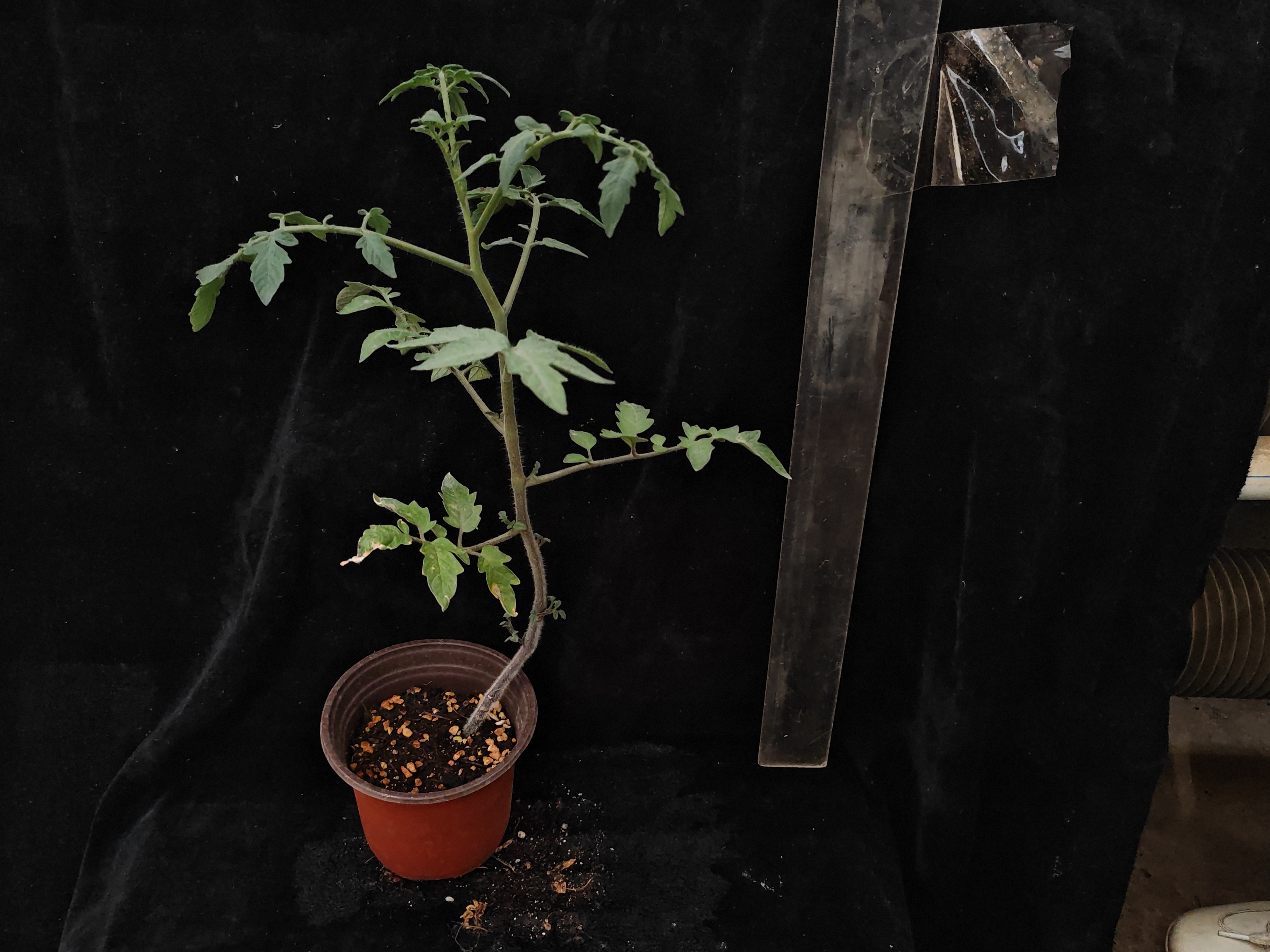

Supplement: Supplementary file 17 — Source data Fig. 5 [file 44318_2026_708_MOESM17_ESM.zip › Source Data Fig 5/Source Data Fig 5G/2 slzhd8.jpg]

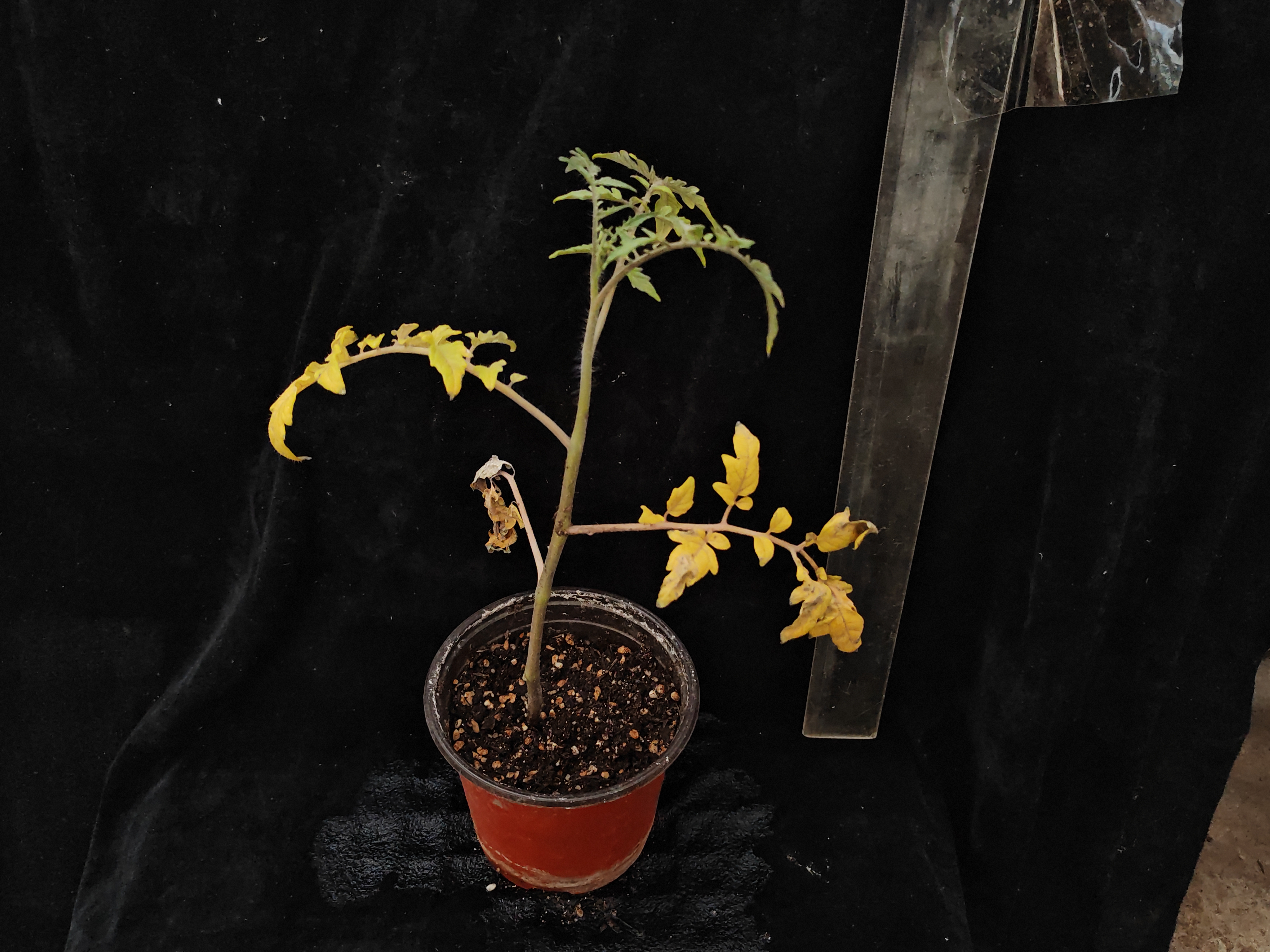

Supplement: Supplementary file 17 — Source data Fig. 5 [file 44318_2026_708_MOESM17_ESM.zip › Source Data Fig 5/Source Data Fig 5G/3 slsweet2.jpg]

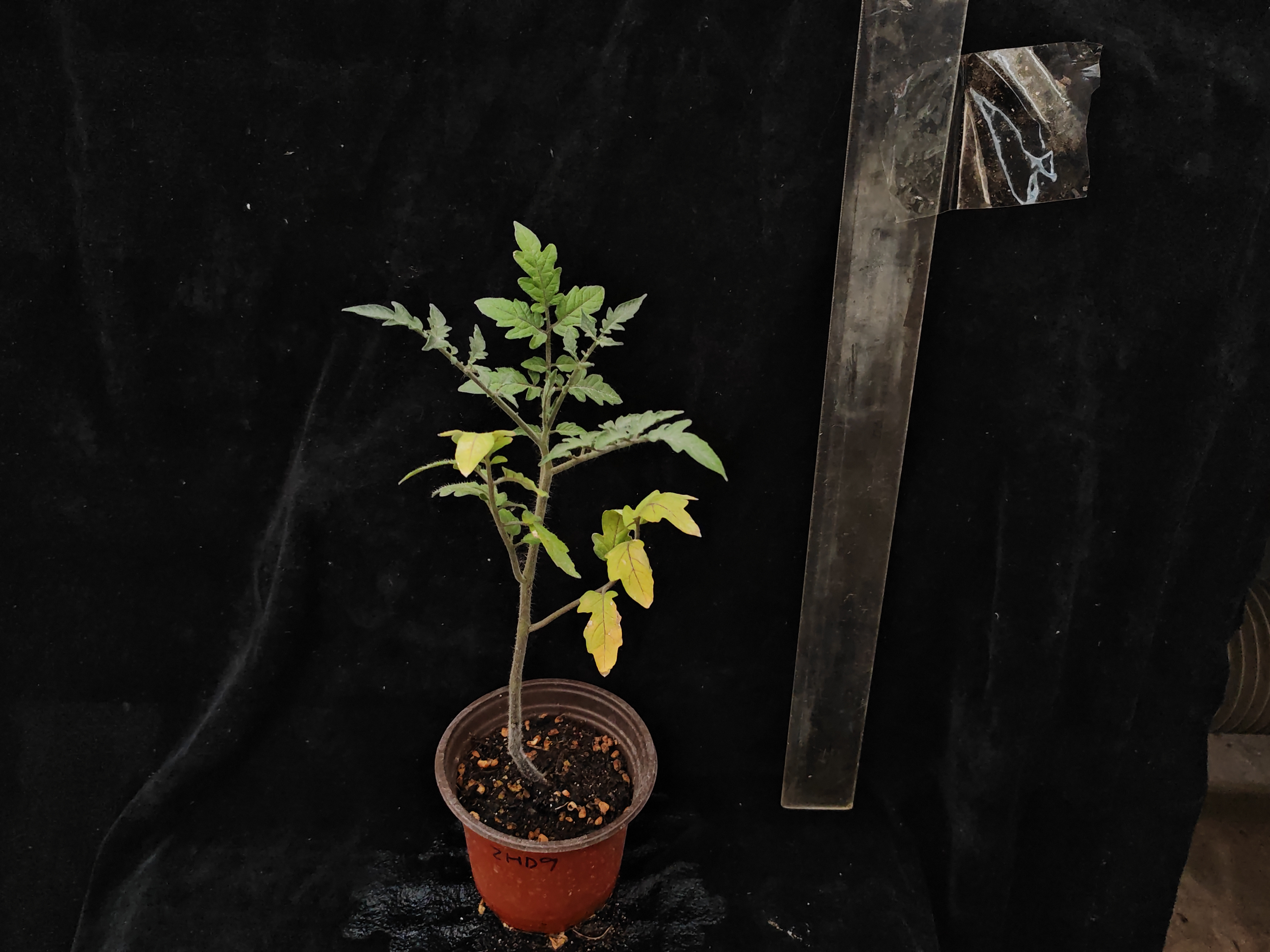

Supplement: Supplementary file 17 — Source data Fig. 5 [file 44318_2026_708_MOESM17_ESM.zip › Source Data Fig 5/Source Data Fig 5G/4 slzhd8 slsweet12.jpg]

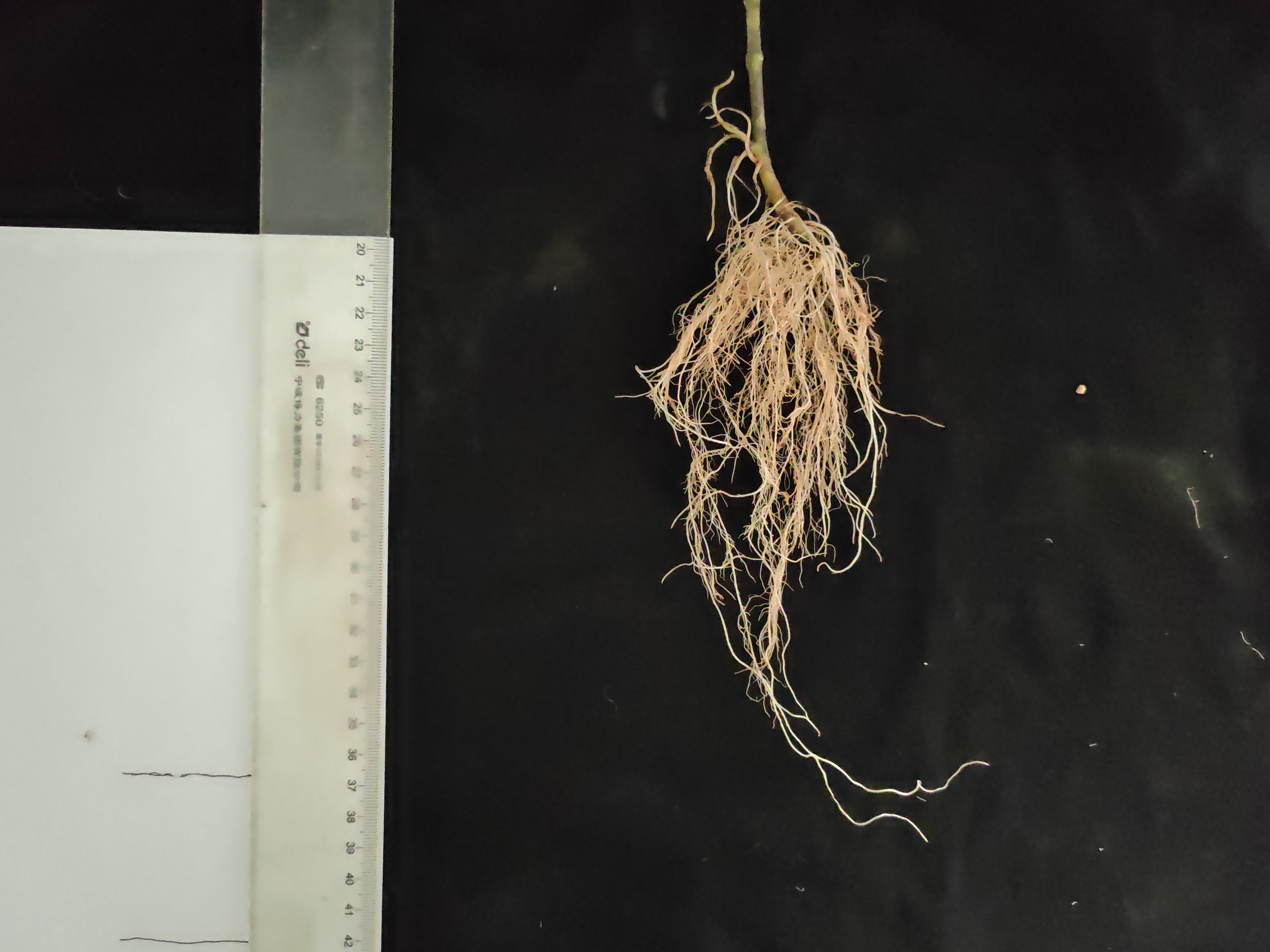

Supplement: Supplementary file 17 — Source data Fig. 5 [file 44318_2026_708_MOESM17_ESM.zip › Source Data Fig 5/Source Data Fig 5G/Root 1.jpg]

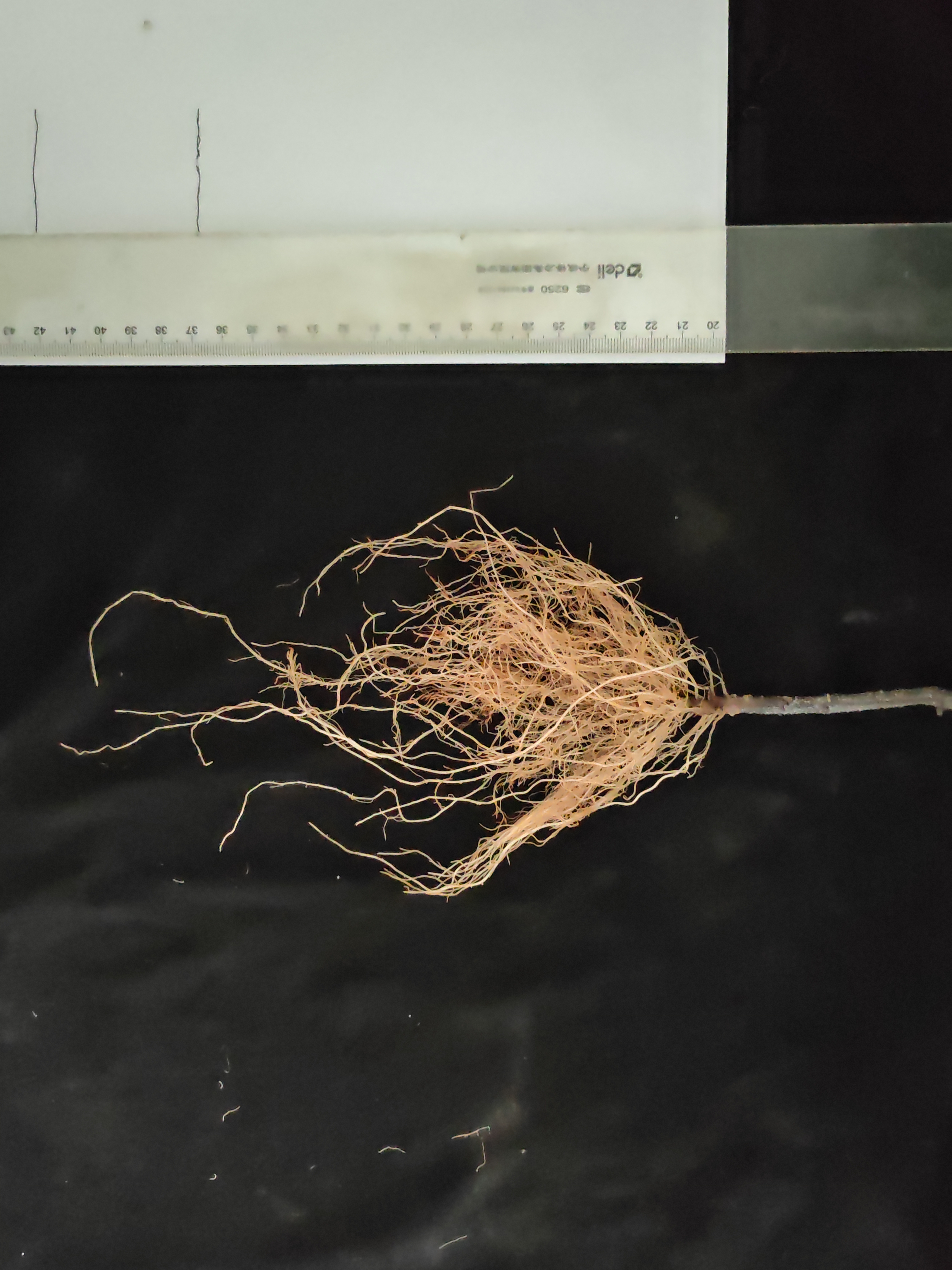

Supplement: Supplementary file 17 — Source data Fig. 5 [file 44318_2026_708_MOESM17_ESM.zip › Source Data Fig 5/Source Data Fig 5G/Root 2.jpg]

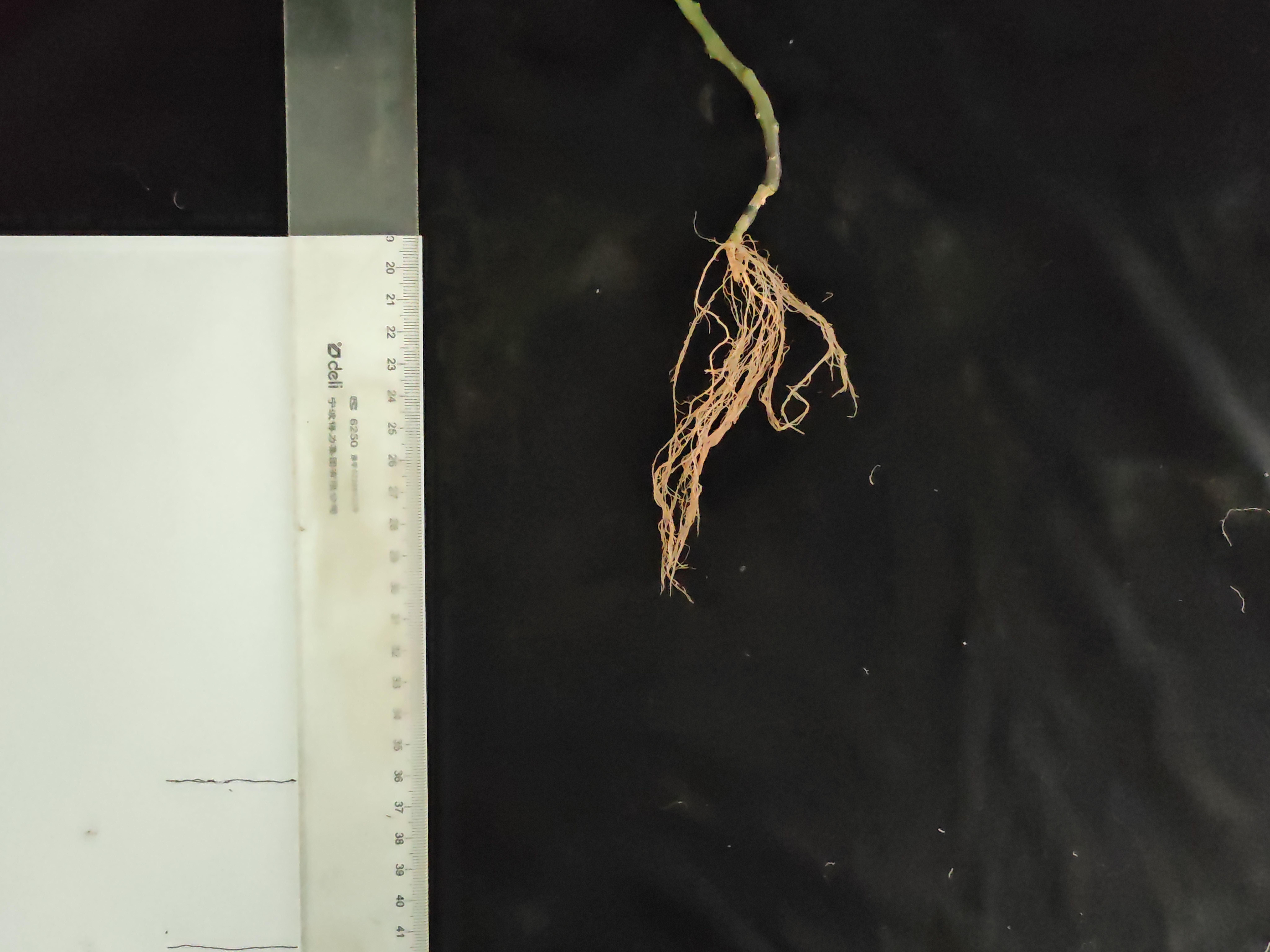

Supplement: Supplementary file 17 — Source data Fig. 5 [file 44318_2026_708_MOESM17_ESM.zip › Source Data Fig 5/Source Data Fig 5G/Root 3.jpg]

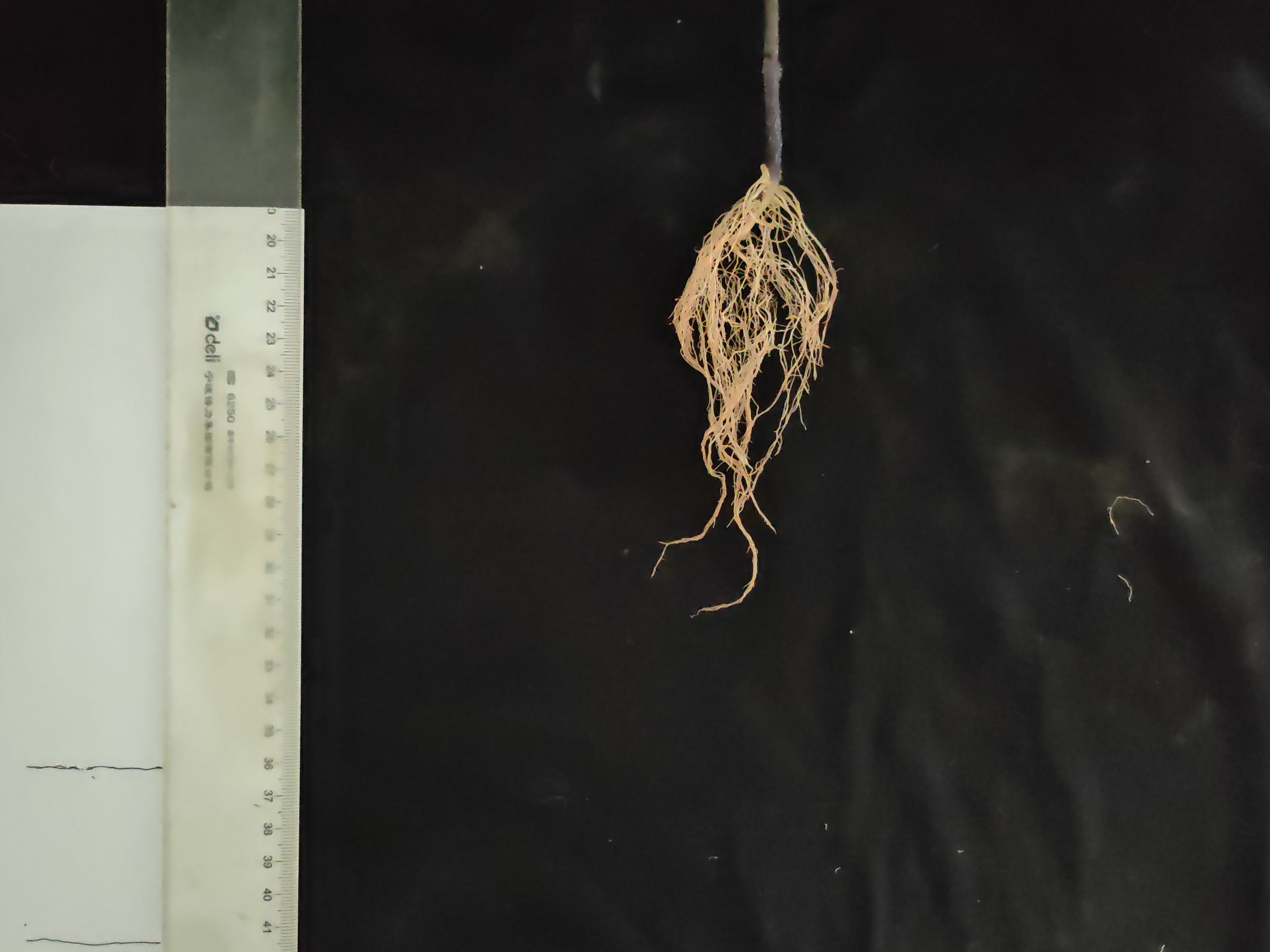

Supplement: Supplementary file 17 — Source data Fig. 5 [file 44318_2026_708_MOESM17_ESM.zip › Source Data Fig 5/Source Data Fig 5G/Root 4.jpg]

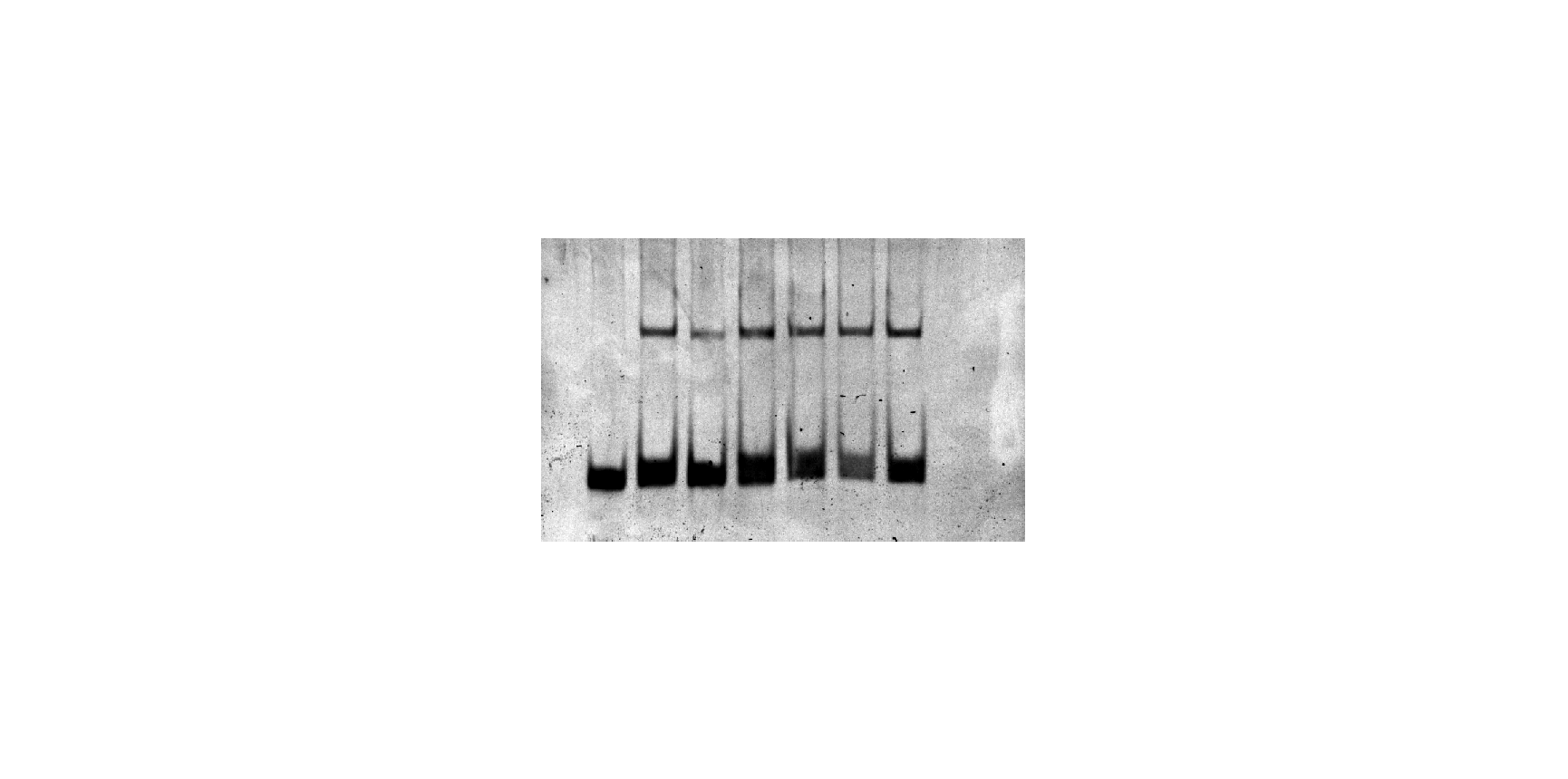

Supplement: Supplementary file 18 — Source data Fig. 6 [file 44318_2026_708_MOESM18_ESM.zip › Source Data Fig 6/Source Data Fig 6C/SlSUS3.tif]

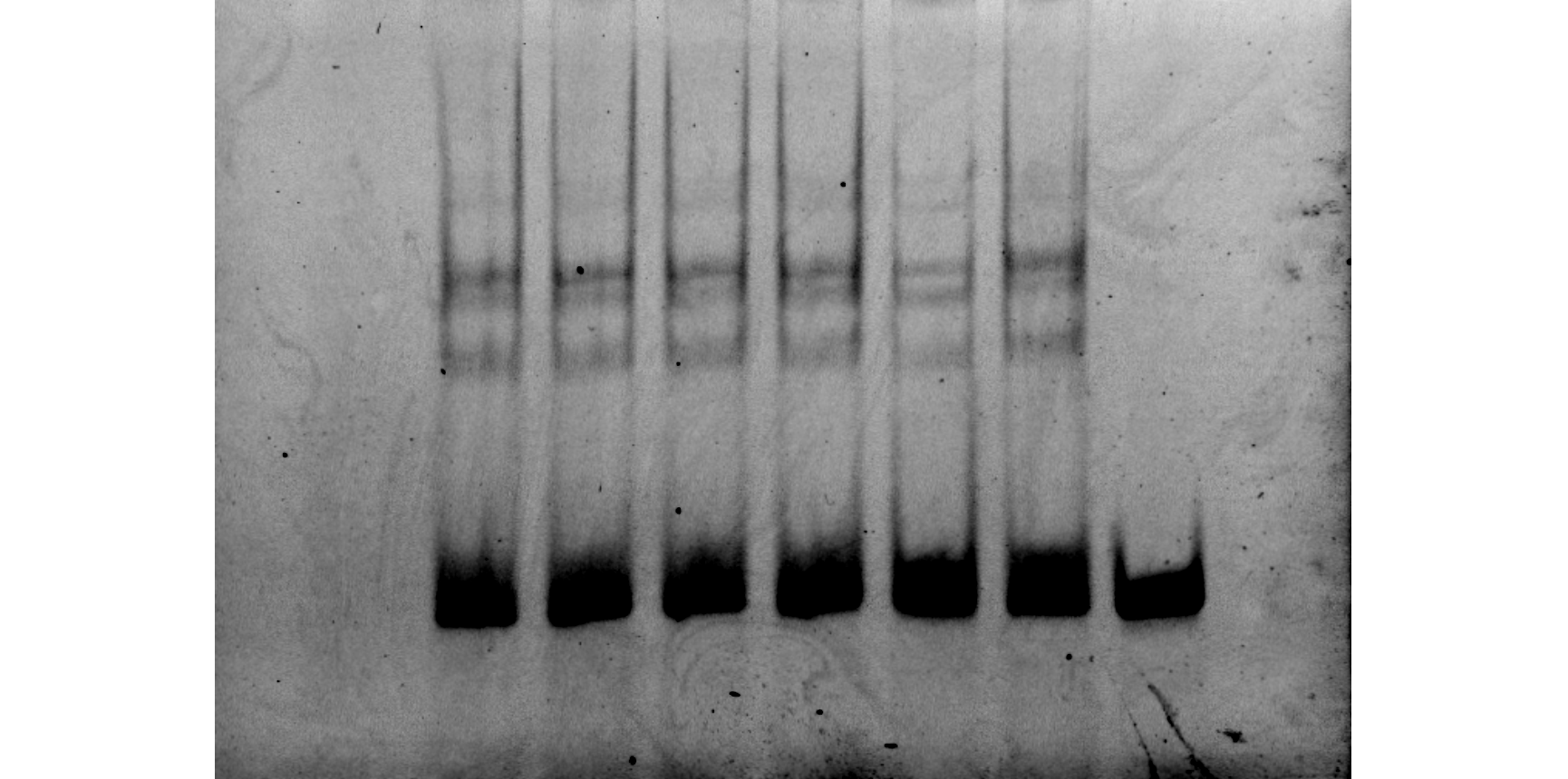

Supplement: Supplementary file 18 — Source data Fig. 6 [file 44318_2026_708_MOESM18_ESM.zip › Source Data Fig 6/Source Data Fig 6C/SlSWEET12.tif]

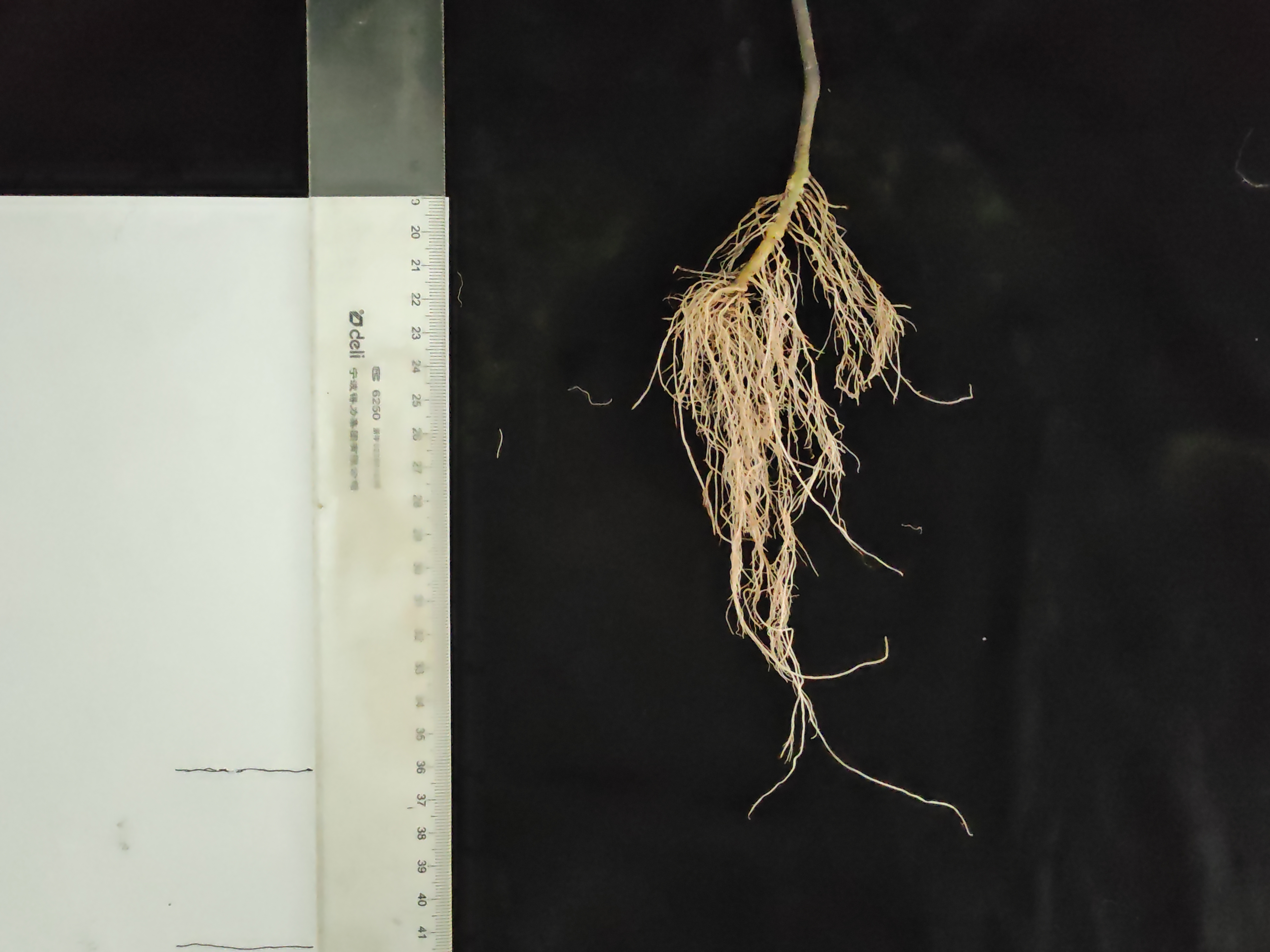

Supplement: Supplementary file 18 — Source data Fig. 6 [file 44318_2026_708_MOESM18_ESM.zip › Source Data Fig 6/Source Data Fig 6K/Hap A-root.jpg]

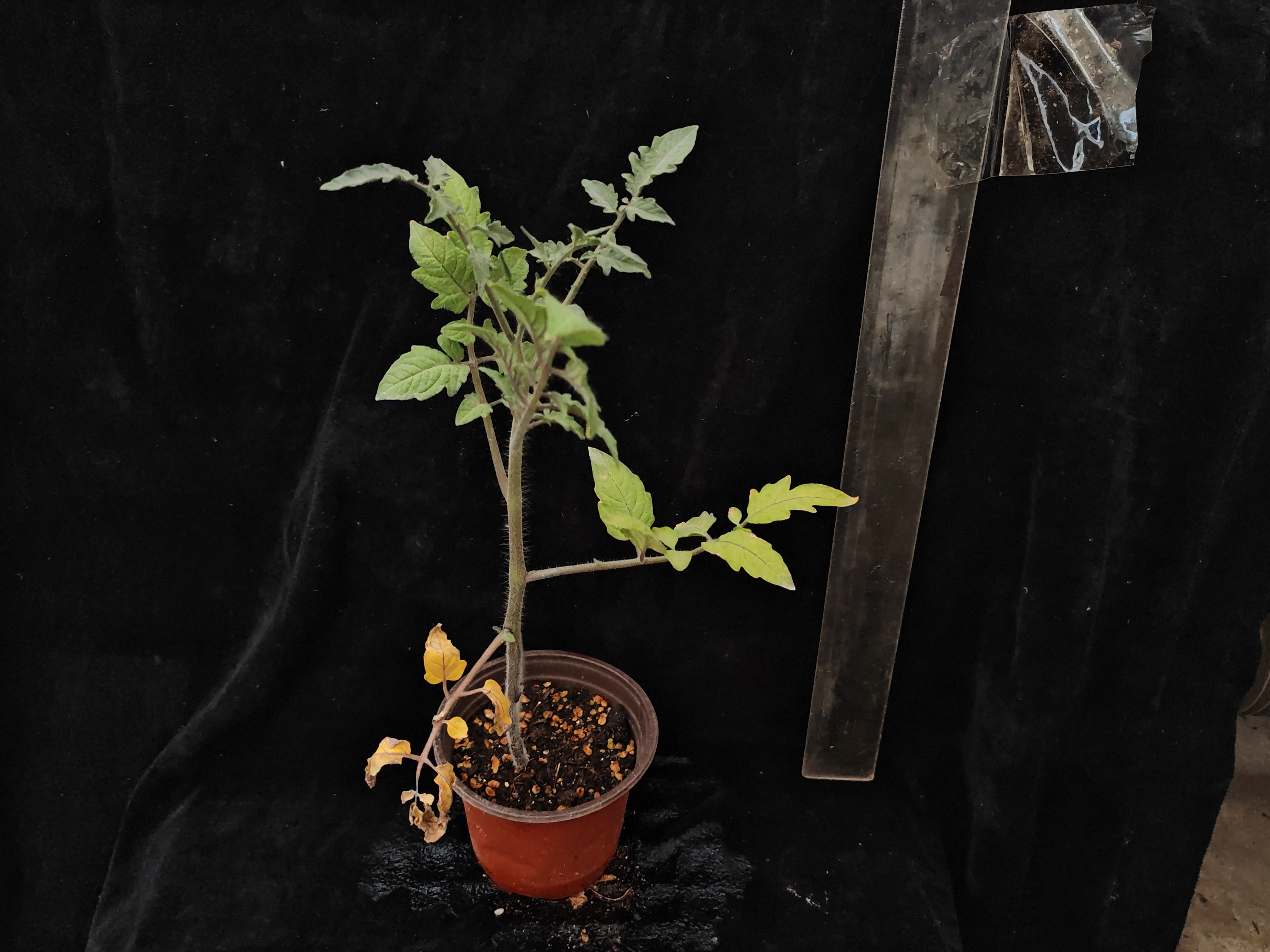

Supplement: Supplementary file 18 — Source data Fig. 6 [file 44318_2026_708_MOESM18_ESM.zip › Source Data Fig 6/Source Data Fig 6K/Hap A-shoot.jpg]

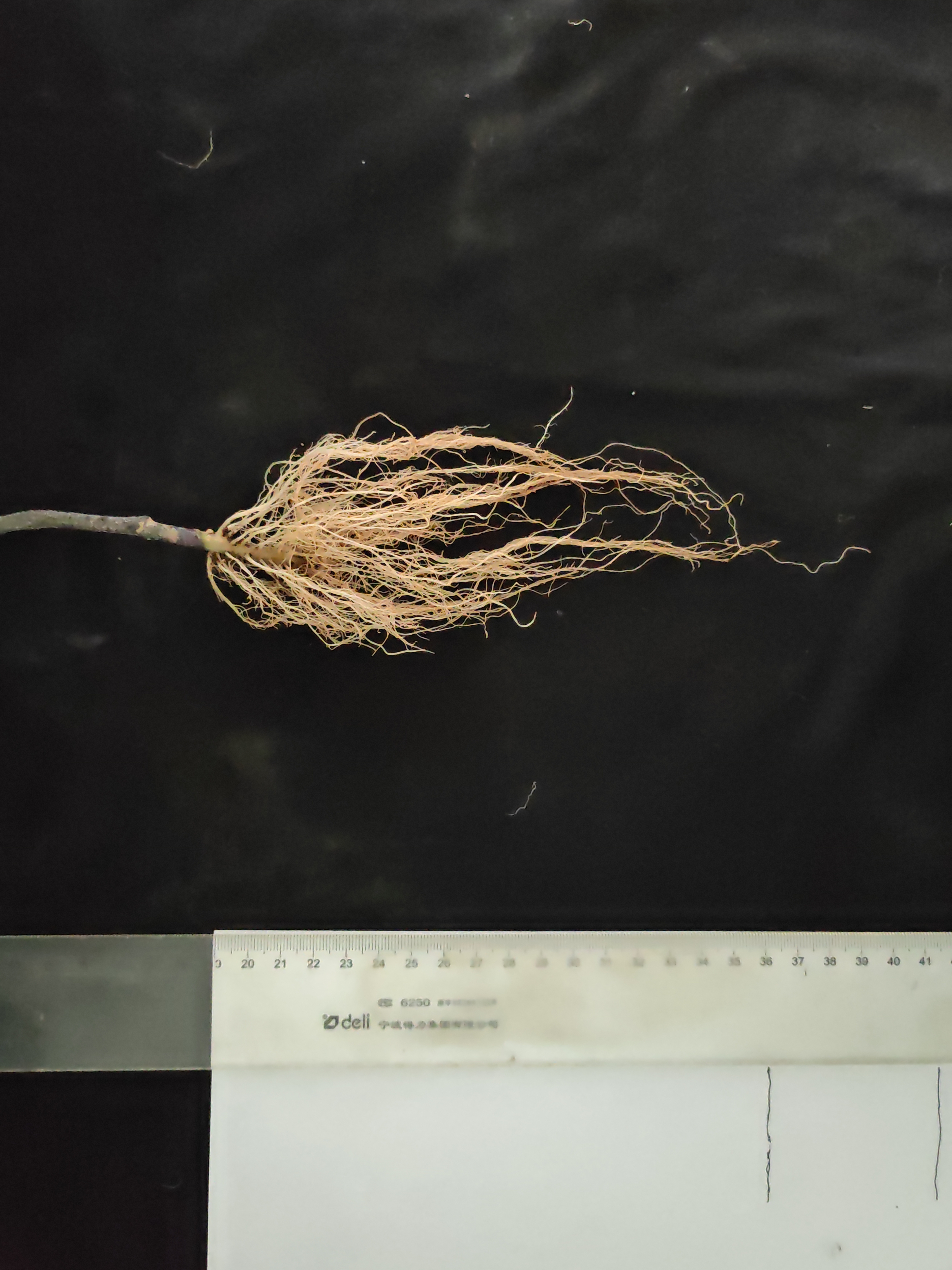

Supplement: Supplementary file 18 — Source data Fig. 6 [file 44318_2026_708_MOESM18_ESM.zip › Source Data Fig 6/Source Data Fig 6K/Hap B-root.jpg]

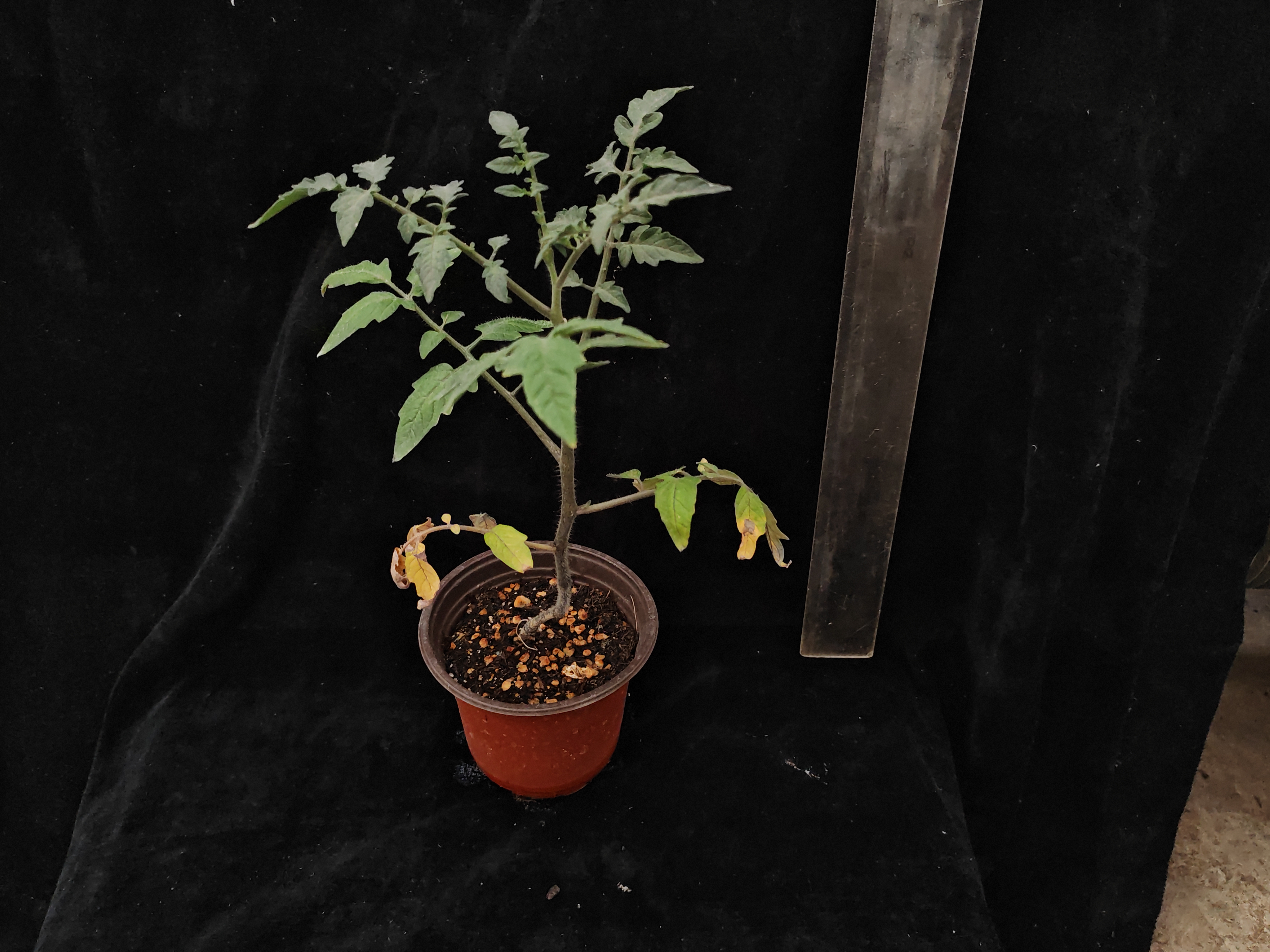

Supplement: Supplementary file 18 — Source data Fig. 6 [file 44318_2026_708_MOESM18_ESM.zip › Source Data Fig 6/Source Data Fig 6K/Hap B-shoot.jpg]

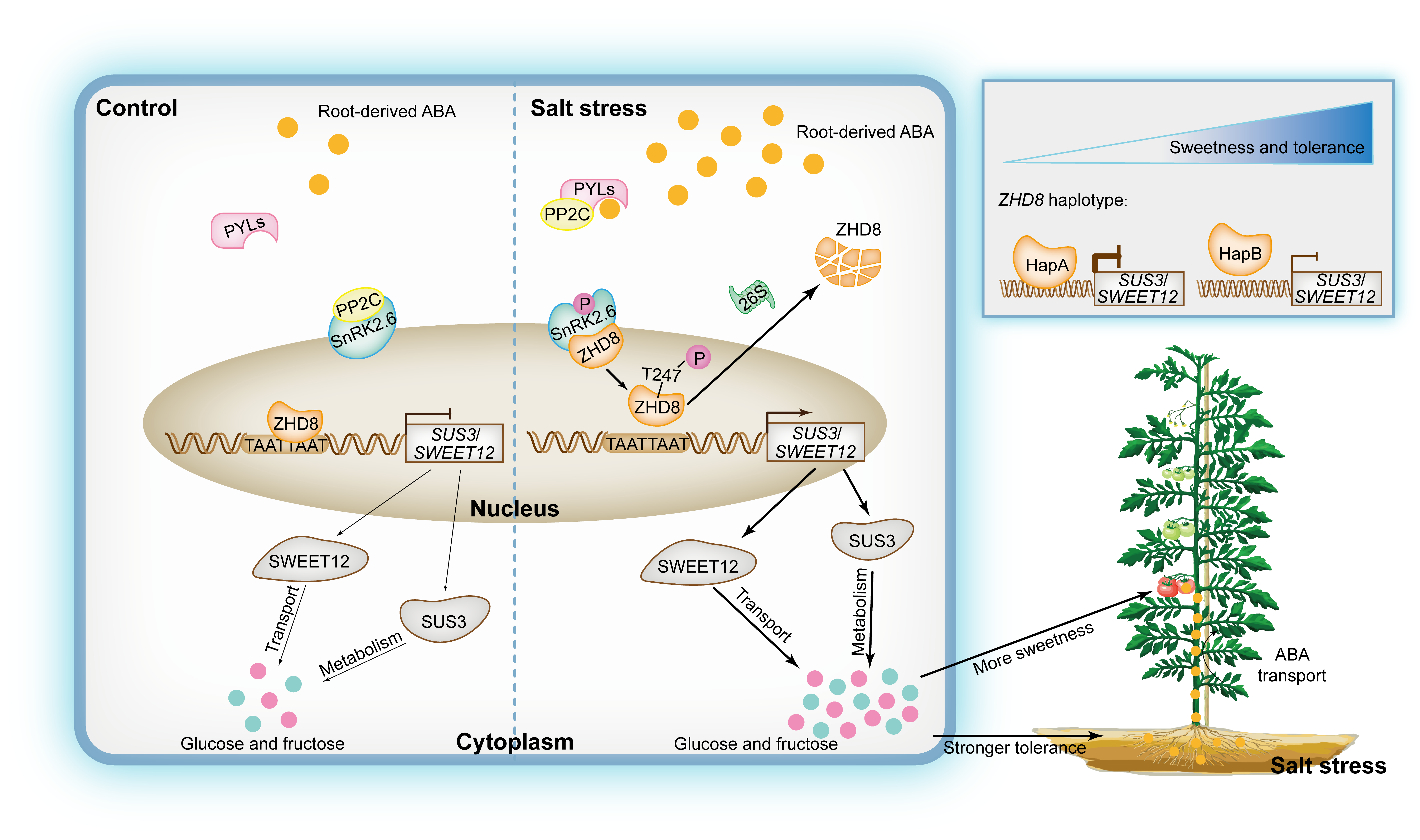

Supplement: Supplementary file 19 — Source data Fig. 7 [file 44318_2026_708_MOESM19_ESM.zip › Source Data Fig 7/Fig 7.tif]
